# Supplementary material for: Probing the origins of human acetylcholinesterase inhibition via QSAR modeling and molecular docking
Source: PeerJ. 2016 Aug 9;4:e2322. doi: 10.7717/peerj.2322 (PMC4991866; doi:10.7717/peerj.2322)
Supplement: Table S1 [file peerj-04-2322-s001.docx]

**Table S1.** Outlying compounds as deduced from the Williams plot.

| **Compound No.** | **Structure** |
| --- | --- |
| 997 |  |
| 1829 |  |
| 62 |  |
| 1096 |  |
| 13 |  |
| 677 |  |
| 1567 |  |
| 576 |  |
| 1644 |  |
| 1098 |  |
| 2022 |  |
| 1447 |  |
| 322 |  |
| 2116 |  |
| 2120 |  |
| 2388 |  |
| 2117 |  |
| 2392 |  |
| 2323 |  |
| 2423 |  |
| 2424 |  |
| 2507 |  |
| 2219 |  |
| 2422 |  |
| 2486 |  |
| 2353 |  |
| 2130 |  |
| 2553 |  |
| 2389 |  |
| 2072 |  |
| 2103 |  |
| 2125 |  |
