# Supplementary material for: Probing the origins of human acetylcholinesterase inhibition via QSAR modeling and molecular docking
Source: PeerJ. 2016 Aug 9;4:e2322. doi: 10.7717/peerj.2322 (PMC4991866; doi:10.7717/peerj.2322)

Visual representation of Lipinski's descriptors

Saw Simeon, Nuttapat Anuwongcharoen, Watshara Shoombuatong, Aijaz Ahmad Malik, Virapong Prachayasittikul, Jarl E. S. Wikberg and Chanin Nantasenamat

June 7, 2016

Chemical space of AChE inhibitors

Convert IC50 to pIC50 as well as binning the activity (i.e., Active, Inactive and Intermediate)

```
data <- read.csv("Lipinski_descriptors.csv")
IC50_nm <- data$STANDARD_VALUE
IC50 <- as.numeric(IC50_nm)*10^-9
pIC50 <- -log10(IC50)
descriptors_name <- c("ALogP", "nHBAcc", "nHBDon", "MW")
descriptors <- data[, descriptors_name]
#data_classification <- cbind(pIC50, descriptors)
Activity <- ifelse(pIC50 > 6, "Active",
                      ifelse((pIC50 <= 6) & (pIC50 < 5), "Inactive",
                             "Intermediate"))
Activity <- data.frame(Activity)
```

Visualization of Lipinski's descriptors

Chemical space of AChE inhibitors are shown as active (green), inactive (red) and intermediate (blue)

```
library(ggplot2)
library(cowplot)
data_2 <- cbind(Activity, descriptors)
data_2 <- na.omit(data_2)
data_plot <- data.frame(data_2)
p <- ggplot(data_plot, aes(MW, ALogP))
p <- p + geom_point(aes(colour = factor(Activity)), size = 3, alpha = 0.3)
p <- p + theme(legend.position = ("none"),
           panel.border = element_rect(linetype = "solid",
                                        colour = "black", fill = NA, size = 1),
           axis.text.x = element_text(colour = "black", size = 10),
           axis.text.y = element_text(colour = "black", size = 10),
           plot.margin = unit(c(1, 1, 1, 1), "cm"),
           axis.title.x = element_text(colour = "black", size = 15, face = "bold"),
           axis.title.y = element_text(colour = "black", size = 15, face = "bold")
           )
print(p)
```

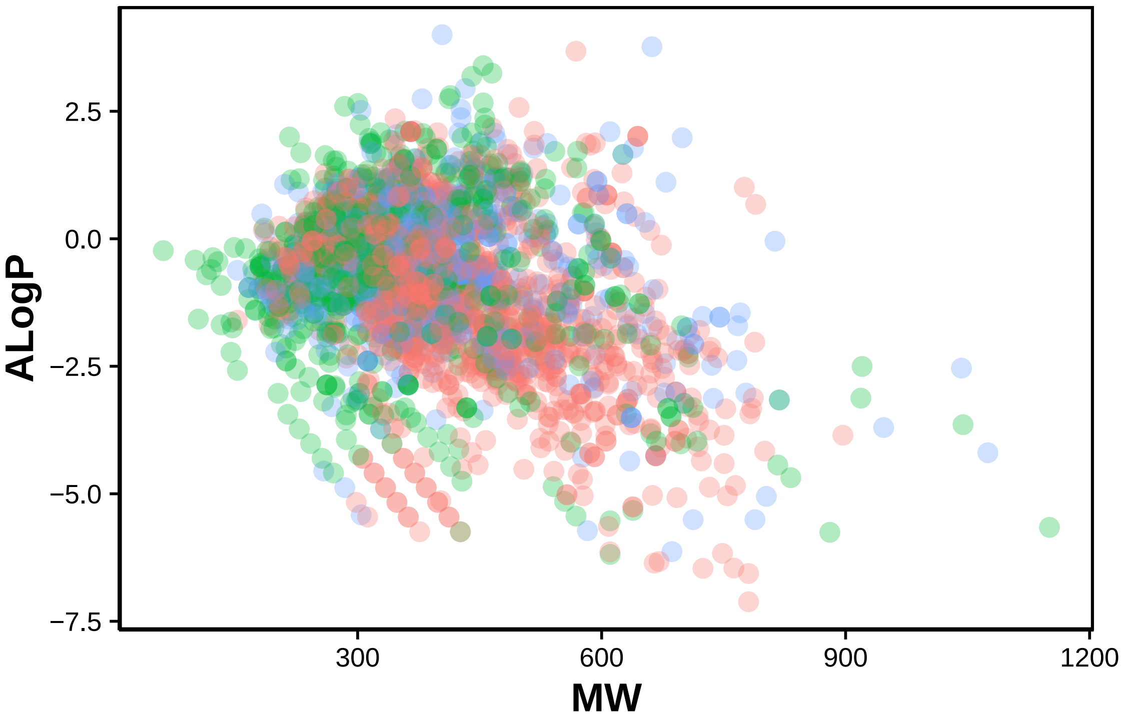

Boxplot of Lipinski's rule-of-five descriptors (ALogP)

```
p_1 <- ggplot(data_plot, aes(factor(Activity), ALogP))
p_1 <- p_1 + geom_boxplot(aes(fill = factor(Activity)), alpha = 0.7)
p_1 <- p_1 + theme(legend.position = ("none"),
                   panel.border = element_rect(linetype = "solid",
                                               colour = "black", fill = NA, size = 1),
                   axis.text.x = element_text(colour = "black", size = 15),
                   axis.text.y = element_text(colour = "black", size = 15),
                   plot.margin = unit(c(1, 1, 1, 1), "cm"),
                   axis.title.y = element_text(size = 20, face = "bold"),
                   axis.title.x = element_blank())

print(p_1)
```

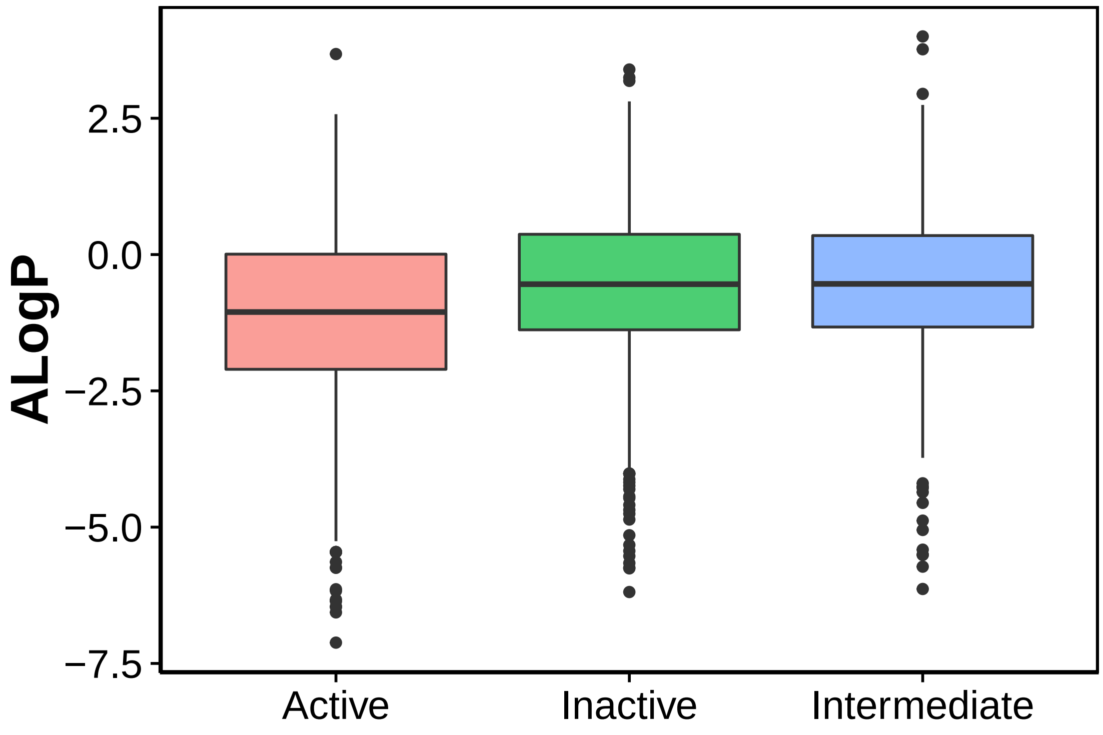

Boxplot of Lipinski's rule-of-five descriptors (nHBAcc)

```
p_2 <- ggplot(data_plot, aes(factor(Activity), nHBAcc))
p_2 <- p_2 + geom_boxplot(aes(fill = factor(Activity)), alpha = 0.7)
p_2 <- p_2 + theme(legend.position = ("none"),
                   panel.border = element_rect(linetype = "solid",
                                               colour = "black", fill = NA, size = 1),
                   axis.text.x = element_text(colour = "black", size = 15),
                   axis.text.y = element_text(colour = "black", size = 15),
                   plot.margin = unit(c(1, 1, 1, 1), "cm"),
                   axis.title.y = element_text(size = 20, face = "bold"),
                   axis.title.x = element_blank())
print(p_2)
```

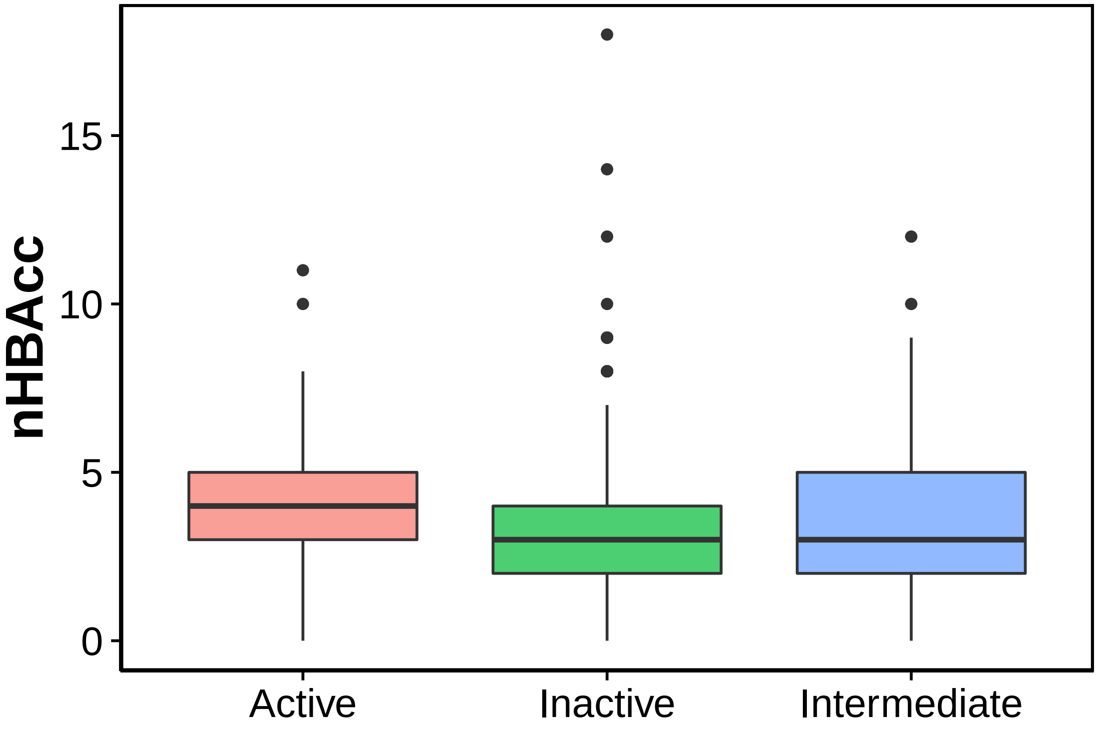

Boxplot of Lipinski's rule-of-five descriptors (nHBDon)

```
p_3 <- ggplot(data_plot, aes(factor(Activity), nHBDon))
p_3 <- p_3 + geom_boxplot(aes(fill = factor(Activity)), alpha = 0.7)
p_3 <- p_3 + theme(legend.position = ("none"),
                   panel.border = element_rect(linetype = "solid",
                                               colour = "black", fill = NA, size = 1),
                   axis.text.x = element_text(colour = "black", size = 15),
                   axis.text.y = element_text(colour = "black", size = 15),
                   plot.margin = unit(c(1, 1, 1, 1), "cm"),
                   axis.title.y = element_text(size = 20, face = "bold"),
                   axis.title.x = element_blank())
print(p_3)
```

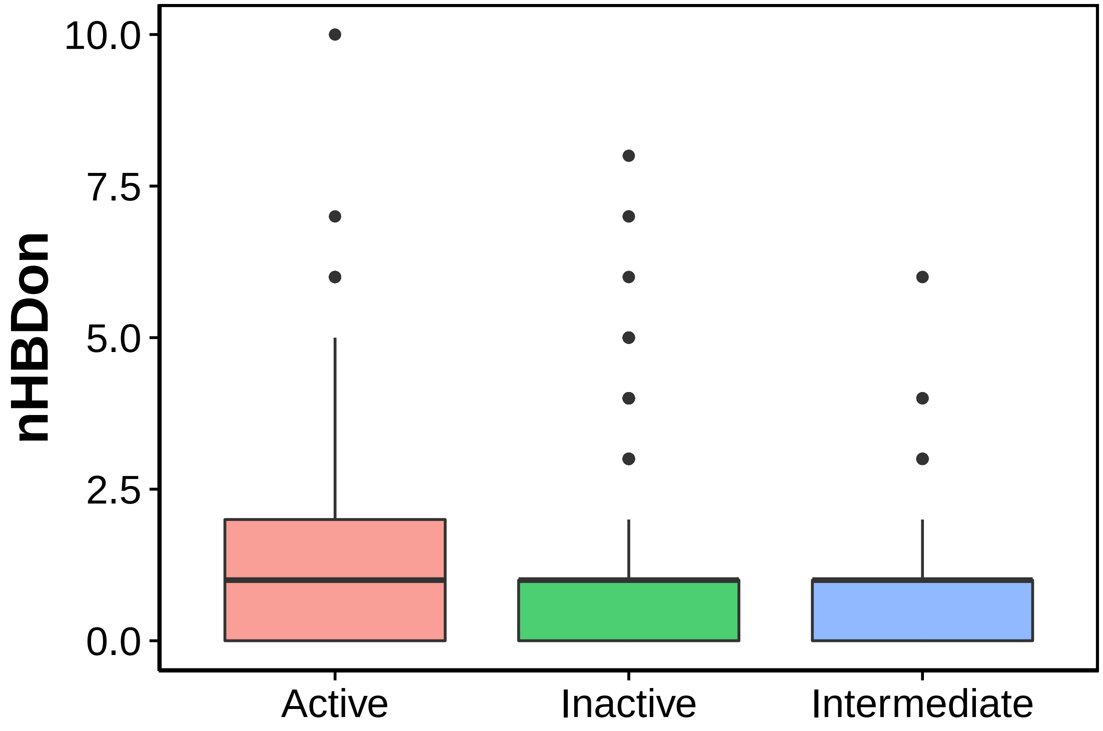

Boxplot of Lipinski's rule-of-five descriptors (MW)

```
p_4 <- ggplot(data_plot, aes(factor(Activity), MW))
p_4 <- p_4 + geom_boxplot(aes(fill = factor(Activity)), alpha = 0.7)
p_4 <- p_4 + theme(legend.position = ("none"),
                   panel.border = element_rect(linetype = "solid",
                                               colour = "black", fill = NA, size = 1),
                   axis.text.x = element_text(colour = "black", size = 15),
                   axis.text.y = element_text(colour = "black", size = 15),
                   plot.margin = unit(c(1, 1, 1, 0.4), "cm"),
                   axis.title.y = element_text(size = 20, face = "bold"),
                   axis.title.x = element_blank())

print(p_4)
```

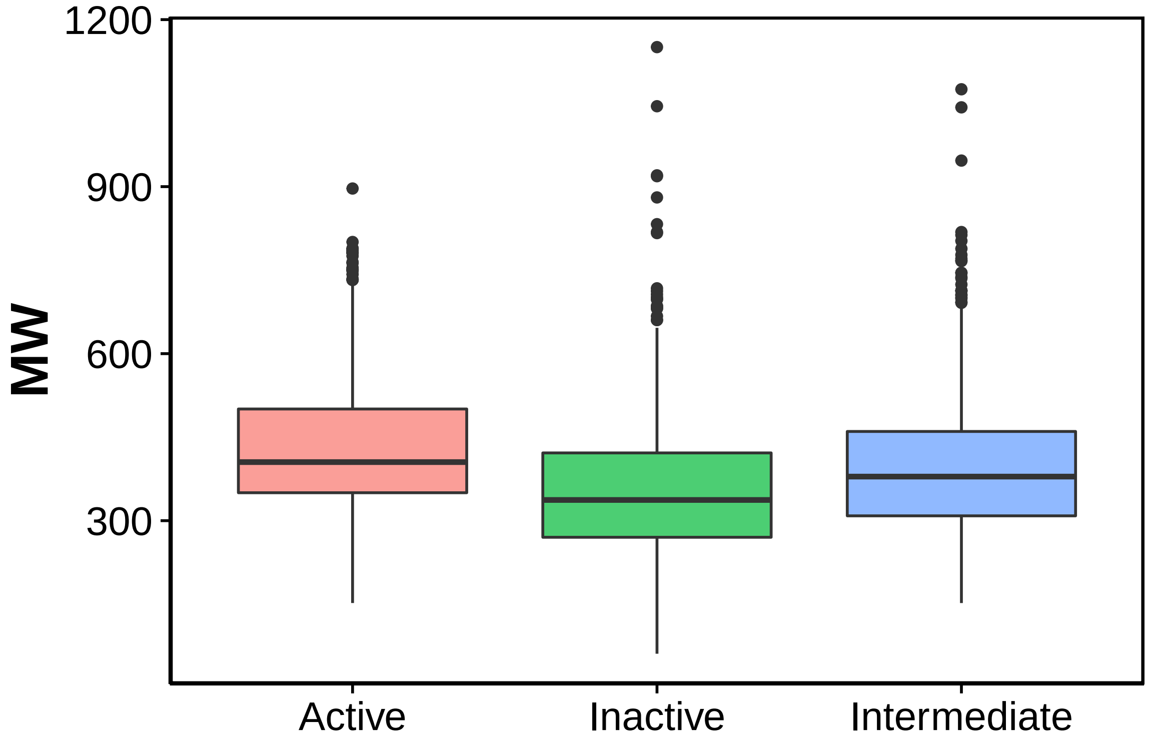

Arranging boxplot of Lipinski's rule-of-file descriptors (ALogP, nHBAcc, nHBDon, MW)

```
library(cowplot)
plot_grid(p_1, p_2, p_3, p_4)
```

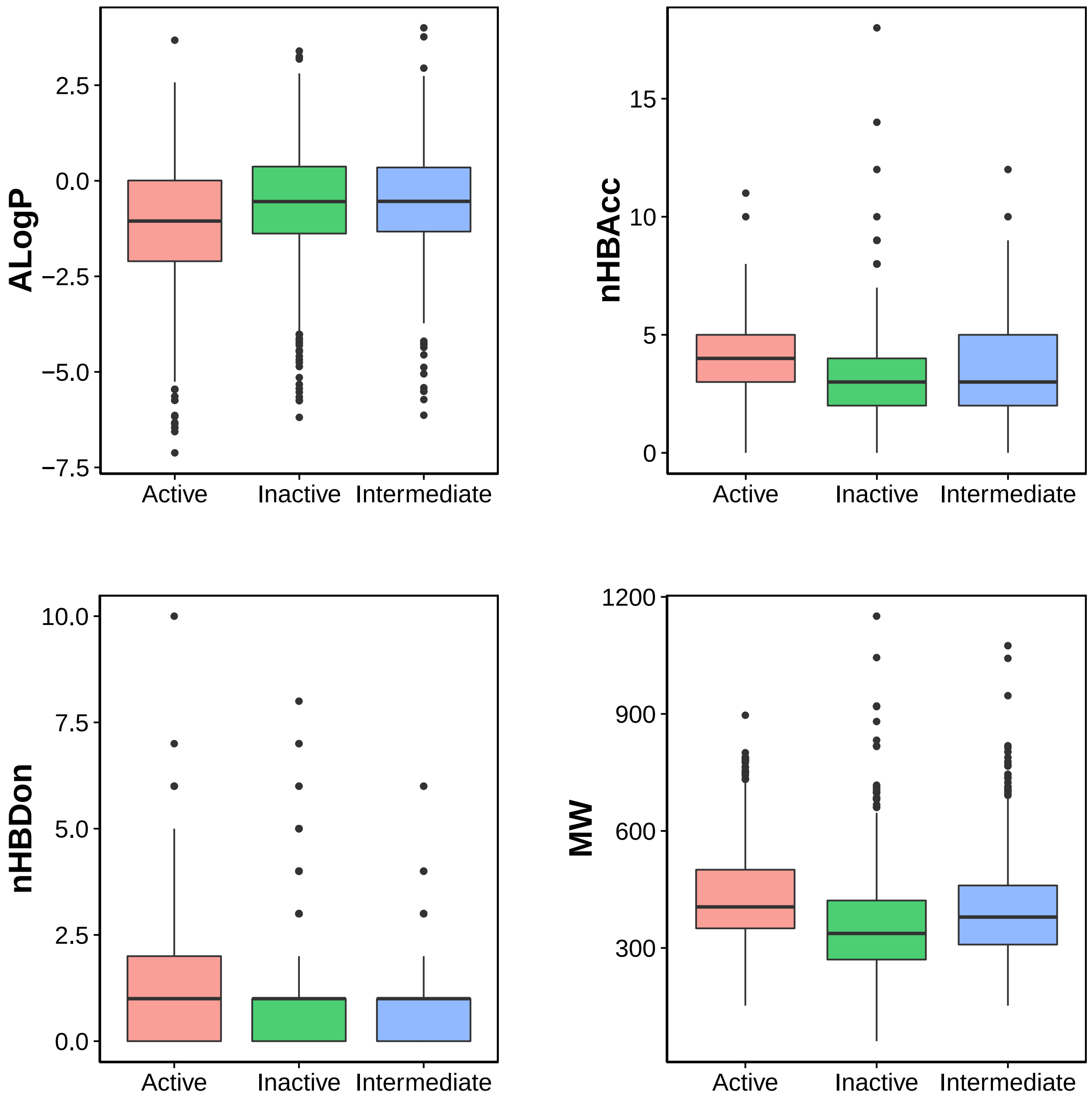

Supplement: Data S2 [file peerj-04-2322-s003.zip › R mark down/Chemical_Space.pdf]
