# Supplementary material for: Probing the origins of human acetylcholinesterase inhibition via QSAR modeling and molecular docking
Source: PeerJ. 2016 Aug 9;4:e2322. doi: 10.7717/peerj.2322 (PMC4991866; doi:10.7717/peerj.2322)
Supplement: Data S2 [file peerj-04-2322-s003.zip › R mark down/Feature_Importance.pdf]

Mechanistic interpretation of feature importance

Saw Simeon, Nuttapat Anuwongcharoen, Watshara Shoombuatong, Aijaz Ahmad Malik, Virapong Prachayasittikul, Jarl E. S. Wikberg and Chanin Nantasenamat

June 9, 2016

Feature importance analysis from Random forest using Gini index (i.e., variance of the responses).

Creating a R function to perform feature importance

```
randomForest_feature_importance <- function(x) {
  library(doSNOW)
  library(foreach)
  library(parallel)
  cl <- makeCluster(8)
  registerDoSNOW(cl)

  results <- list(100)
  results <- foreach (i = 1:100) %dopar% {
    x <- na.omit(x)
    set.seed(i)
    para <- dplyr::sample_n(x, size = 2570, replace = TRUE)
    set.seed(i)
    in_train_para <- sample(nrow(para),
                            size = as.integer(nrow(para) * 0.8),
                            replace = FALSE)
    Train <- para[in_train_para, ]
    Test <- para[-in_train_para, ]
    rm(in_train_para)
    rm(Test)
    set.seed(i)
    model <- ranger::ranger(pIC50~., data = Train, importance = 'impurity',
                                  write.forest = TRUE, save.memory = TRUE)
    rm(Train)
    importance <- model$variable.importance
    results[[i]] <- importance
  }
  return(results)
  stopCluster(cl)
}
```

Generating the plot of feature importance

```
input <- readRDS(file = "data.Rds")
SubStructure_fingerPrintCount <- input$Substructure_fingerPrintCount
set.seed(10)
results_feature_importance_RF <- randomForest_feature_importance(SubStructure_fingerPrintCount)
data1 <- data.frame(results_feature_importance_RF)
data1 <- cbind(features = rownames(data1), data1)
library(reshape2)
data_melt <- suppressWarnings(melt(data1, id.vars = "features"))
#data_melt <- melt(data1, id.vars = "features")
```

```r
data_melt$features <- factor(data_melt$features)
library(ggplot2)
set.seed(1)
plot_feature <- ggplot(data_melt, aes(x = reorder(features, value, FUN = median), y = value)) +
  geom_boxplot(fill = "#F8766D", colour = "black", alpha = 0.5) +
  theme_bw() + xlab("") + ylab("Gini Index") + coord_flip() + theme(
    axis.text.y = element_text(size = 20, colour = "black"),
    axis.text.x = element_text(size = 20, colour = "black"),
    #axis.title.x = element_blank(),
    plot.margin = unit(c(1, 1, 1, 1), "cm"),
    panel.border = element_rect(linetype = "solid", colour = "black", fill = NA, size = 1),
    axis.title = element_text(size = 25, face = "bold", colour = "black")
  )

plot_feature
```

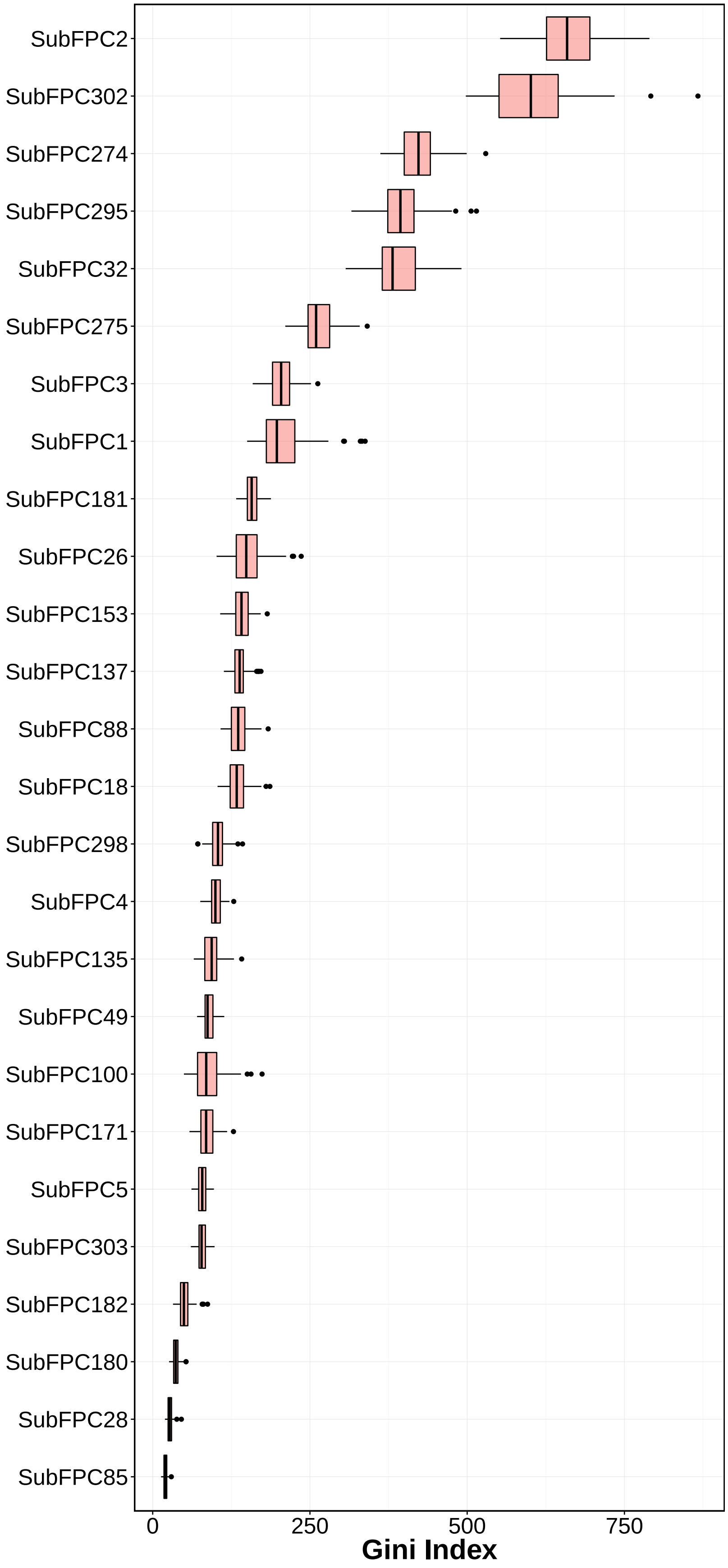
SubFPC2
SubFPC302
SubFPC274
SubFPC295
SubFPC32
SubFPC275
SubFPC3
SubFPC1
SubFPC181
SubFPC26
SubFPC153
SubFPC137
SubFPC88
SubFPC18
SubFPC298
SubFPC4
SubFPC135
SubFPC49
SubFPC100
SubFPC171
SubFPC5
SubFPC303
SubFPC182
SubFPC180
SubFPC28
SubFPC85
0
250
500
750
Gini Index
