# Supplementary material for: Probing the origins of human acetylcholinesterase inhibition via QSAR modeling and molecular docking
Source: PeerJ. 2016 Aug 9;4:e2322. doi: 10.7717/peerj.2322 (PMC4991866; doi:10.7717/peerj.2322)
Supplement: Data S2 [file peerj-04-2322-s003.zip › R mark down/Importing_Data.pdf]

Reading 12 Fingerprint from Padel Descriptor Calculator

Saw Simeon, Nuttapat Anuwongcharoen, Watshara Shoombuatong, Aijaz Ahmad Malik, Virapong Prachayasittikul, Jarl E. S. Wikberg and Chanin Nantasenamat

June 8, 2016

Function for Correlation Cut of at 0.7

```
### Regression Acetylachlineesterase
read_file <- function(x){
  library(caret)
  library(data.table)
  data <- fread(x)
  IC50_nm <- data$IC50
  IC50 <- as.numeric(IC50_nm)*10^-9
  pIC50 <- -log10(IC50)
  data <- as.data.frame(data)
  descriptors <- data[, 2:ncol(data)]
  set.seed(1)
  yes <- descriptors[, -nearZeroVar(descriptors)]
  raw <- cor(yes)
  raw_2 <- raw[1: ncol(raw), 1:ncol(raw)]
  high <- findCorrelation(raw_2, cutoff = 0.7)
  filtered_descriptors <- yes[, -high]
  filtered_data <- cbind(pIC50, filtered_descriptors)
  return(filtered_data)
}
```

Reading each data frame and printing out of the dimension of each data Frame

```
AtomPairs2D_fingerPrintCount <- read_file("data/2D_Atom_Paris_Count.csv")
```

```
## Warning: package 'caret' was built under R version 3.2.4

## Loading required package: lattice

## Loading required package: ggplot2

## Warning: package 'ggplot2' was built under R version 3.2.4
```

```
AtomPairs2D_fingerPrinter <- read_file("data/2D_Atom_Pairs.csv")
Substructure_fingerPrintCount <- read_file("data/Substructure_Count.csv")
Substructure_fingerPrinter <- read_file("data/Substructure.csv")
Extended_finterPrinter <- read_file("data/CDK_Extended.csv")
FingerPrinter <- read_file("data/CDK.csv")
Estate_FingerPrinter <- read_file("data/E_State.csv")
GraphOnly_FingerPrinter <- read_file("data/CDK_Graph_Only.csv")
KlekotaRoth_FingerprintCount <- read_file("data/Klekota_Roth_Count.csv")
KlekotaRoth_FingerPrinter <- read_file("data/Klekota_Roth.csv")
MACCS_FingerPrinter <- read_file("data/MACCS.csv")
```

```r
Pubchem_FingerPrinter <- read_file("data/PubChem.csv")


input <- list(AtomPairs2D_fingerPrintCount=AtomPairs2D_fingerPrintCount,
              AtomPairs2D_fingerPrinter = AtomPairs2D_fingerPrinter,
              Substructure_fingerPrintCount = Substructure_fingerPrintCount,
              Substructure_fingerPrinter = Substructure_fingerPrinter,
              Extended_finterPrinter = Extended_finterPrinter,
              FingerPrinter = FingerPrinter,
              Estate_FingerPrinter = Estate_FingerPrinter,
              GraphOnly_FingerPrinter = GraphOnly_FingerPrinter,
              KlekotaRoth_FingerprintCount = KlekotaRoth_FingerprintCount,
              KlekotaRoth_FingerPrinter = KlekotaRoth_FingerPrinter,
              MACCS_FingerPrinter = MACCS_FingerPrinter,
              Pubchem_FingerPrinter = Pubchem_FingerPrinter)

print(lapply(input, function(x) dim(x)))
```

```
## $AtomPairs2D_fingerPrintCount
## [1] 2570   39
##
## $AtomPairs2D_fingerPrinter
## [1] 2570   43
##
## $Substructure_fingerPrintCount
## [1] 2570   27
##
## $Substructure_fingerPrinter
## [1] 2570   31
##
## $Extended_finterPrinter
## [1] 2570  949
##
## $FingerPrinter
## [1] 2570  961
##
## $Estate_FingerPrinter
## [1] 2570   22
##
## $GraphOnly_FingerPrinter
## [1] 2570  199
##
## $KlekotaRoth_FingerprintCount
## [1] 2570   73
##
## $KlekotaRoth_FingerPrinter
## [1] 2570  112
##
## $MACCS_FingerPrinter
## [1] 2570   78
##
## $Pubchem_FingerPrinter
## [1] 2570  104
```
