# Supplementary material for: Probing the origins of human acetylcholinesterase inhibition via QSAR modeling and molecular docking
Source: PeerJ. 2016 Aug 9;4:e2322. doi: 10.7717/peerj.2322 (PMC4991866; doi:10.7717/peerj.2322)

Plot Interval Graph (Prediction Vs Observed inhibitory activities)

Saw Simeon, Nuttapat Anuwongcharoen, Watshara Shoombuatong, Aijaz Ahmad Malik, Virapong Prachayasittikul, Jarl E. S. Wikberg and Chanin Nantasenamat

June 16, 2016

Create a predicted and IC50 plot

```
file <- function(x) {
  library(randomForest)
  library(caret)
  library(ranger)
  library(cowplot)
  set.seed(10)
  para <- dplyr::sample_n(x, size = 2570, replace = TRUE)
  set.seed(3)
  in_train_para <- sample(nrow(para),
                          size = as.integer(nrow(para) * 0.8),
                          replace = FALSE)
  set.seed(4)
  Train <- para[in_train_para, ]
  Test  <- para[-in_train_para, ]

  model <- ranger::ranger(pIC50~., data = Train, write.forest = TRUE, save.memory = TRUE)
  #actual <- train$Activity
  prediction <- predict(model, Train)
  prediction_Internal <- prediction$predictions
  value <- data.frame(obs = Train$pIC50, pred = prediction_Internal)
  labeling <- c("obs", "pred")
  colnames(value) <- labeling
  value$Label <- c("Internal")
  prediction_External <- predict(model, Test)
  prediction_External <- prediction_External$predictions
  value_external <- data.frame(obs = Test$pIC50, pred = prediction_External)
  colnames(value_external) <- labeling
  value_external$Label <- c("External")
  results <- rbind(value, value_external)
  return(results)
}


get_interval <- function(x) {
  file <- file(x)
  x <- file[, 1]
  y <- file[, 2]
  label <- file[3]
  fit <- lm(y~x)
  pred.int <- predict(fit, interval = "prediction")
```

```
  pred.lower = pred.int[,2]
  pred.upper = pred.int[,3]
  df <- cbind(x, y, label, pred.lower, pred.upper)
  return(df)
}

plot_graph_interval <- function(x) {
  library(ggplot2)
  ok <- get_interval(x)
  good <- ggplot(ok, aes(x = x)) +
    geom_point(size = 7, colour = "black", pch = 21, alpha= 0.4,
               aes(y = y, fill = factor(Label))) +
    geom_line(aes(y = pred.lower), size = 1.5, colour = "grey", linetype = 2) +
    geom_line(aes(y = pred.upper), size = 1.5, colour = "grey", linetype = 2) +
    xlab(expression(paste('Predicted ', pIC[50]))) + ylab(expression(paste('Experimental ', pIC[50])))
    #geom_abline(intercept = ok$pred.lower[[1]], linetype = 2, size = 1.5, colour = "grey") +
    #geom_abline(intercept = ok$pred.upper[[1]], linetype = 2, size = 1.5, colour = "grey") +
    theme(
      panel.border = element_rect(linetype = "solid", colour = "black",
                                  fill = NA, size = 1),

    axis.text.y = element_text(size = 20, colour = "black"),
    axis.text.x = element_text(size = 20, colour = "black"),
    axis.title.x = element_text(size = 30, color = "black", face = "bold"),
    axis.title.y = element_text(size = 30, color = "black", face = "bold"),

      legend.position = ("none")) +
    coord_cartesian(ylim = c(-6, 12), xlim = c(-6, 12))
  return(good)

}
```

CDK fingerprint

```
input <- readRDS("data.Rds")
df <- input$FingerPrinter
plot_graph_interval(df)
```

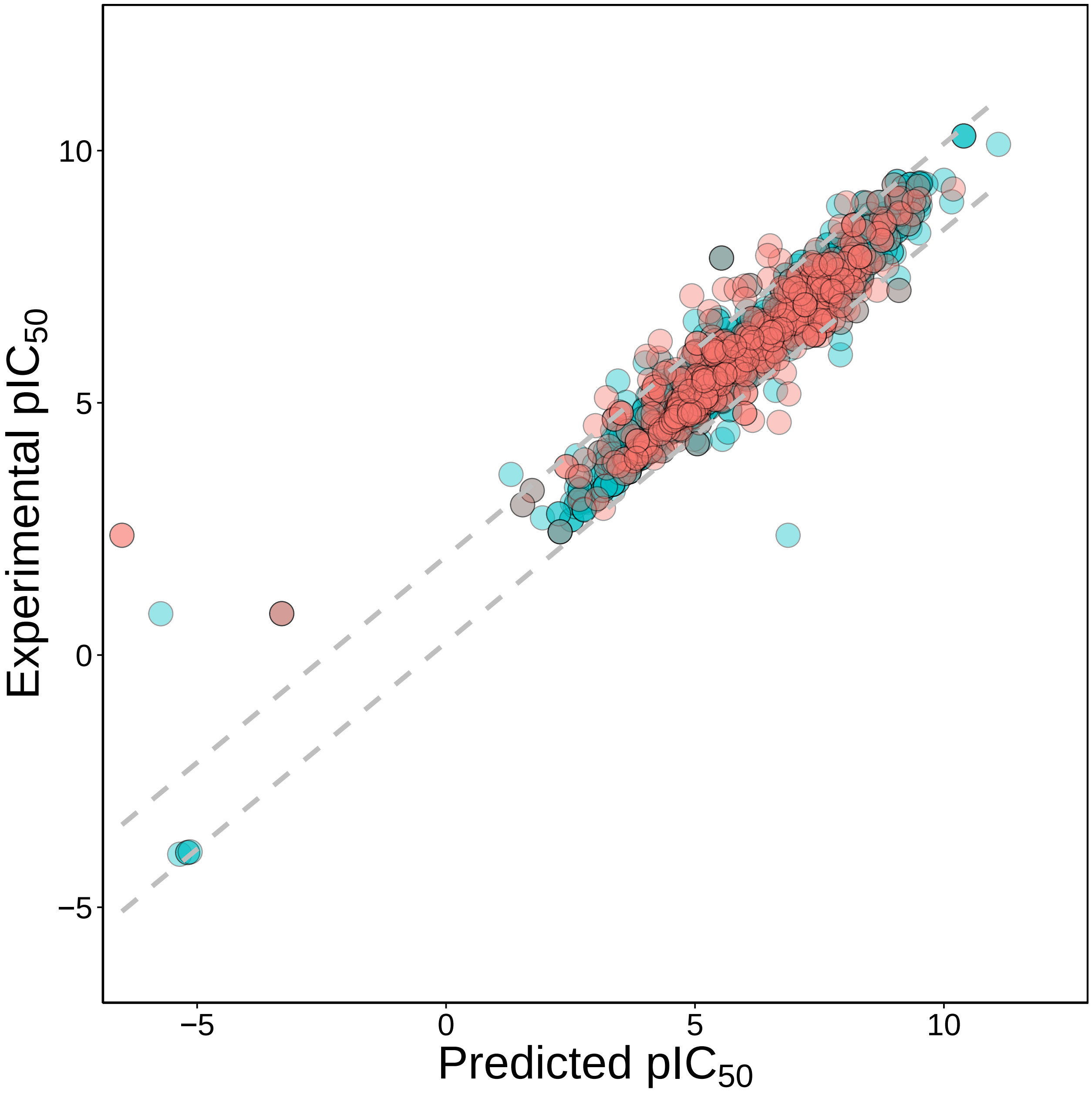

CDK extended fingerprint

```r
input <- readRDS("data.Rds")
df <- input$Extended_finterPrinter
plot_graph_interval(df)
```

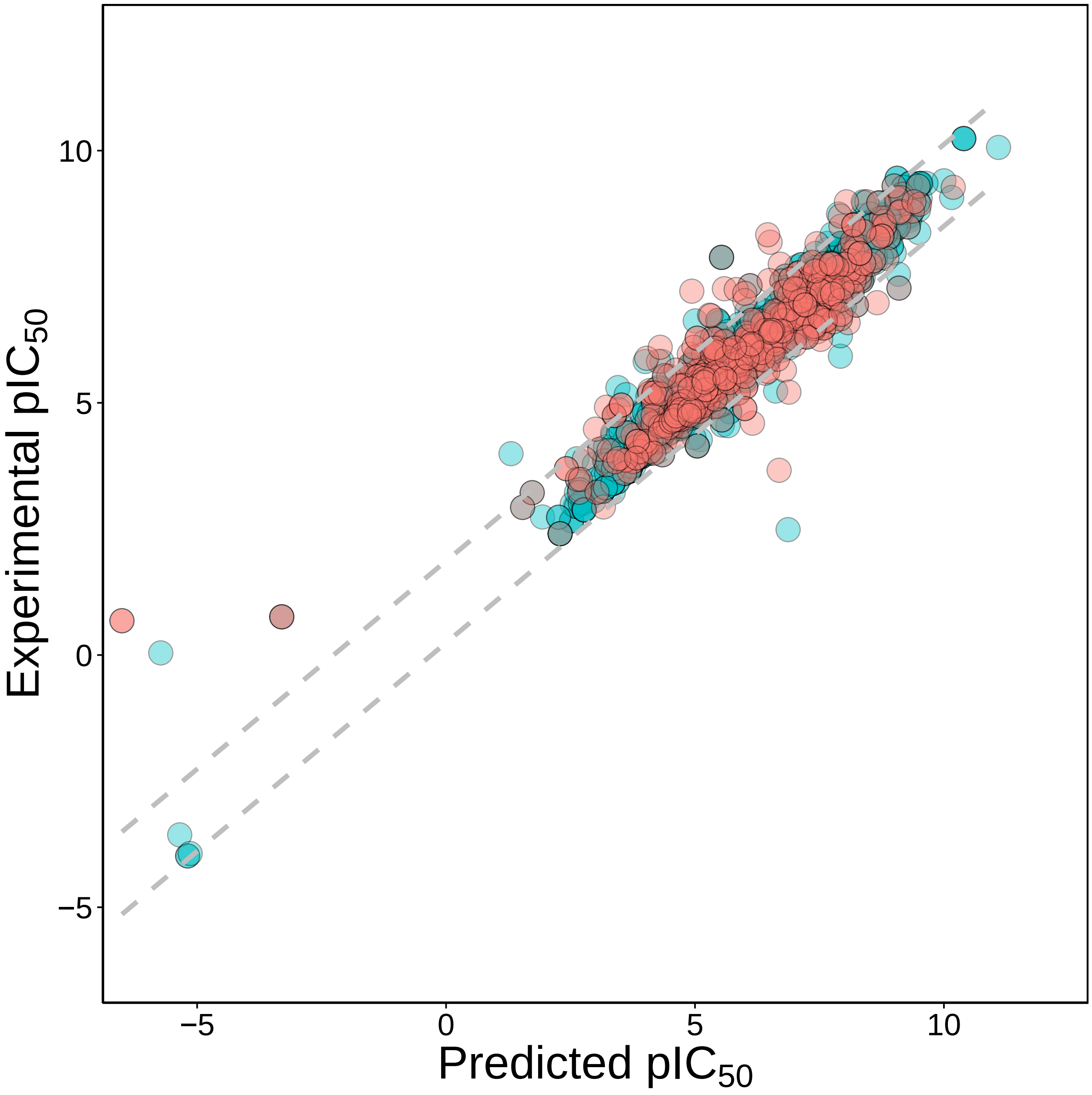

CDK graph only fingerprint

```
input <- readRDS("data.Rds")
df <- input$GraphOnly_FingerPrinter
plot_graph_interval(df)
```

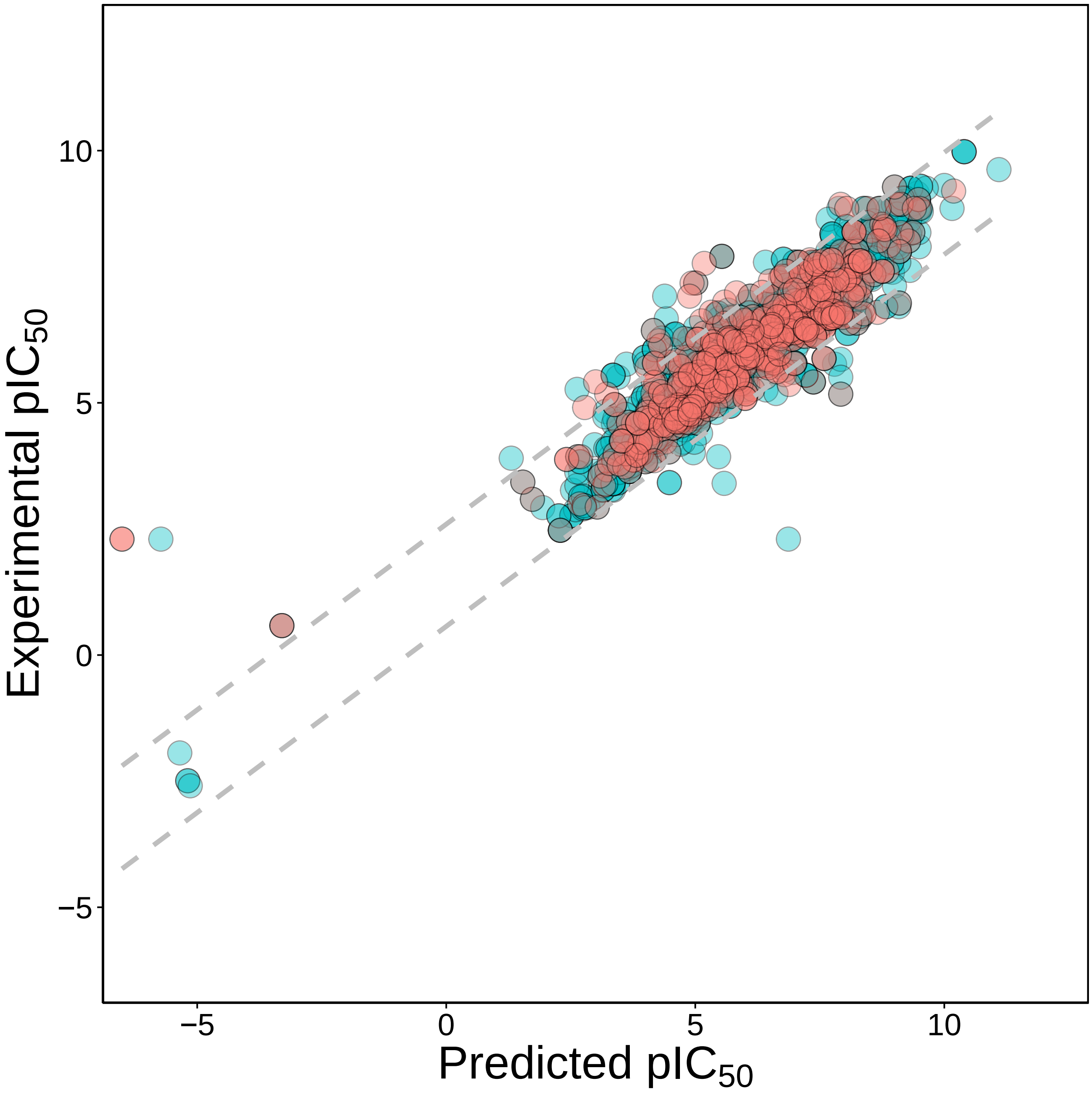

E-state fingerprint

```
input <- readRDS("data.Rds")
df <- input$Extended_finterPrinter
plot_graph_interval(df)
```

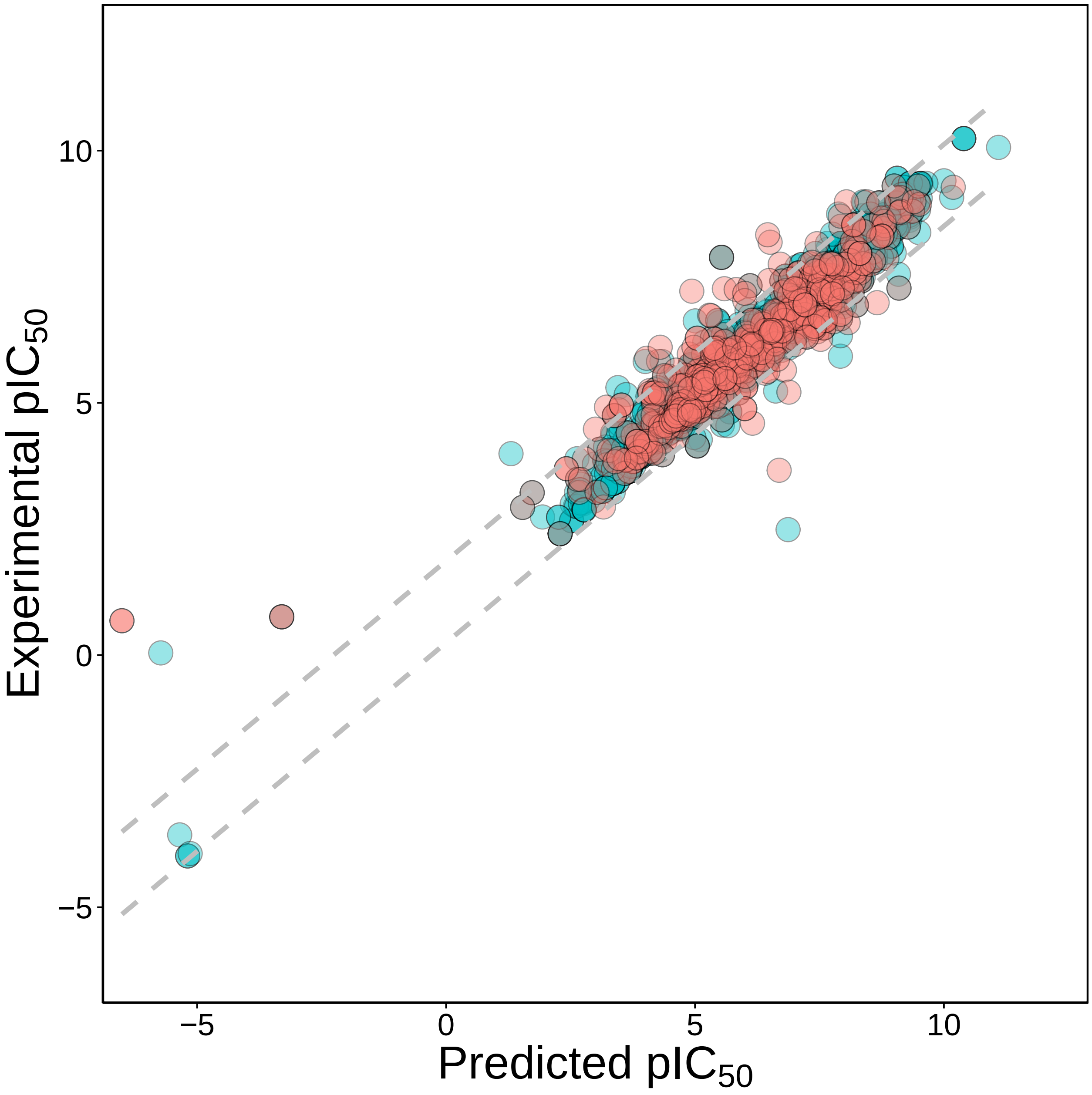

MACCS fingerprint

```
input <- readRDS("data.Rds")
df <- input$MACCS_FingerPrinter
plot_graph_interval(df)
```

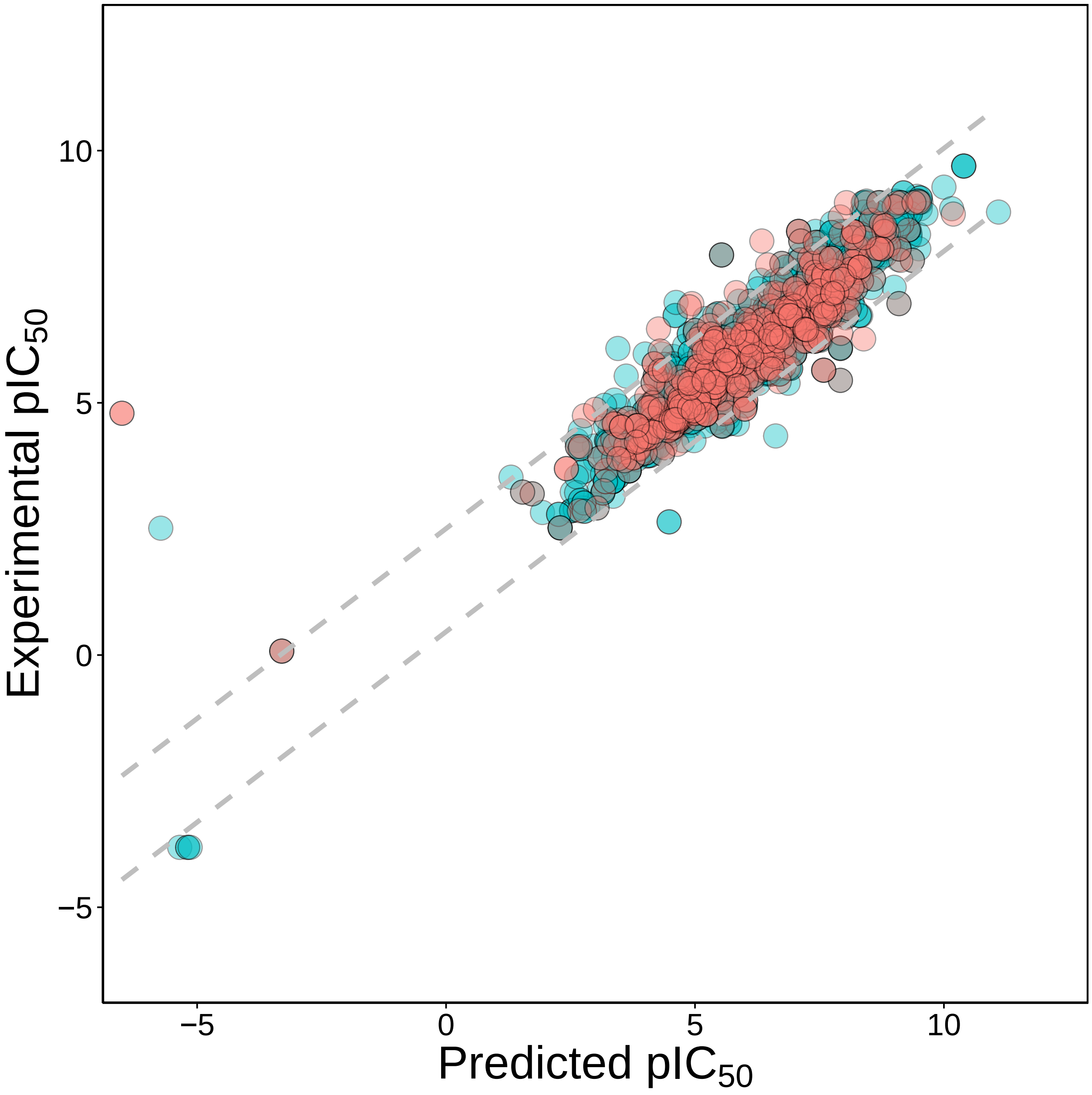

PubChem fingerprint

```r
input <- readRDS("data.Rds")
df <- input$Pubchem_FingerPrinter
plot_graph_interval(df)
```

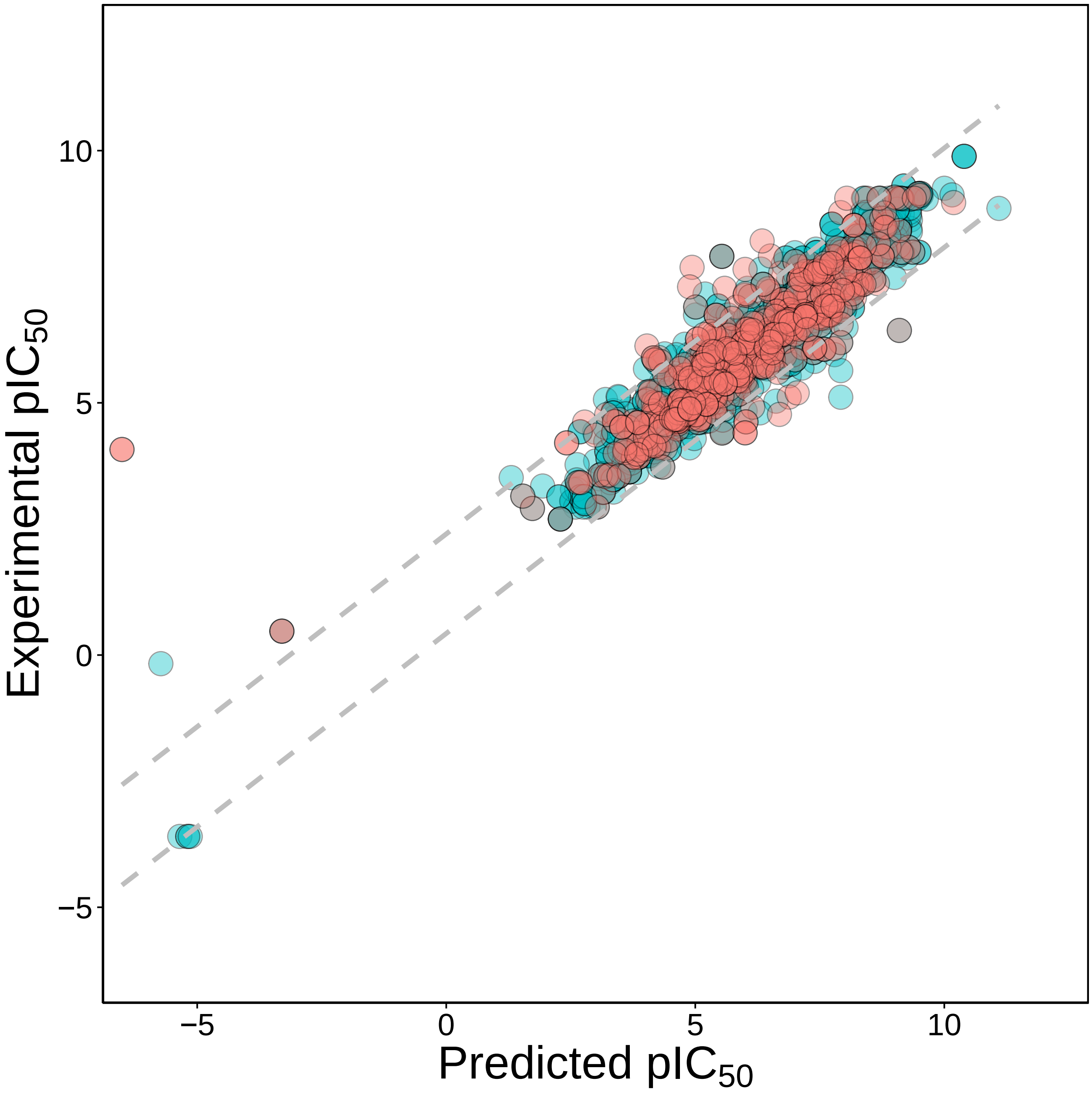

Substructure fingerprint

```
input <- readRDS("data.Rds")
df <- input$Substructure_fingerPrinter
plot_graph_interval(df)
```

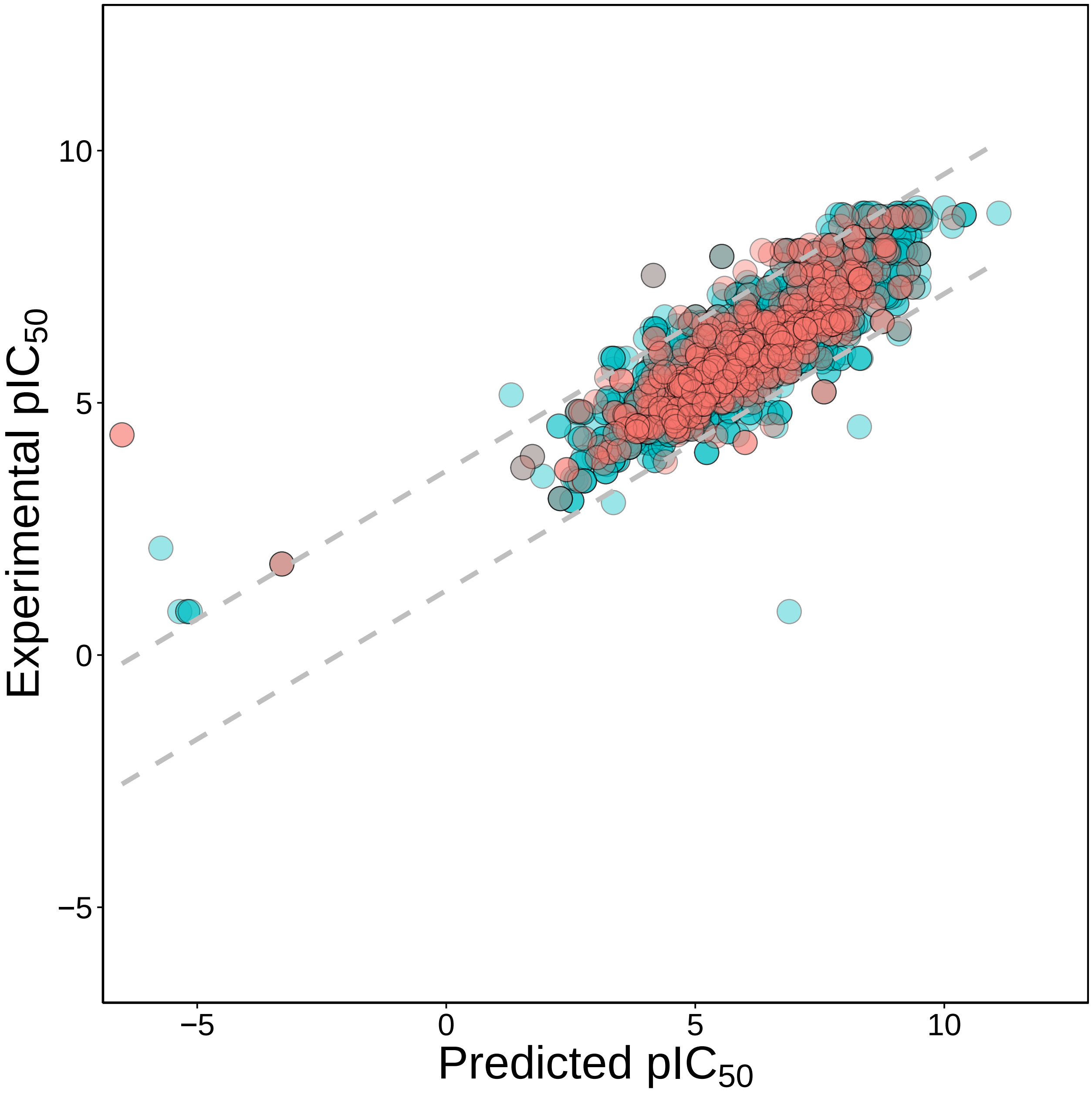

Substructure count

```
input <- readRDS("data.Rds")
df <- input$Substructure_fingerPrintCount
plot_graph_interval(df)
```

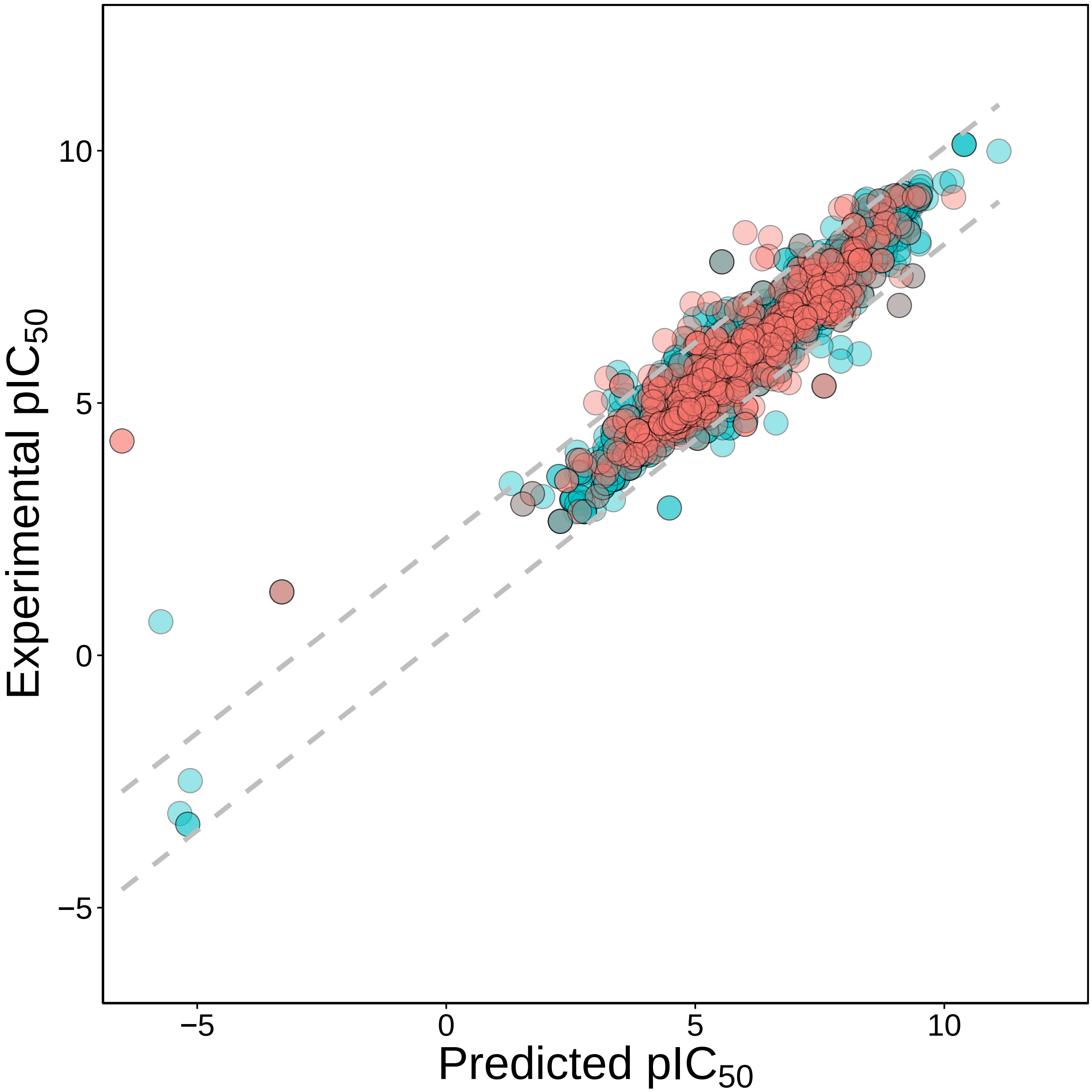

Klekota-Roth fingerprint

```
input <- readRDS("data.Rds")
df <- input$KlekotaRoth_FingerPrinter
plot_graph_interval(df)
```

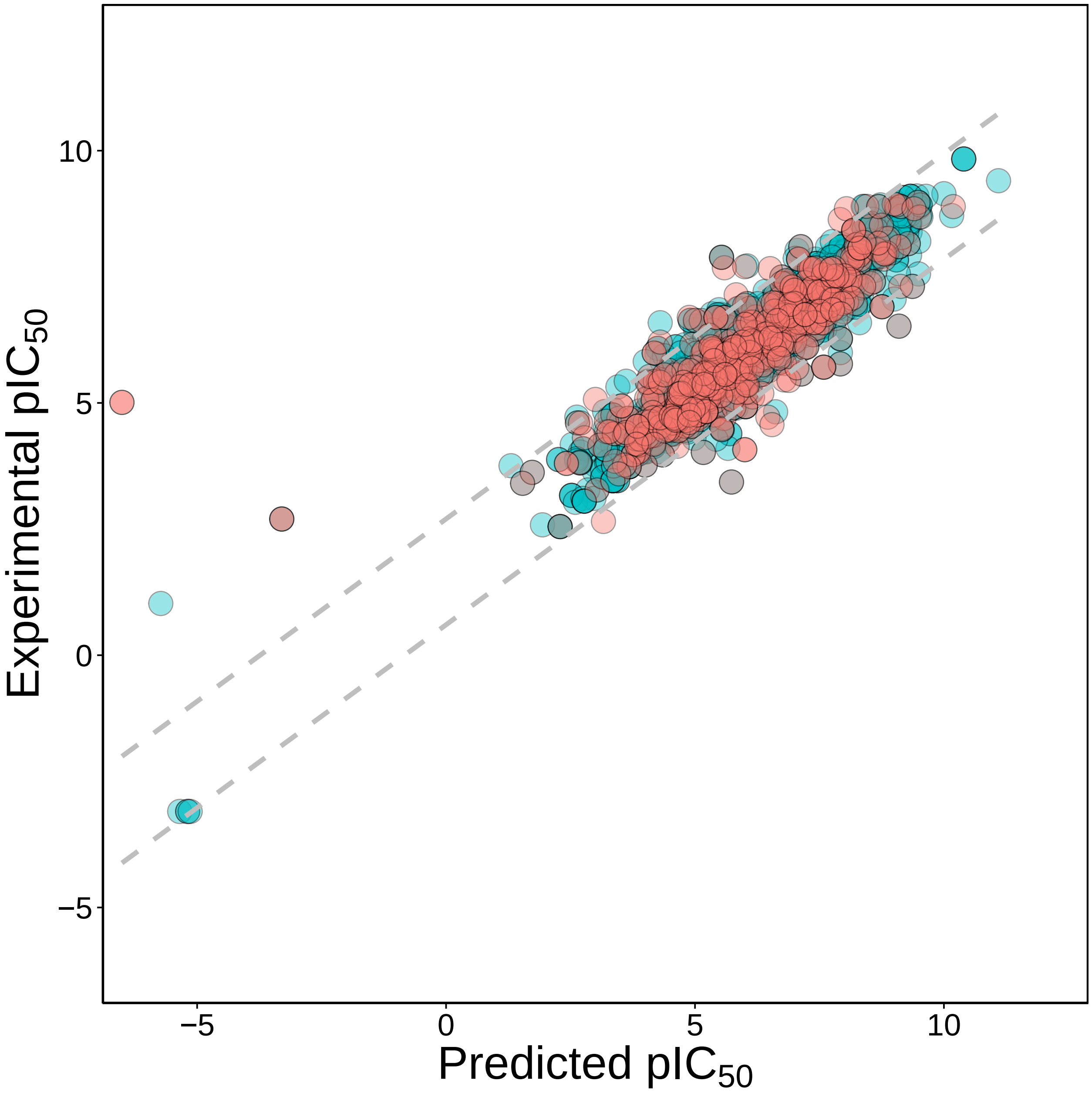

Klekota-Roth count

```
input <- readRDS("data.Rds")
df <- input$KlekotaRoth_FingerprintCount
plot_graph_interval(df)
```

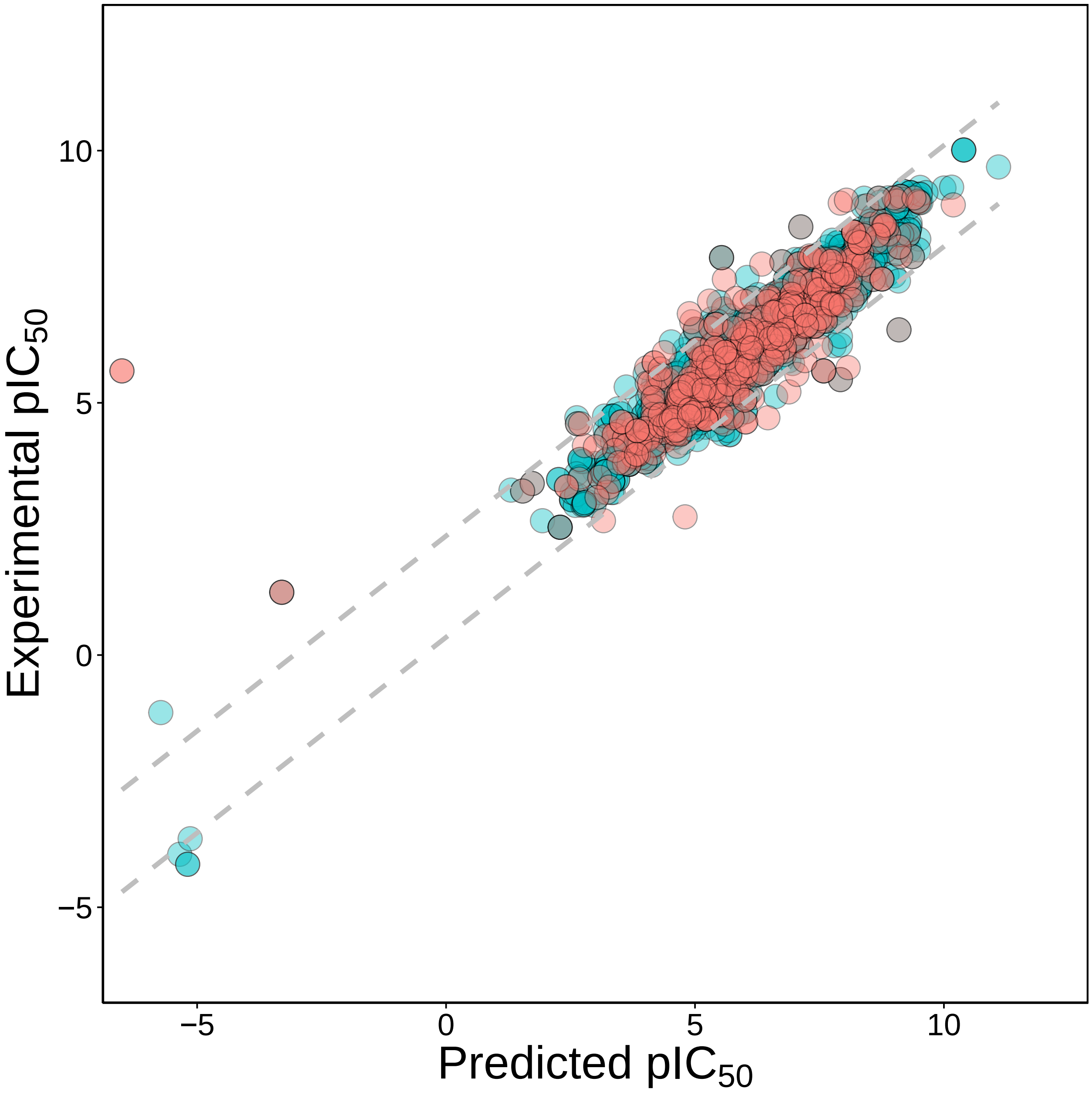

2D atom pairs

```
input <- readRDS("data.Rds")
df <- input$AtomPairs2D_fingerPrinter
plot_graph_interval(df)
```

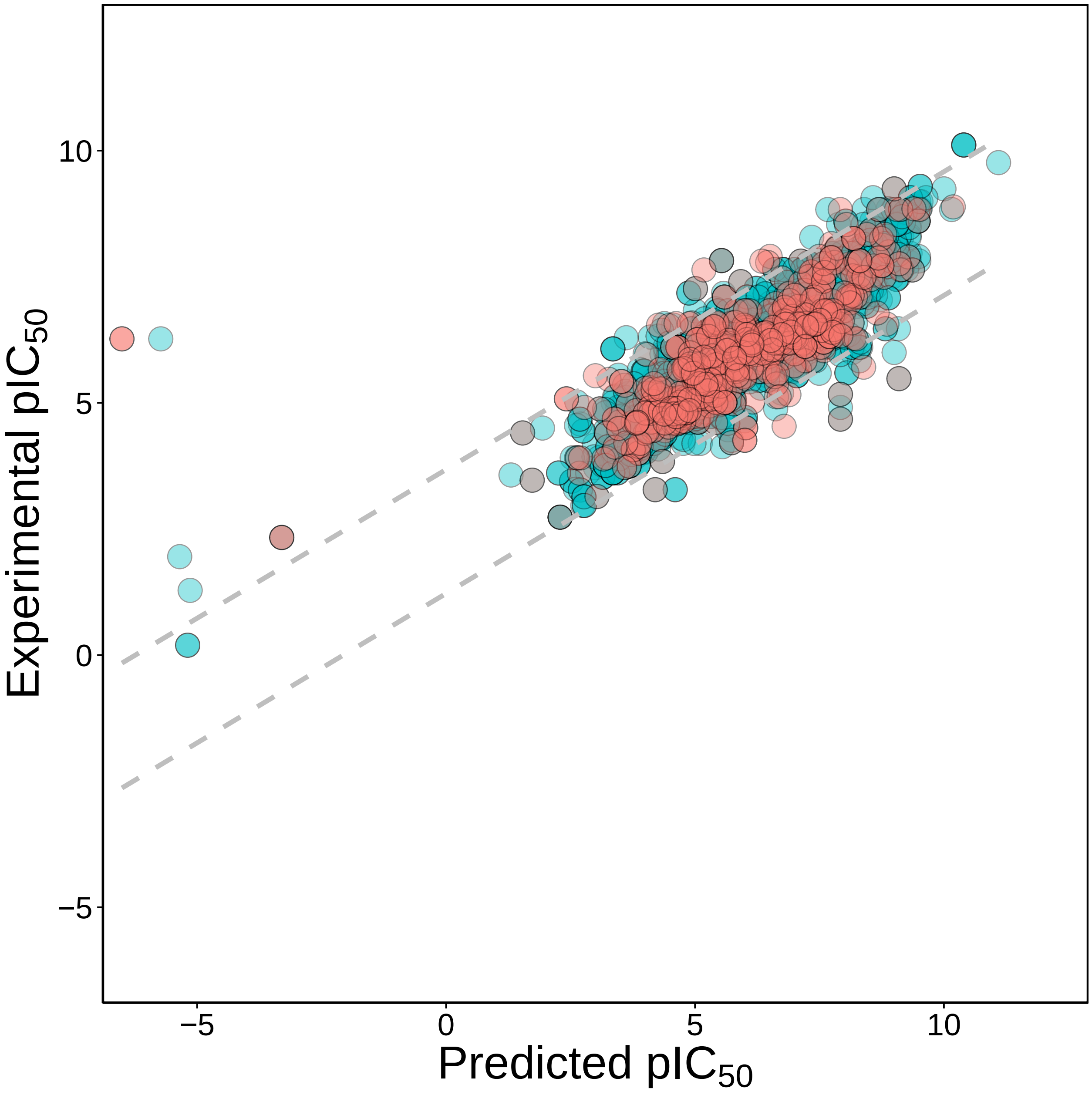

2D atom pairs count

```
input <- readRDS("data.Rds")
df <- input$AtomPairs2D_fingerPrintCount
plot_graph_interval(df)
```

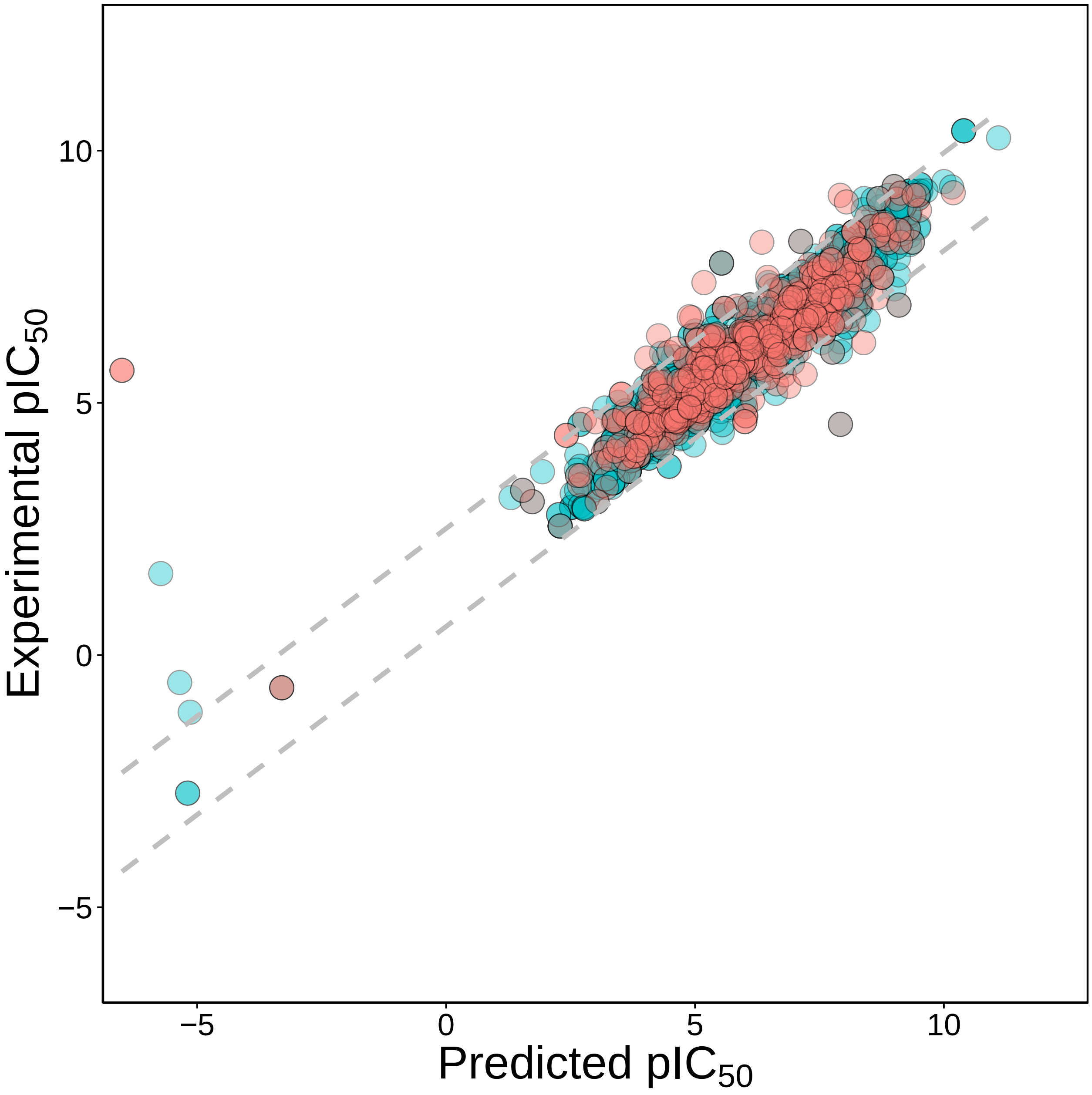

Supplement: Data S2 [file peerj-04-2322-s003.zip › R mark down/Interval_Plot.pdf]
