# Supplementary material for: Probing the origins of human acetylcholinesterase inhibition via QSAR modeling and molecular docking
Source: PeerJ. 2016 Aug 9;4:e2322. doi: 10.7717/peerj.2322 (PMC4991866; doi:10.7717/peerj.2322)
Supplement: Data S2 [file peerj-04-2322-s003.zip › R mark down/Machine_Learning.pdf]

# Random Forest Regression

*Saw Simeon, Nuttapat Anuwongcharoen, Watshara Shoombuatong, Aijaz Ahmad Malik, Virapong Prachayasittikul, Jarl E. S. Wikberg and Chanin Nantasenamat*

*June 8, 2016*

Modeling with Random forest

```
randomForest_training <- function(x) {
    library(randomForest)

    library(parallel)
    library(doSNOW)
    cl <- makeCluster(8)
    registerDoSNOW(cl)

    R2 <- function(y, equation, ...) {
        1 - (sum((y - predict(equation))^2)/sum((y - mean(y))^2))
    }
    rm2 <- function(y, x, ...) {
        if ((R2(y, (lm(y ~ x)))) > R2(y, (lm(y ~ -1 + x)))) {
            return(R2(y, (lm(y ~ x))) * (1 - (sqrt(R2(y, (lm(y ~ x))) - R2(y,
                (lm(y ~ -1 + x)))))))
        } else {
            return(R2(y, (lm(y ~ x))))
        }
    }
    rm2.reverse <- function(y, x, ...) {
        return(R2(x, (lm(x ~ y))) * (1 - (sqrt(R2(x, (lm(x ~ y))) - R2(x, (lm(x ~
            -1 + y)))))))
    }
    average.rm2 <- function(y, x, ...) {
        if ((R2(y, (lm(y ~ x)))) > R2(y, (lm(y ~ -1 + x)))) {
            return(((R2(y, (lm(y ~ x))) * (1 - (sqrt(R2(y, (lm(y ~ x))) - R2(y,
                (lm(y ~ -1 + x)))))) + R2(x, (lm(x ~ y))) * (1 - (sqrt(R2(x,
                (lm(x ~ y))) - R2(x, (lm(x ~ -1 + y))))))))/2)
        } else {
            return(((R2(y, (lm(y ~ x)))) + (R2(x, (lm(x ~ y))) * (1 - (sqrt(R2(x,
                (lm(x ~ y))) - R2(x, (lm(x ~ -1 + y))))))))/2)
        }
    }
    delta.rm2 <- function(y, x, ...) {
        if ((R2(y, (lm(y ~ x)))) > R2(y, (lm(y ~ -1 + x)))) {
            return(abs((R2(y, (lm(y ~ x))) * (1 - (sqrt(R2(y, (lm(y ~ x))) -
               R2(y, (lm(y ~ -1 + x)))))) - R2(x, (lm(x ~ y))) * (1 - (sqrt(R2(x,
                (lm(x ~ y))) - R2(x, (lm(x ~ -1 + y)))))))))
        } else {
            return(abs((R2(y, (lm(y ~ x)))) - (R2(x, (lm(x ~ y))) * (1 - (sqrt(R2(x,
                (lm(x ~ y))) - R2(x, (lm(x ~ -1 + y)))))))))
        }
    }
```

```
    results <- list(100)
    results <- foreach(i = 1:100) %dopar% {



        x <- na.omit(x)
        para <- dplyr::sample_n(x, size = 2570, replace = TRUE)
        in_train_para <- sample(nrow(para), size = as.integer(nrow(para) * 0.8),
            replace = FALSE)
        Train <- para[in_train_para, ]
        Test <- para[-in_train_para, ]


        model_train <- ranger::ranger(pIC50 ~ ., data = Train, write.forest = TRUE,
            save.memory = TRUE)
        # actual <- train$Activity
        prediction <- predict(model_train, Train)
        prediction <- prediction$predictions

        # prediction <- predict(model, Train)
        value <- data.frame(obs = Train$pIC50, pred = prediction)
        rm(para)
        rm(Train)
        rm(Test)
        rm(model_train)
        rm(prediction)
        labeling <- c("obs", "pred")
        colnames(value) <- labeling
        result <- caret::defaultSummary(value)
        result_rm2 <- rm2(value$obs, value$pred)
        names(result_rm2) <- "rm2"
        results_reverse <- rm2.reverse(value$obs, value$pred)
        names(results_reverse) <- "reverse.rm2"
        result_average_rm2 <- average.rm2(value$obs, value$pred)
        names(result_average_rm2) <- "average.rm2"
        result_delta <- delta.rm2(value$obs, value$pred)
        names(result_delta) <- "delta.rm"

        results[[i]] <- c(result, result_rm2, results_reverse, result_average_rm2,
            result_delta)
    }
    return(results)
    stopCluster(cl)
}

mean_and_sd <- function(x) {
    c(round(rowMeans(x, na.rm = TRUE), digits = 2), round(genefilter::rowSds(x,
        na.rm = TRUE), digits = 2))
}

randomForest_train <- function(x) {
    ok <- randomForest_training(x)
    data <- data.frame(ok)
```

```r
    result <- mean_and_sd(data)
    df <- data.frame(result)
    R2_and_RMSE <- t(df)
    label <- c("RMSE_Mean", "Rsquared_Mean", "RM2_Mean", "Reverse_RM2_Mean",
        "Average_RM2_Mean", "Delta_RM2_Mean", "RMSE_SD", "Rsquared_SD", "RM2_SD",
        "Reverse_RM2_SD", "Average_RM2_SD", "Delta_RM2_SD")
    colnames(R2_and_RMSE) <- label
    return(R2_and_RMSE)
}

rf_cross_validation <- function(x) {
    library(randomForest)

    library(parallel)
    library(doSNOW)
    cl <- makeCluster(8)
    registerDoSNOW(cl)

    R2 <- function(y, equation, ...) {
        1 - (sum((y - predict(equation))^2)/sum((y - mean(y))^2))
    }
    rm2 <- function(y, x, ...) {
        if ((R2(y, (lm(y ~ x)))) > R2(y, (lm(y ~ -1 + x)))) {
            return(R2(y, (lm(y ~ x))) * (1 - (sqrt(R2(y, (lm(y ~ x))) - R2(y,
                (lm(y ~ -1 + x)))))))
        } else {
            return(R2(y, (lm(y ~ x))))
        }
    }
    rm2.reverse <- function(y, x, ...) {
        return(R2(x, (lm(x ~ y))) * (1 - (sqrt(R2(x, (lm(x ~ y))) - R2(x, (lm(x ~
            -1 + y)))))))
    }
    average.rm2 <- function(y, x, ...) {
        if ((R2(y, (lm(y ~ x)))) > R2(y, (lm(y ~ -1 + x)))) {
            return(((R2(y, (lm(y ~ x))) * (1 - (sqrt(R2(y, (lm(y ~ x))) - R2(y,
                (lm(y ~ -1 + x)))))) + R2(x, (lm(x ~ y))) * (1 - (sqrt(R2(x,
                (lm(x ~ y))) - R2(x, (lm(x ~ -1 + y))))))))/2)
        } else {
            return(((R2(y, (lm(y ~ x)))) + (R2(x, (lm(x ~ y))) * (1 - (sqrt(R2(x,
                (lm(x ~ y))) - R2(x, (lm(x ~ -1 + y))))))))/2)
        }
    }
    delta.rm2 <- function(y, x, ...) {
        if ((R2(y, (lm(y ~ x)))) > R2(y, (lm(y ~ -1 + x)))) {
            return(abs((R2(y, (lm(y ~ x))) * (1 - (sqrt(R2(y, (lm(y ~ x))) -
               R2(y, (lm(y ~ -1 + x)))))) - R2(x, (lm(x ~ y))) * (1 - (sqrt(R2(x,
                (lm(x ~ y))) - R2(x, (lm(x ~ -1 + y)))))))))
        } else {
            return(abs((R2(y, (lm(y ~ x)))) - (R2(x, (lm(x ~ y))) * (1 - (sqrt(R2(x,
                (lm(x ~ y))) - R2(x, (lm(x ~ -1 + y)))))))))
        }
    }
```

```
    results <- list(100)
    results <- foreach(i = 1:100) %dopar% {

        cool <- na.omit(x)
        para <- dplyr::sample_n(cool, size = 2570, replace = TRUE)
        in_train_para <- sample(nrow(para), size = as.integer(nrow(para) * 0.8),
            replace = FALSE)
        myData <- para[in_train_para, ]
        Test <- para[-in_train_para, ]
        rm(Test)
        k = 10
        index <- sample(1:k, nrow(myData), replace = TRUE)
        folds <- 1:k
        myRes <- data.frame()
        for (j in 1:k) {
            training <- subset(myData, index %in% folds[-j])
            testing <- subset(myData, index %in% c(j))
            model_train <- ranger::ranger(pIC50 ~ ., data = training, write.forest = TRUE,
                save.memory = TRUE)
            prediction <- predict(model_train, testing)
            prediction <- prediction$predictions

            ok <- data.frame(obs = testing$pIC50, pred = prediction)
            value <- rbind(myRes, ok)
        }
        rm(myData)
        rm(para)
        rm(in_trian_para)
        rm(training)
        rm(testing)
        rm(prediction)
        labeling <- c("obs", "pred")
        colnames(value) <- labeling
        result <- caret::defaultSummary(value)
        result_rm2 <- rm2(value$obs, value$pred)
        names(result_rm2) <- "rm2"
        results_reverse <- rm2.reverse(value$obs, value$pred)
        names(results_reverse) <- "reverse.rm2"
        result_average_rm2 <- average.rm2(value$obs, value$pred)
        names(result_average_rm2) <- "average.rm2"
        result_delta <- delta.rm2(value$obs, value$pred)
        names(result_delta) <- "delta.rm"

        results[[i]] <- c(result, result_rm2, results_reverse, result_average_rm2,
            result_delta)
    }
    return(results)
}

rf_10_CV <- function(x) {
    ok <- rf_cross_validation(x)
    data <- data.frame(ok)
```

```
    result <- mean_and_sd(data)
    df <- data.frame(result)
    R2_and_RMSE <- t(df)
    label <- c("RMSE_Mean", "Rsquared_Mean", "RM2_Mean", "Reverse_RM2_Mean",
        "Average_RM2_Mean", "Delta_RM2_Mean", "RMSE_SD", "Rsquared_SD", "RM2_SD",
        "Reverse_RM2_SD", "Average_RM2_SD", "Delta_RM2_SD")
    colnames(R2_and_RMSE) <- label
    return(R2_and_RMSE)
}


randomForest_testing <- function(x) {
    library(parallel)
    library(doSNOW)
    cl <- makeCluster(8)
    registerDoSNOW(cl)

    R2 <- function(y, equation, ...) {
        1 - (sum((y - predict(equation))^2)/sum((y - mean(y))^2))
    }
    rm2 <- function(y, x, ...) {
        if ((R2(y, (lm(y ~ x)))) > R2(y, (lm(y ~ -1 + x)))) {
            return(R2(y, (lm(y ~ x))) * (1 - (sqrt(R2(y, (lm(y ~ x))) - R2(y,
                (lm(y ~ -1 + x)))))))
        } else {
            return(R2(y, (lm(y ~ x))))
        }
    }
    rm2.reverse <- function(y, x, ...) {
        return(R2(x, (lm(x ~ y))) * (1 - (sqrt(R2(x, (lm(x ~ y))) - R2(x, (lm(x ~
            -1 + y)))))))
    }
    average.rm2 <- function(y, x, ...) {
        if ((R2(y, (lm(y ~ x)))) > R2(y, (lm(y ~ -1 + x)))) {
            return(((R2(y, (lm(y ~ x))) * (1 - (sqrt(R2(y, (lm(y ~ x))) - R2(y,
                (lm(y ~ -1 + x)))))) + R2(x, (lm(x ~ y))) * (1 - (sqrt(R2(x,
                (lm(x ~ y))) - R2(x, (lm(x ~ -1 + y))))))))/2)
        } else {
            return(((R2(y, (lm(y ~ x)))) + (R2(x, (lm(x ~ y))) * (1 - (sqrt(R2(x,
                (lm(x ~ y))) - R2(x, (lm(x ~ -1 + y))))))))/2)
        }
    }
    delta.rm2 <- function(y, x, ...) {
        if ((R2(y, (lm(y ~ x)))) > R2(y, (lm(y ~ -1 + x)))) {
            return(abs((R2(y, (lm(y ~ x))) * (1 - (sqrt(R2(y, (lm(y ~ x))) -
               R2(y, (lm(y ~ -1 + x)))))) - R2(x, (lm(x ~ y))) * (1 - (sqrt(R2(x,
                (lm(x ~ y))) - R2(x, (lm(x ~ -1 + y)))))))))
        } else {
            return(abs((R2(y, (lm(y ~ x)))) - (R2(x, (lm(x ~ y))) * (1 - (sqrt(R2(x,
                (lm(x ~ y))) - R2(x, (lm(x ~ -1 + y)))))))))
        }
    }
```

```r
    results <- list(100)
    results <- foreach(i = 1:100) %dopar% {

        x <- na.omit(x)
        para <- dplyr::sample_n(x, size = 2570, replace = TRUE)
        in_train_para <- sample(nrow(para), size = as.integer(nrow(para) * 0.8),
            replace = FALSE)
        Train <- para[in_train_para, ]
        Test <- para[-in_train_para, ]
        model_train <- ranger::ranger(pIC50 ~ ., data = Train, write.forest = TRUE,
            save.memory = TRUE)
        # actual <- train$Activity
        prediction <- predict(model_train, Test)
        prediction <- prediction$predictions
        value <- data.frame(obs = Test$pIC50, pred = prediction)
        rm(Train)
        rm(Test)
        rm(para)
        rm(in_train_para)
        rm(prediction)
        labeling <- c("obs", "pred")
        colnames(value) <- labeling
        result <- caret::defaultSummary(value)
        result_rm2 <- rm2(value$obs, value$pred)
        names(result_rm2) <- "rm2"
        results_reverse <- rm2.reverse(value$obs, value$pred)
        names(results_reverse) <- "reverse.rm2"
        result_average_rm2 <- average.rm2(value$obs, value$pred)
        names(result_average_rm2) <- "average.rm2"
        result_delta <- delta.rm2(value$obs, value$pred)
        names(result_delta) <- "delta.rm"

        results[[i]] <- c(result, result_rm2, results_reverse, result_average_rm2,
            result_delta)
    }
    return(results)
    stopCluster(cl)
}

randomForest_test <- function(x) {
    ok <- randomForest_testing(x)
    data <- data.frame(ok)
    result <- mean_and_sd(data)
    df <- data.frame(result)
    R2_and_RMSE <- t(df)
    label <- c("RMSE_Mean", "Rsquared_Mean", "RM2_Mean", "Reverse_RM2_Mean",
        "Average_RM2_Mean", "Delta_RM2_Mean", "RMSE_SD", "Rsquared_SD", "RM2_SD",
        "Reverse_RM2_SD", "Average_RM2_SD", "Delta_RM2_SD")
    colnames(R2_and_RMSE) <- label
    return(R2_and_RMSE)
}
```

**Training results for 12 data frame with random forest**

```r
input <- readRDS("data.Rds")
training_results <- lapply(input, function(x) {
  results <- randomForest_train(x)
  return(results)
})
training_results
```

```
## $AtomPairs2D_fingerPrintCount
##        RMSE_Mean Rsquared_Mean RM2_Mean Reverse_RM2_Mean Average_RM2_Mean
## result      0.51          0.92      0.8              0.7             0.75
##        Delta_RM2_Mean RMSE_SD Rsquared_SD RM2_SD Reverse_RM2_SD
## result            0.1    0.02        0.01   0.01           0.02
##        Average_RM2_SD Delta_RM2_SD
## result           0.01         0.01
##
## $AtomPairs2D_fingerPrinter
##        RMSE_Mean Rsquared_Mean RM2_Mean Reverse_RM2_Mean Average_RM2_Mean
## result      0.85          0.75     0.63             0.36             0.49
##        Delta_RM2_Mean RMSE_SD Rsquared_SD RM2_SD Reverse_RM2_SD
## result           0.28    0.06        0.03   0.02           0.05
##        Average_RM2_SD Delta_RM2_SD
## result           0.03         0.02
##
## $Substructure_fingerPrintCount
##        RMSE_Mean Rsquared_Mean RM2_Mean Reverse_RM2_Mean Average_RM2_Mean
## result       0.5          0.92     0.82             0.72             0.77
##        Delta_RM2_Mean RMSE_SD Rsquared_SD RM2_SD Reverse_RM2_SD
## result            0.1    0.02        0.01   0.01           0.02
##        Average_RM2_SD Delta_RM2_SD
## result           0.01         0.01
##
## $Substructure_fingerPrinter
##        RMSE_Mean Rsquared_Mean RM2_Mean Reverse_RM2_Mean Average_RM2_Mean
## result      0.85          0.75     0.64             0.36              0.5
##        Delta_RM2_Mean RMSE_SD Rsquared_SD RM2_SD Reverse_RM2_SD
## result           0.28    0.03        0.01   0.01           0.03
##        Average_RM2_SD Delta_RM2_SD
## result           0.02         0.01
##
## $Extended_finterPrinter
##        RMSE_Mean Rsquared_Mean RM2_Mean Reverse_RM2_Mean Average_RM2_Mean
## result      0.42          0.94     0.86             0.79             0.83
##        Delta_RM2_Mean RMSE_SD Rsquared_SD RM2_SD Reverse_RM2_SD
## result           0.07    0.03        0.01   0.01           0.02
##        Average_RM2_SD Delta_RM2_SD
## result           0.01         0.01
##
## $FingerPrinter
##        RMSE_Mean Rsquared_Mean RM2_Mean Reverse_RM2_Mean Average_RM2_Mean
## result      0.44          0.93     0.86             0.78             0.82
##        Delta_RM2_Mean RMSE_SD Rsquared_SD RM2_SD Reverse_RM2_SD
## result           0.07    0.04        0.01   0.01           0.02
```

```
##        Average_RM2_SD Delta_RM2_SD
## result           0.02         0.01
##
## $Estate_FingerPrinter
##        RMSE_Mean Rsquared_Mean RM2_Mean Reverse_RM2_Mean Average_RM2_Mean
## result         1          0.66     0.54             0.16             0.35
##        Delta_RM2_Mean RMSE_SD Rsquared_SD RM2_SD Reverse_RM2_SD
## result           0.38    0.05        0.03   0.02           0.04
##        Average_RM2_SD Delta_RM2_SD
## result           0.03         0.02
##
## $GraphOnly_FingerPrinter
##        RMSE_Mean Rsquared_Mean RM2_Mean Reverse_RM2_Mean Average_RM2_Mean
## result      0.61          0.87     0.77             0.63              0.7
##        Delta_RM2_Mean RMSE_SD Rsquared_SD RM2_SD Reverse_RM2_SD
## result           0.14    0.03        0.01   0.01           0.02
##        Average_RM2_SD Delta_RM2_SD
## result           0.02         0.01
##
## $KlekotaRoth_FingerprintCount
##        RMSE_Mean Rsquared_Mean RM2_Mean Reverse_RM2_Mean Average_RM2_Mean
## result      0.52          0.91     0.81             0.71             0.76
##        Delta_RM2_Mean RMSE_SD Rsquared_SD RM2_SD Reverse_RM2_SD
## result           0.11    0.03        0.01   0.01           0.02
##        Average_RM2_SD Delta_RM2_SD
## result           0.02         0.01
##
## $KlekotaRoth_FingerPrinter
##        RMSE_Mean Rsquared_Mean RM2_Mean Reverse_RM2_Mean Average_RM2_Mean
## result      0.59          0.89     0.78             0.64             0.71
##        Delta_RM2_Mean RMSE_SD Rsquared_SD RM2_SD Reverse_RM2_SD
## result           0.14    0.03        0.01   0.01           0.02
##        Average_RM2_SD Delta_RM2_SD
## result           0.02         0.01
##
## $MACCS_FingerPrinter
##        RMSE_Mean Rsquared_Mean RM2_Mean Reverse_RM2_Mean Average_RM2_Mean
## result      0.56          0.89     0.79             0.67             0.73
##        Delta_RM2_Mean RMSE_SD Rsquared_SD RM2_SD Reverse_RM2_SD
## result           0.12    0.03        0.01   0.01           0.02
##        Average_RM2_SD Delta_RM2_SD
## result           0.01         0.01
##
## $Pubchem_FingerPrinter
##        RMSE_Mean Rsquared_Mean RM2_Mean Reverse_RM2_Mean Average_RM2_Mean
## result      0.55           0.9      0.8             0.68             0.74
##        Delta_RM2_Mean RMSE_SD Rsquared_SD RM2_SD Reverse_RM2_SD
## result           0.12    0.03        0.01   0.01           0.02
##        Average_RM2_SD Delta_RM2_SD
## result           0.02         0.01
```

### 10-Fold CV results for 12 data frame with random forest

```r
input <- readRDS("data.Rds")
cross_validation_results <- lapply(input, function(x) {
  results <- rf_10_CV(x)
  return(results)
})
cross_validation_results
```

```
## $AtomPairs2D_fingerPrintCount
##        RMSE_Mean Rsquared_Mean RM2_Mean Reverse_RM2_Mean Average_RM2_Mean
## result      0.84          0.74     0.65             0.39             0.52
##        Delta_RM2_Mean RMSE_SD Rsquared_SD RM2_SD Reverse_RM2_SD
## result           0.26    0.15        0.07   0.06           0.11
##        Average_RM2_SD Delta_RM2_SD
## result           0.09         0.05
##
## $AtomPairs2D_fingerPrinter
##        RMSE_Mean Rsquared_Mean RM2_Mean Reverse_RM2_Mean Average_RM2_Mean
## result      1.03          0.61     0.53             0.17             0.35
##        Delta_RM2_Mean RMSE_SD Rsquared_SD RM2_SD Reverse_RM2_SD
## result           0.36    0.15        0.08   0.06            0.1
##        Average_RM2_SD Delta_RM2_SD
## result           0.08         0.04
##
## $Substructure_fingerPrintCount
##        RMSE_Mean Rsquared_Mean RM2_Mean Reverse_RM2_Mean Average_RM2_Mean
## result      0.77          0.78     0.71             0.49              0.6
##        Delta_RM2_Mean RMSE_SD Rsquared_SD RM2_SD Reverse_RM2_SD
## result           0.22    0.14        0.06   0.06            0.1
##        Average_RM2_SD Delta_RM2_SD
## result           0.08         0.05
##
## $Substructure_fingerPrinter
##        RMSE_Mean Rsquared_Mean RM2_Mean Reverse_RM2_Mean Average_RM2_Mean
## result         1          0.64     0.56             0.22             0.39
##        Delta_RM2_Mean RMSE_SD Rsquared_SD RM2_SD Reverse_RM2_SD
## result           0.34    0.13        0.06   0.05            0.1
##        Average_RM2_SD Delta_RM2_SD
## result           0.07         0.05
##
## $Extended_finterPrinter
##        RMSE_Mean Rsquared_Mean RM2_Mean Reverse_RM2_Mean Average_RM2_Mean
## result      0.76          0.79     0.72             0.52             0.62
##        Delta_RM2_Mean RMSE_SD Rsquared_SD RM2_SD Reverse_RM2_SD
## result            0.2    0.12        0.06   0.06            0.1
##        Average_RM2_SD Delta_RM2_SD
## result           0.08         0.05
##
## $FingerPrinter
##        RMSE_Mean Rsquared_Mean RM2_Mean Reverse_RM2_Mean Average_RM2_Mean
## result      0.76          0.79     0.71             0.52             0.62
##        Delta_RM2_Mean RMSE_SD Rsquared_SD RM2_SD Reverse_RM2_SD
## result            0.2    0.15        0.07   0.07           0.11
```

```
##        Average_RM2_SD Delta_RM2_SD
## result           0.09         0.06
##
## $Estate_FingerPrinter
##        RMSE_Mean Rsquared_Mean RM2_Mean Reverse_RM2_Mean Average_RM2_Mean
## result      1.11          0.55     0.47             0.07             0.27
##        Delta_RM2_Mean RMSE_SD Rsquared_SD RM2_SD Reverse_RM2_SD
## result            0.4    0.13        0.09   0.06           0.09
##        Average_RM2_SD Delta_RM2_SD
## result           0.07         0.04
##
## $GraphOnly_FingerPrinter
##        RMSE_Mean Rsquared_Mean RM2_Mean Reverse_RM2_Mean Average_RM2_Mean
## result      0.87          0.72     0.64             0.38             0.51
##        Delta_RM2_Mean RMSE_SD Rsquared_SD RM2_SD Reverse_RM2_SD
## result           0.27    0.13        0.06   0.06           0.09
##        Average_RM2_SD Delta_RM2_SD
## result           0.07         0.05
##
## $KlekotaRoth_FingerprintCount
##        RMSE_Mean Rsquared_Mean RM2_Mean Reverse_RM2_Mean Average_RM2_Mean
## result      0.79          0.78      0.7             0.49              0.6
##        Delta_RM2_Mean RMSE_SD Rsquared_SD RM2_SD Reverse_RM2_SD
## result           0.22    0.14        0.06   0.06            0.1
##        Average_RM2_SD Delta_RM2_SD
## result           0.08         0.05
##
## $KlekotaRoth_FingerPrinter
##        RMSE_Mean Rsquared_Mean RM2_Mean Reverse_RM2_Mean Average_RM2_Mean
## result      0.85          0.74     0.66             0.41             0.54
##        Delta_RM2_Mean RMSE_SD Rsquared_SD RM2_SD Reverse_RM2_SD
## result           0.25    0.14        0.07   0.06            0.1
##        Average_RM2_SD Delta_RM2_SD
## result           0.08         0.05
##
## $MACCS_FingerPrinter
##        RMSE_Mean Rsquared_Mean RM2_Mean Reverse_RM2_Mean Average_RM2_Mean
## result      0.81          0.77     0.68             0.45             0.57
##        Delta_RM2_Mean RMSE_SD Rsquared_SD RM2_SD Reverse_RM2_SD
## result           0.23    0.15        0.07   0.07           0.11
##        Average_RM2_SD Delta_RM2_SD
## result           0.09         0.05
##
## $Pubchem_FingerPrinter
##        RMSE_Mean Rsquared_Mean RM2_Mean Reverse_RM2_Mean Average_RM2_Mean
## result       0.8          0.76     0.69             0.46             0.57
##        Delta_RM2_Mean RMSE_SD Rsquared_SD RM2_SD Reverse_RM2_SD
## result           0.23    0.11        0.05   0.05           0.09
##        Average_RM2_SD Delta_RM2_SD
## result           0.07         0.04
```

**Testing results for 12 data frame with random forest**

```
input <- readRDS("data.Rds")
testing_results <- lapply(input, function(x) {
  results <- randomForest_test(x)
  return(results)
})
testing_results
```

```
## $AtomPairs2D_fingerPrintCount
##        RMSE_Mean Rsquared_Mean RM2_Mean Reverse_RM2_Mean Average_RM2_Mean
## result      0.82          0.76     0.67             0.42             0.54
##        Delta_RM2_Mean RMSE_SD Rsquared_SD RM2_SD Reverse_RM2_SD
## result           0.25     0.1        0.05   0.04           0.08
##        Average_RM2_SD Delta_RM2_SD
## result           0.06         0.04
##
## $AtomPairs2D_fingerPrinter
##        RMSE_Mean Rsquared_Mean RM2_Mean Reverse_RM2_Mean Average_RM2_Mean
## result      1.05           0.6     0.53             0.16             0.35
##        Delta_RM2_Mean RMSE_SD Rsquared_SD RM2_SD Reverse_RM2_SD
## result           0.36    0.12        0.06   0.05           0.08
##        Average_RM2_SD Delta_RM2_SD
## result           0.06         0.03
##
## $Substructure_fingerPrintCount
##        RMSE_Mean Rsquared_Mean RM2_Mean Reverse_RM2_Mean Average_RM2_Mean
## result      0.77          0.78     0.71              0.5             0.61
##        Delta_RM2_Mean RMSE_SD Rsquared_SD RM2_SD Reverse_RM2_SD
## result           0.21     0.1        0.05   0.05           0.08
##        Average_RM2_SD Delta_RM2_SD
## result           0.06         0.04
##
## $Substructure_fingerPrinter
##        RMSE_Mean Rsquared_Mean RM2_Mean Reverse_RM2_Mean Average_RM2_Mean
## result      0.98          0.65     0.57             0.25             0.41
##        Delta_RM2_Mean RMSE_SD Rsquared_SD RM2_SD Reverse_RM2_SD
## result           0.33    0.08        0.05   0.04           0.07
##        Average_RM2_SD Delta_RM2_SD
## result           0.05         0.03
##
## $Extended_finterPrinter
##        RMSE_Mean Rsquared_Mean RM2_Mean Reverse_RM2_Mean Average_RM2_Mean
## result      0.72          0.81     0.74             0.56             0.65
##        Delta_RM2_Mean RMSE_SD Rsquared_SD RM2_SD Reverse_RM2_SD
## result           0.18    0.08        0.04   0.04           0.06
##        Average_RM2_SD Delta_RM2_SD
## result           0.05         0.03
##
## $FingerPrinter
##        RMSE_Mean Rsquared_Mean RM2_Mean Reverse_RM2_Mean Average_RM2_Mean
## result      0.73           0.8     0.74             0.54             0.64
##        Delta_RM2_Mean RMSE_SD Rsquared_SD RM2_SD Reverse_RM2_SD
## result           0.19    0.09        0.04   0.04           0.07
```

```
##        Average_RM2_SD Delta_RM2_SD
## result           0.05         0.03
##
## $Estate_FingerPrinter
##        RMSE_Mean Rsquared_Mean RM2_Mean Reverse_RM2_Mean Average_RM2_Mean
## result       1.1          0.56     0.48             0.07             0.28
##        Delta_RM2_Mean RMSE_SD Rsquared_SD RM2_SD Reverse_RM2_SD
## result           0.41    0.08        0.05   0.03           0.06
##        Average_RM2_SD Delta_RM2_SD
## result           0.05         0.03
##
## $GraphOnly_FingerPrinter
##        RMSE_Mean Rsquared_Mean RM2_Mean Reverse_RM2_Mean Average_RM2_Mean
## result      0.87          0.72     0.65              0.4             0.53
##        Delta_RM2_Mean RMSE_SD Rsquared_SD RM2_SD Reverse_RM2_SD
## result           0.26    0.09        0.05   0.04           0.06
##        Average_RM2_SD Delta_RM2_SD
## result           0.05         0.03
##
## $KlekotaRoth_FingerprintCount
##        RMSE_Mean Rsquared_Mean RM2_Mean Reverse_RM2_Mean Average_RM2_Mean
## result      0.77          0.78     0.71              0.5             0.61
##        Delta_RM2_Mean RMSE_SD Rsquared_SD RM2_SD Reverse_RM2_SD
## result           0.21    0.11        0.05   0.05           0.09
##        Average_RM2_SD Delta_RM2_SD
## result           0.07         0.04
##
## $KlekotaRoth_FingerPrinter
##        RMSE_Mean Rsquared_Mean RM2_Mean Reverse_RM2_Mean Average_RM2_Mean
## result      0.81          0.76     0.68             0.45             0.56
##        Delta_RM2_Mean RMSE_SD Rsquared_SD RM2_SD Reverse_RM2_SD
## result           0.24     0.1        0.05   0.04           0.07
##        Average_RM2_SD Delta_RM2_SD
## result           0.06         0.03
##
## $MACCS_FingerPrinter
##        RMSE_Mean Rsquared_Mean RM2_Mean Reverse_RM2_Mean Average_RM2_Mean
## result       0.8          0.77     0.69             0.46             0.58
##        Delta_RM2_Mean RMSE_SD Rsquared_SD RM2_SD Reverse_RM2_SD
## result           0.23    0.09        0.04   0.04           0.07
##        Average_RM2_SD Delta_RM2_SD
## result           0.05         0.03
##
## $Pubchem_FingerPrinter
##        RMSE_Mean Rsquared_Mean RM2_Mean Reverse_RM2_Mean Average_RM2_Mean
## result      0.79          0.78      0.7             0.48             0.59
##        Delta_RM2_Mean RMSE_SD Rsquared_SD RM2_SD Reverse_RM2_SD
## result           0.22    0.08        0.03   0.04           0.07
##        Average_RM2_SD Delta_RM2_SD
## result           0.05         0.03
```
