# Supplementary material for: Probing the origins of human acetylcholinesterase inhibition via QSAR modeling and molecular docking
Source: PeerJ. 2016 Aug 9;4:e2322. doi: 10.7717/peerj.2322 (PMC4991866; doi:10.7717/peerj.2322)
Supplement: Data S2 [file peerj-04-2322-s003.zip › R mark down/Preprocessing.pdf]

Preprocessing

Saw Simeon, Nuttapat Anuwongcharoen, Watshara Shoombuatong, Aijaz Ahmad Malik, Virapong Prachayasittikul, Jarl E. S. Wikberg and Chanin Nantasenamat

June 7, 2016

Import data

Import the bioactivity data obtained from the ChEMBL database as a dataframe. Select the IC50 data subset.

```
library(readxl)
```

```
## Warning: package 'readxl' was built under R version 3.2.4
```

```
df_raw <- suppressWarnings(read_excel("Human_AChE.xlsx"))
names <- c("CMPD_CHEMBLID", "CANONICAL_SMILES", "STANDARD_TYPE",
           "RELATION", "STANDARD_VALUE", "STANDARD_UNITS",
           "PROTEIN_ACCESSION", "PREF_NAME",
      "PUBMED_ID",
      "JOURNAL", "YEAR", "VOLUME",
      "ISSUE", "FIRST_PAGE", "MOLWEIGHT",
      "ALOGP",
           "PSA", "NUM_RO5_VIOLATIONS")
df <- df_raw[, names]
df_1 <- subset(df, STANDARD_TYPE == "IC50")
df_2 <- subset(df_1, RELATION == "=")
df_4 <- subset(df_2, STANDARD_UNITS == "nM")
bioactivity <- paste0("Bioactivity data points: ", nrow(df))
IC50 <- paste0("IC50 Bioactivity data points: ", nrow(df_1))
IC50_omit <- paste0("Omit IC50 with lesser/greater than symbol: ", nrow(df_2))
final <- paste0("Final number of data points: ", nrow(df_4))

message <- c(bioactivity, IC50, IC50_omit, final)
print(message)
```

```
## [1] "Bioactivity data points: 9242"
## [2] "IC50 Bioactivity data points: 4910"
## [3] "Omit IC50 with lesser/greater than symbol: 3609"
## [4] "Final number of data points: 3596"
```

Remove redundant rows

```
ID <- df_4$CMPD_CHEMBLID
IC50_nm <- df_4$STANDARD_VALUE
smiles <- df_4$CANONICAL_SMILES
data_1 <- data.frame(ID, smiles, IC50_nm)

duplicate <- smiles[duplicated(smiles)]
unique <- unique(duplicate)
unique <- as.character(unique)
results <- data.frame()
```

```r
for (i in unique) {
    pre_data <- subset(data_1, smiles == i)
    results <- rbind(results, pre_data)
}


results_2 <- data.frame()
for (i in unique) {
    class <- subset(results, smiles == i)
    IC50 <- class$IC50_nm
    mean_IC50_nm <- mean(IC50)
    sd <- sd(IC50)
    data_frame <- data.frame(i, mean_IC50_nm, sd)
    results_2 <- rbind(data_frame, results_2)
}


keep <- subset(results_2, sd < 2)
names(keep) <- c("CANONICAL_SMILES", "STANDARD_VALUE", "SD")


non_redundant <- df_4[!duplicated(df_4$CANONICAL_SMILES), ]
non_redundant <- non_redundant[!non_redundant$CANONICAL_SMILES %in% unique,
    ]




non_redundant <- non_redundant[, c("CANONICAL_SMILES", "STANDARD_VALUE")]
redundant <- df_4[df_4$CANONICAL_SMILES %in% unique, ]
redundant <- subset(redundant, !duplicated(redundant[, c("CANONICAL_SMILES")]))
redundant <- subset(redundant, select = -STANDARD_VALUE)
STANDARD_VALUE <- keep$STANDARD_VALUE
CANONICAL_SMILES <- keep$CANONICAL_SMILES
cleaned_redundant <- data.frame(CANONICAL_SMILES, STANDARD_VALUE)
curated_data <- rbind(non_redundant, cleaned_redundant)
curated_data_na_removed <- na.omit(curated_data)

unique <- paste0("number of unique redundant compounds: ", length(unique))
sd_2 <- paste0("Compounds left that are kept", " which has a sd of lower than two: ",
    nrow(keep))
combined <- paste0("Combined data points: ", nrow(curated_data))
non_redundant_compound <- paste0("number of non-redundant compounds: ", nrow(non_redundant))
curated <- paste0("number of curated data points where missing smiles are removed: ",
    nrow(curated_data_na_removed))
message <- c(non_redundant_compound, unique, sd_2, combined, curated)
print(message)
```

```
## [1] "number of non-redundant compounds: 2426"
## [2] "number of unique redundant compounds: 340"
## [3] "Compounds left that are kept which has a sd of lower than two: 145"
## [4] "Combined data points: 2571"
## [5] "number of curated data points where missing smiles are removed: 2570"
```
