# Supplementary material for: Probing the origins of human acetylcholinesterase inhibition via QSAR modeling and molecular docking
Source: PeerJ. 2016 Aug 9;4:e2322. doi: 10.7717/peerj.2322 (PMC4991866; doi:10.7717/peerj.2322)

Y-permutation Plot

Saw Simeon, Nuttapat Anuwongcharoen, Watshara Shoombuatong, Aijaz Ahmad Malik, Virapong Prachayasittikul, Jarl E. S. Wikberg and Chanin Nantasenamat

June 10, 2016

Function For Creating the Scrambling Plot

```
scrambling_R2 <- function(x) {
    library(doSNOW)
    library(foreach)
    library(parallel)
    cl <- makeCluster(8)
    registerDoSNOW(cl)

    results <- list(50)
    results <- foreach(i = 1:50) %dopar% {
        x <- na.omit(x)
        para <- dplyr::sample_n(x, size = 2571, replace = TRUE)
        in_train_para <- sample(nrow(para), size = as.integer(nrow(para) * 0.8),
            replace = FALSE)
        Train <- para[in_train_para, ]
        Test <- para[-in_train_para, ]
        pIC50 <- gtools::permute(Train$pIC50)
        pIC50 <- data.frame(pIC50)
        fake_data <- cbind(pIC50, Train[, 2:ncol(Train)])
        # ctrl <- caret::trainControl(method = 'repeatedcv', number = 10, repeats =
        # 1) tune <- caret::train(pIC50~., data = fake_data, method = 'rf',
        # trControl = ctrl, tuneLength = 10)
        fit <- ranger::ranger(pIC50 ~ ., data = fake_data, write.forest = TRUE,
            save.memory = TRUE)
        # actual <- train$Activity
        prediction <- predict(fit, Train)
        prediction <- prediction$predictions
        value <- data.frame(obs = Train$pIC50, pred = prediction)

        rm(fit)
        rm(prediction)
        labeling <- c("obs", "pred")
        colnames(value) <- labeling
        results[[i]] <- caret::defaultSummary(value)
    }
    R2 <- data.frame(results)
    R2 <- t(R2)
    R2 <- as.numeric(R2[, 2])
    R2 <- round(R2, digits = 5)
    return(R2)
    stopCluster(cl)
}

real_R2 <- function(x) {
```

```
    x <- na.omit(x)
    para <- dplyr::sample_n(x, size = 2571, replace = TRUE)
    in_train_para <- sample(nrow(para), size = as.integer(nrow(para) * 0.8),
        replace = FALSE)
    Train <- para[in_train_para, ]
    Test <- para[-in_train_para, ]
    # ctrl <- caret::trainControl(method = 'repeatedcv', number = 10, repeats =
    # 1) tune <- train(pIC50~., data = x, method = 'rf', trControl = ctrl,
    # tuneLength = 10)
    fit <- ranger::ranger(pIC50 ~ ., data = Train, write.forest = TRUE, save.memory = TRUE)
    # actual <- train$Activity
    prediction <- predict(fit, Train)
    prediction <- prediction$predictions
    value <- data.frame(obs = Train$pIC50, pred = prediction)
    labeling <- c("obs", "pred")
    colnames(value) <- labeling
    result <- caret::defaultSummary(value)
    R2 <- as.data.frame(result)
    R2 <- R2[2, ]
    R2 <- round(R2, digits = 5)
    return(R2)
}

scrambling_Q2 <- function(x) {
    library(doSNOW)
    library(foreach)
    library(parallel)
    cl <- makeCluster(8)
    registerDoSNOW(cl)

    results <- list(50)
    results <- foreach(i = 1:50) %dopar% {
        para <- dplyr::sample_n(x, size = 2571, replace = TRUE)
        in_train_para <- sample(nrow(para), size = as.integer(nrow(para) * 0.8),
            replace = FALSE)
        Train <- para[in_train_para, ]
        Test <- para[-in_train_para, ]
        pIC50 <- gtools::permute(Train$pIC50)
        pIC50 <- data.frame(pIC50)
        myData <- cbind(pIC50, Train[, 2:ncol(x)])
        k = 10
        index <- sample(1:k, nrow(myData), replace = TRUE)
        folds <- 1:k
        myRes = data.frame()
        for (j in 1:k) {
            training <- subset(myData, index %in% folds[-j])
            testing <- subset(myData, index %in% c(j))
            # pIC50 <- gtools::permute(training$pIC50) pIC50 <- data.frame(pIC50)
            # fake_data <- cbind(pIC50, training[2:ncol(training)]) ctrl <-
            # caret::trainControl(method = 'repeatedcv', number = 10, repeats = 1) tune
            # <- caret::train(pIC50~., data = training, method = 'rf', trControl = ctrl,
            # tuneLength = 10)
            fit <- ranger::ranger(pIC50 ~ ., data = training, write.forest = TRUE,
```

```r
                save.memory = TRUE)
            # actual <- train$Activity
            prediction <- predict(fit, testing)
            prediction <- prediction$predictions
            value <- data.frame(obs = testing$pIC50, pred = prediction)

            # fit <- randomForest::randomForest(pIC50~., data = training, mtry =
            # tune$bestTune[[1]]) prediction <- predict(fit, testing) value <-
            # data.frame( obs = testing$pIC50, pred = prediction)
            myRes <- rbind(myRes, value)
        }
        value <- myRes
        labeling <- c("obs", "pred")
        rm(tune)
        rm(fit)
        rm(prediction)
        rm(ctrl)
        colnames(value) <- labeling
        results[[i]] <- caret::defaultSummary(value)
    }
    Q2 <- data.frame(results)
    Q2 <- t(Q2)
    Q2 <- as.numeric(Q2[, 2])
    Q2 <- round(Q2, digits = 5)
    return(Q2)
    stopCluster(cl)
}


real_Q2 <- function(x) {
    para <- dplyr::sample_n(x, size = 2571, replace = TRUE)
    in_train_para <- sample(nrow(para), size = as.integer(nrow(para) * 0.8),
        replace = FALSE)
    Train <- para[in_train_para, ]
    Test <- para[-in_train_para, ]
    myData <- Train
    k = 10
    index <- sample(1:k, nrow(myData), replace = TRUE)
    folds <- 1:k
    myRes <- data.frame()
    for (j in 1:k) {
        training <- subset(myData, index %in% folds[-j])
        testing <- subset(myData, index %in% c(j))
        # ctrl <- caret::trainControl(method = 'repeatedcv', number = 10, repeats =
        # 1) tune <- train(pIC50~., data = training, method = 'rf', trControl =
        # ctrl, tuneLength = 10)
        fit <- ranger::ranger(pIC50 ~ ., data = training, write.forest = TRUE,
            save.memory = TRUE)
        # actual <- train$Activity
        prediction <- predict(fit, testing)
        prediction <- prediction$predictions
        value <- data.frame(obs = testing$pIC50, pred = prediction)
        # fit <- randomForest(pIC50~., data = training, mtry = tune$bestTune[[1]])
```

```r
        # prediction <- predict(fit, testing) value <- data.frame(obs =
        # testing$pIC50, pred = prediction)
        myRes <- rbind(myRes, value)
    }
    value <- myRes
    labeling <- c("obs", "pred")
    colnames(value) <- labeling
    result <- caret::defaultSummary(value)
    Q2 <- as.data.frame(result)
    Q2 <- Q2[2, ]
    Q2 <- round(Q2, digits = 5)
    return(Q2)
}

data_pre <- function(x) {
    fake_R2 <- scrambling_R2(x)
    real_R2 <- real_R2(x)
    fake_Q2 <- scrambling_Q2(x)
    real_Q2 <- real_Q2(x)
    fake_R2 <- as.data.frame(fake_R2)
    fake_R2$Label <- "Fake"
    real_R2 <- as.data.frame(real_R2)
    real_R2$Label <- "Real"
    fake_Q2 <- as.data.frame(fake_Q2)
    fake_Q2$Label <- "Fake"
    real_Q2 <- as.data.frame(real_Q2)
    real_Q2$Label <- "Real"
    fake <- cbind(fake_R2, fake_Q2)
    real <- cbind(real_R2, real_Q2)
    combine <- data.frame(fake, real)
    return(combine)
}

plot_scrambling <- function(x) {
    ok <- data_pre(x)
    colnames(ok) <- c("R2", "Label", "Q2", "Label", "R2", "Label", "Q2", "Label")
    R2 <- ok[c(1, 5)]
    R2 <- reshape2::melt(R2)
    R2 <- R2$value
    R2 <- data.frame(R2)
    Q2 <- ok[c(3, 7)]
    Q2 <- reshape2::melt(Q2)
    Q2 <- Q2$value
    Q2 <- data.frame(Q2)
    Label <- c("Fake", "Fake", "Fake", "Fake", "Fake", "Fake", "Fake", "Fake",
        "Fake", "Fake", "Fake", "Fake", "Fake", "Fake", "Fake", "Fake", "Fake",
        "Fake", "Fake", "Fake", "Fake", "Fake", "Fake", "Fake", "Fake", "Fake",
        "Fake", "Fake", "Fake", "Fake", "Fake", "Fake", "Fake", "Fake", "Fake",
        "Fake", "Fake", "Fake", "Fake", "Fake", "Fake", "Fake", "Fake", "Fake",
        "Fake", "Fake", "Fake", "Fake", "Fake", "Fake", "Real", "Real", "Real",
        "Real", "Real", "Real", "Real", "Real", "Real", "Real", "Real", "Real",
        "Real", "Real", "Real", "Real", "Real", "Real", "Real", "Real", "Real",
        "Real", "Real", "Real", "Real", "Real", "Real", "Real", "Real", "Real",
```

```
        "Real", "Real", "Real", "Real", "Real", "Real", "Real", "Real", "Real",
        "Real", "Real", "Real", "Real", "Real", "Real", "Real", "Real", "Real",
        "Real", "Real")
    data <- cbind(R2, Q2, Label)
    library(ggplot2)
    plot <- ggplot(data, aes(x = R2, y = Q2, colour = Label)) + geom_point(size = 7,
        colour = "black", aes(fill = factor(Label)), pch = 21, alpha = 0.8) +
        theme(legend.position = ("none"), axis.text = element_blank(), panel.border = element_rect(line
            colour = "black", fill = NA, size = 1)) + xlab("") + ylab("") +
        # labs(y = expression(paste(italic(Q^2)))) + labs(x =
    # expression(paste(italic(R^2)))) +
    scale_x_continuous(limits = c(0, 1), breaks = seq(from = 0, to = 1, by = 0.5)) +
        scale_y_continuous(limits = c(0, 1), breaks = seq(from = 0, to = 1,
            by = 0.5))
    # coord_cartesian(ylim = c(0, 1), xlim = c(0, 1))
    return(plot)
}
```

CDK

```
input <- readRDS("data.Rds")
CDK_Fingerprint <- input$FingerPrinter
plot_scrambling(CDK_Fingerprint)
```

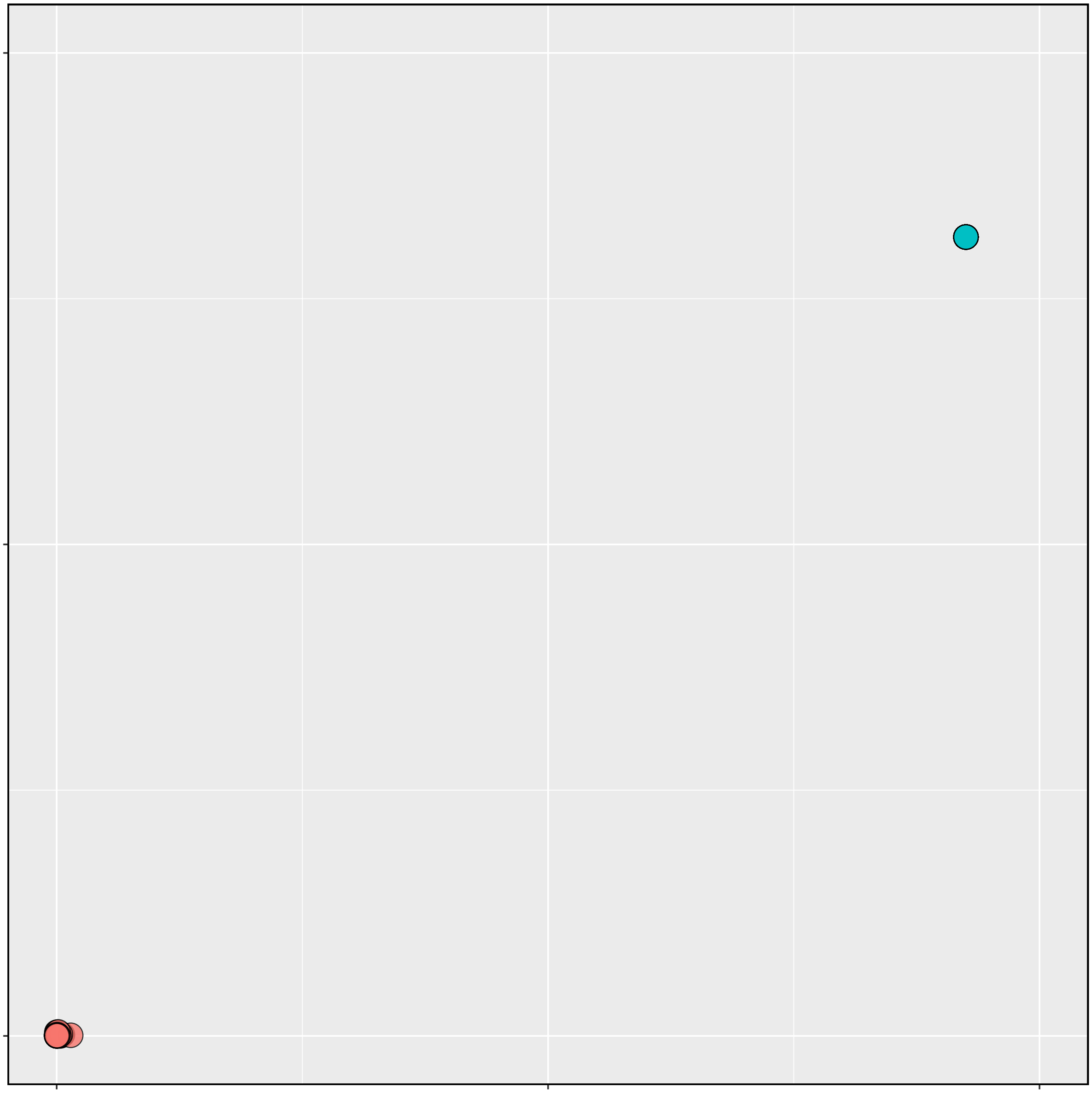

CDK Extended

```
input <- readRDS("data.Rds")
Extended_CDK_Fingerprint <- input$Extended_finterPrinter
plot_scrambling(Extended_CDK_Fingerprint)
```

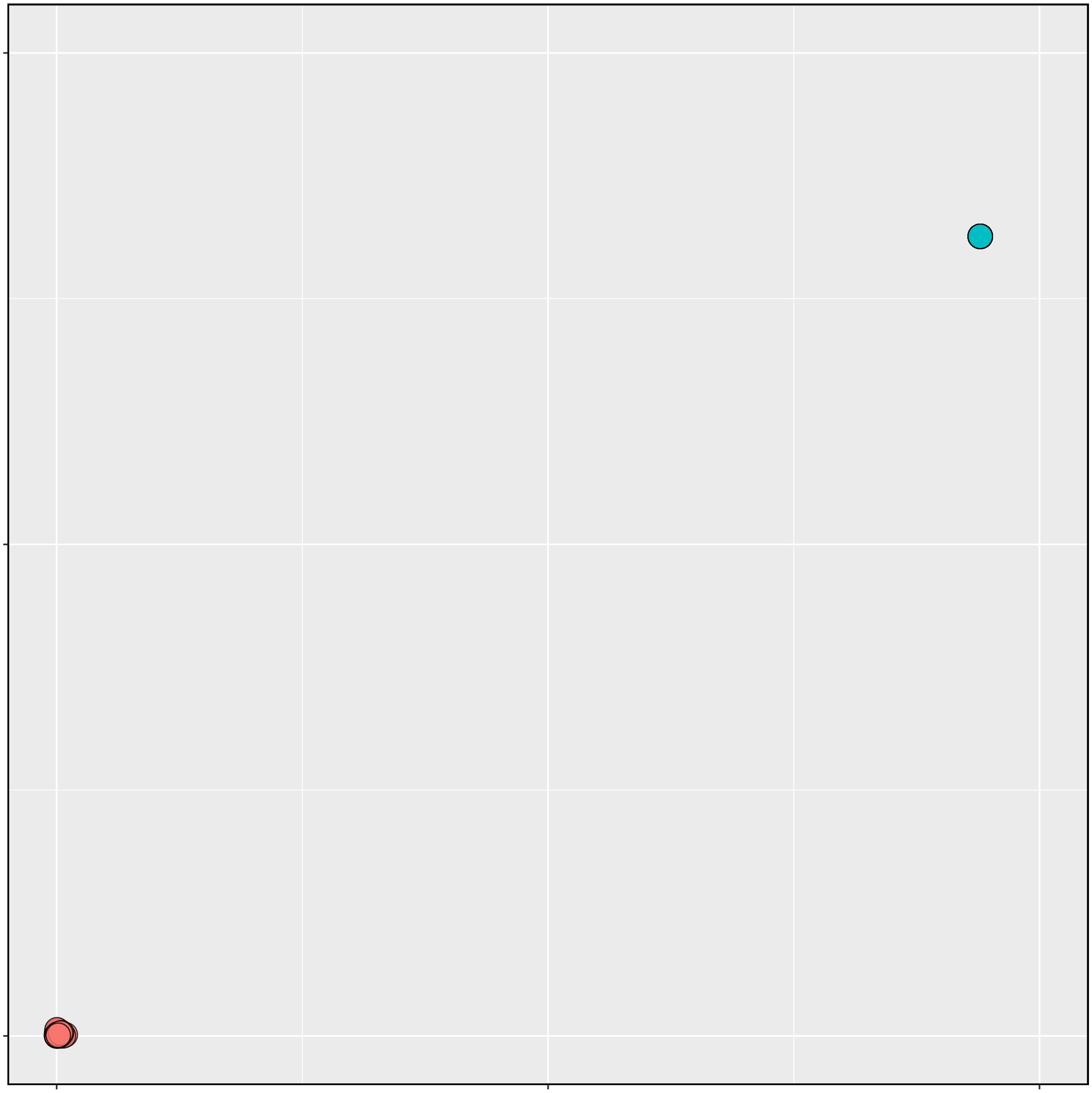

CDK Graph Only

```
input <- readRDS("data.Rds")
Graph_CDK_Fingerprint <- input$GraphOnly_FingerPrinter
plot_scrambling(Graph_CDK_Fingerprint)
```

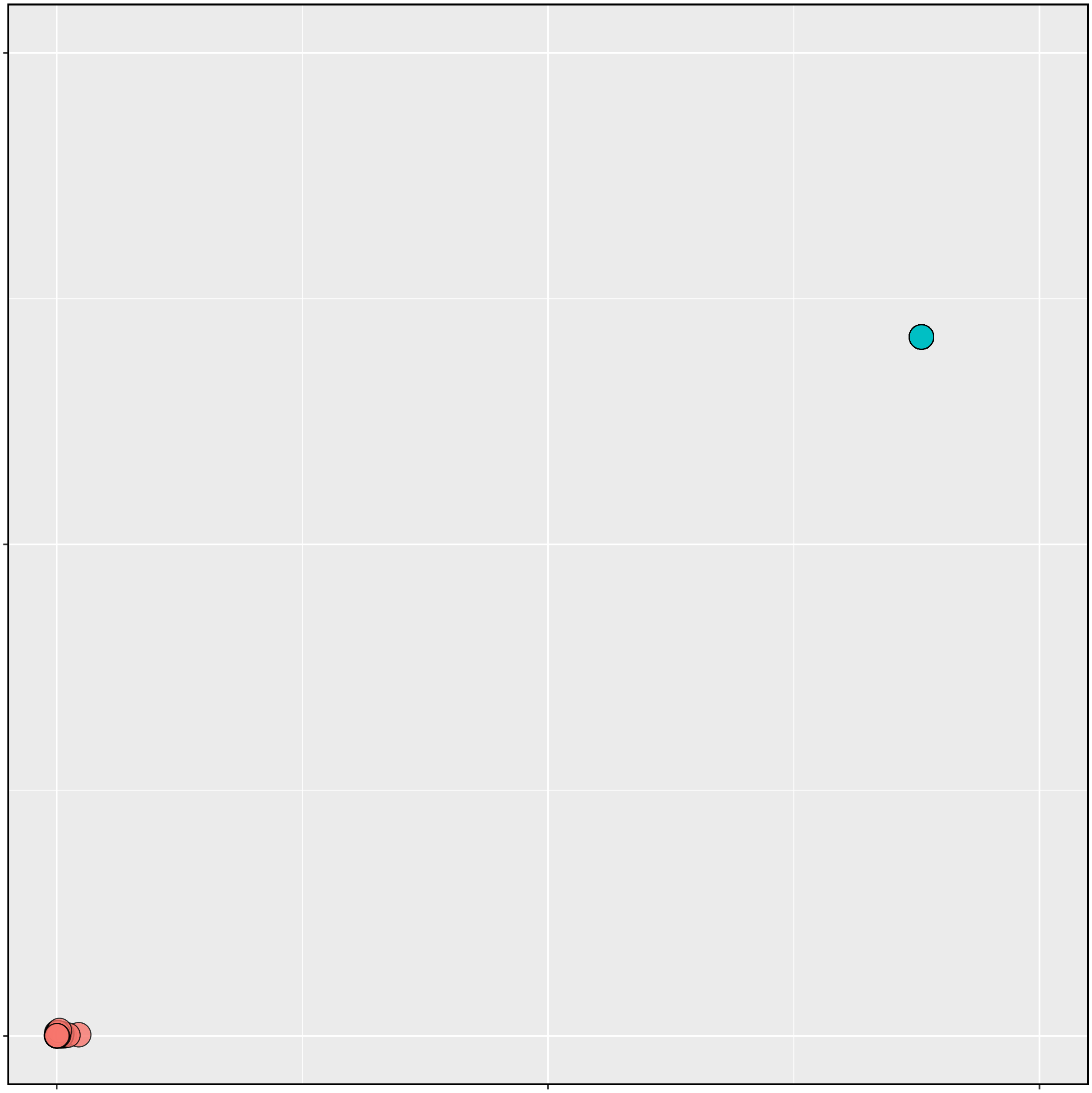

E-State

```
input <- readRDS("data.Rds")
E_state_Fingerprint <- input$Estate_FingerPrinter
plot_scrambling(E_state_Fingerprint)
```

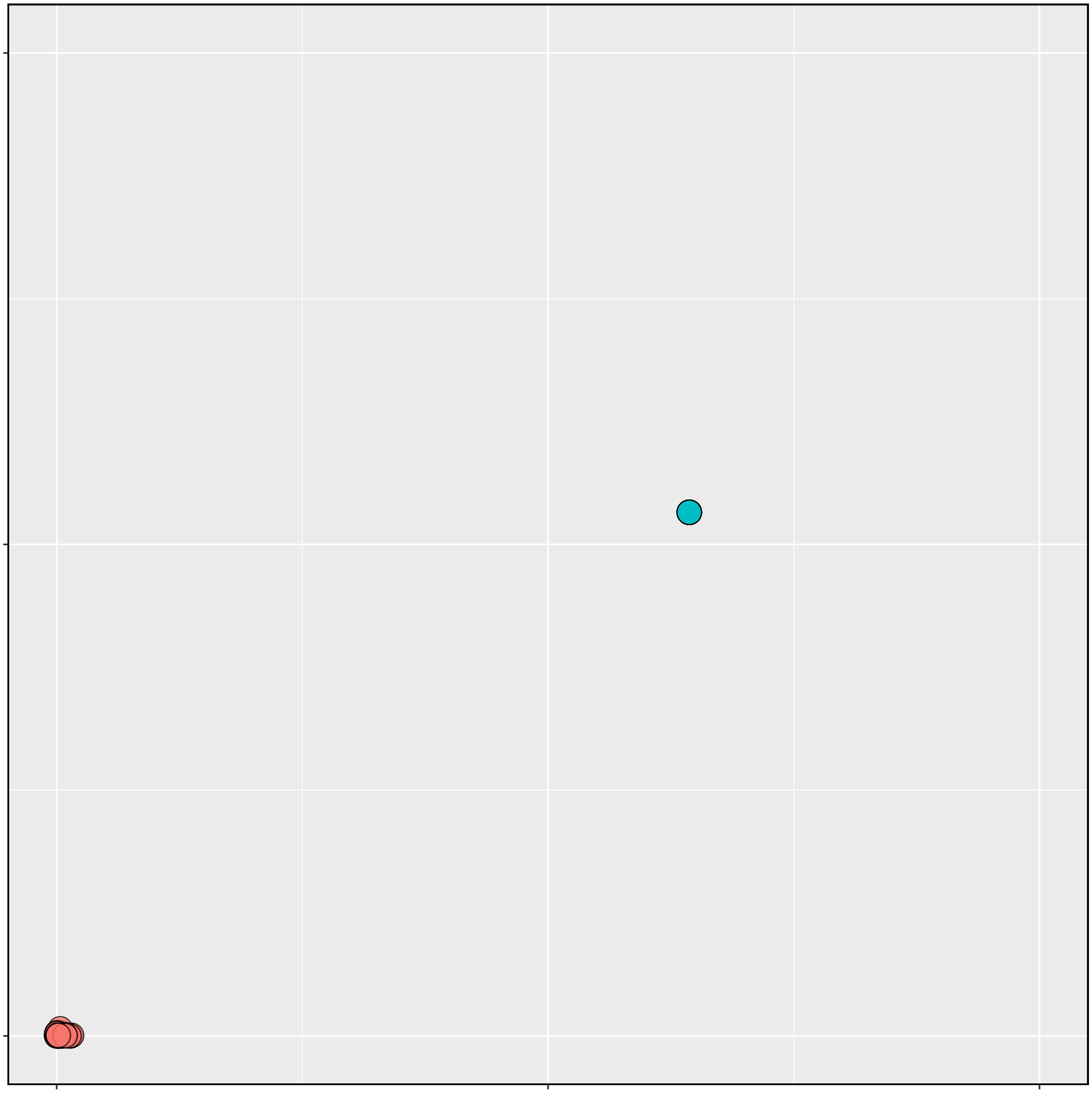

MACCS

```
input <- readRDS("data.Rds")
MACCS_Fingerprint <- input$MACCS_FingerPrinter
plot_scrambling(MACCS_Fingerprint)
```

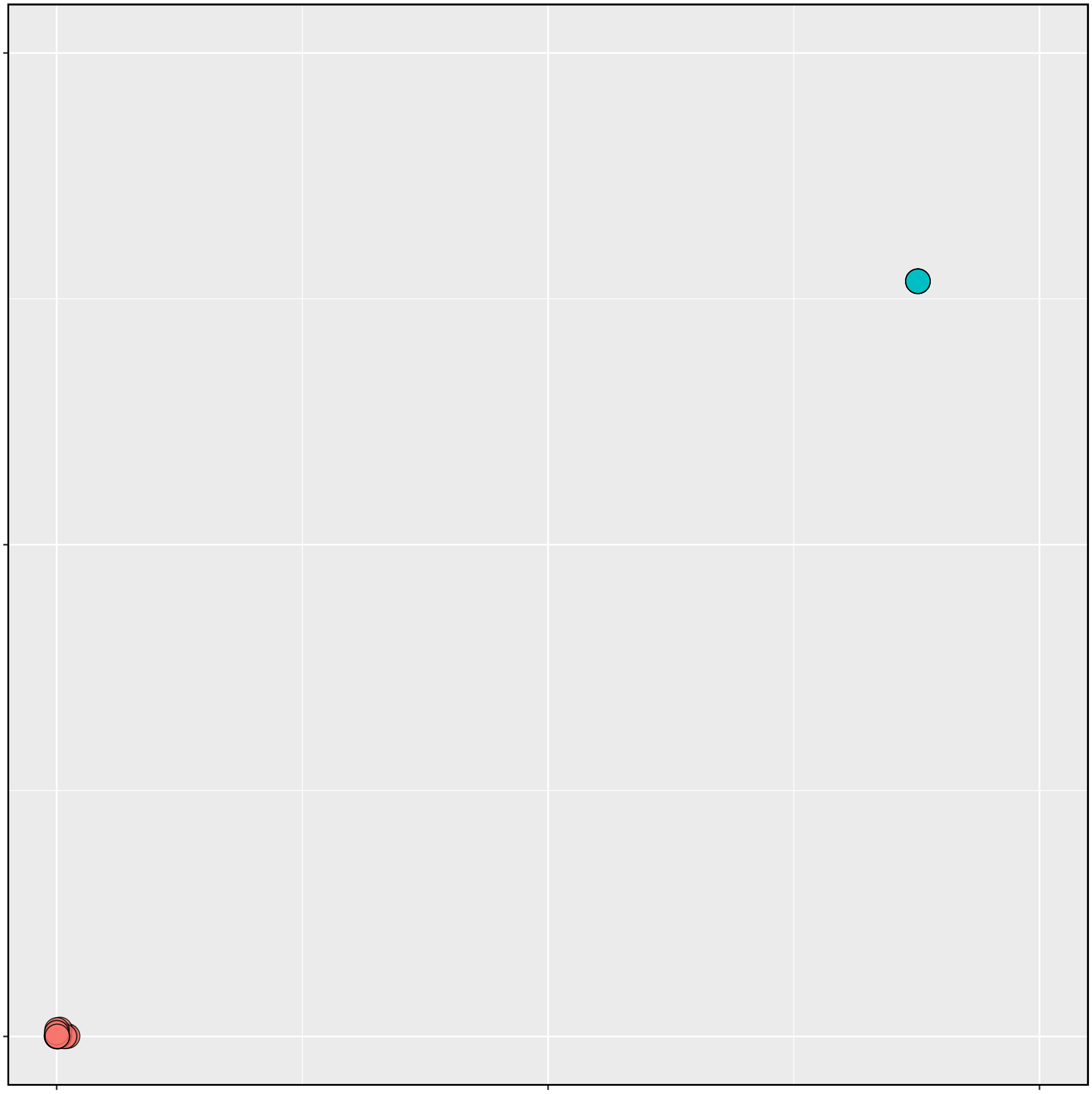

PubChem

```
input <- readRDS("data.Rds")
PubChem_Fingerprint <- input$Pubchem_FingerPrinter
plot_scrambling(PubChem_Fingerprint)
```

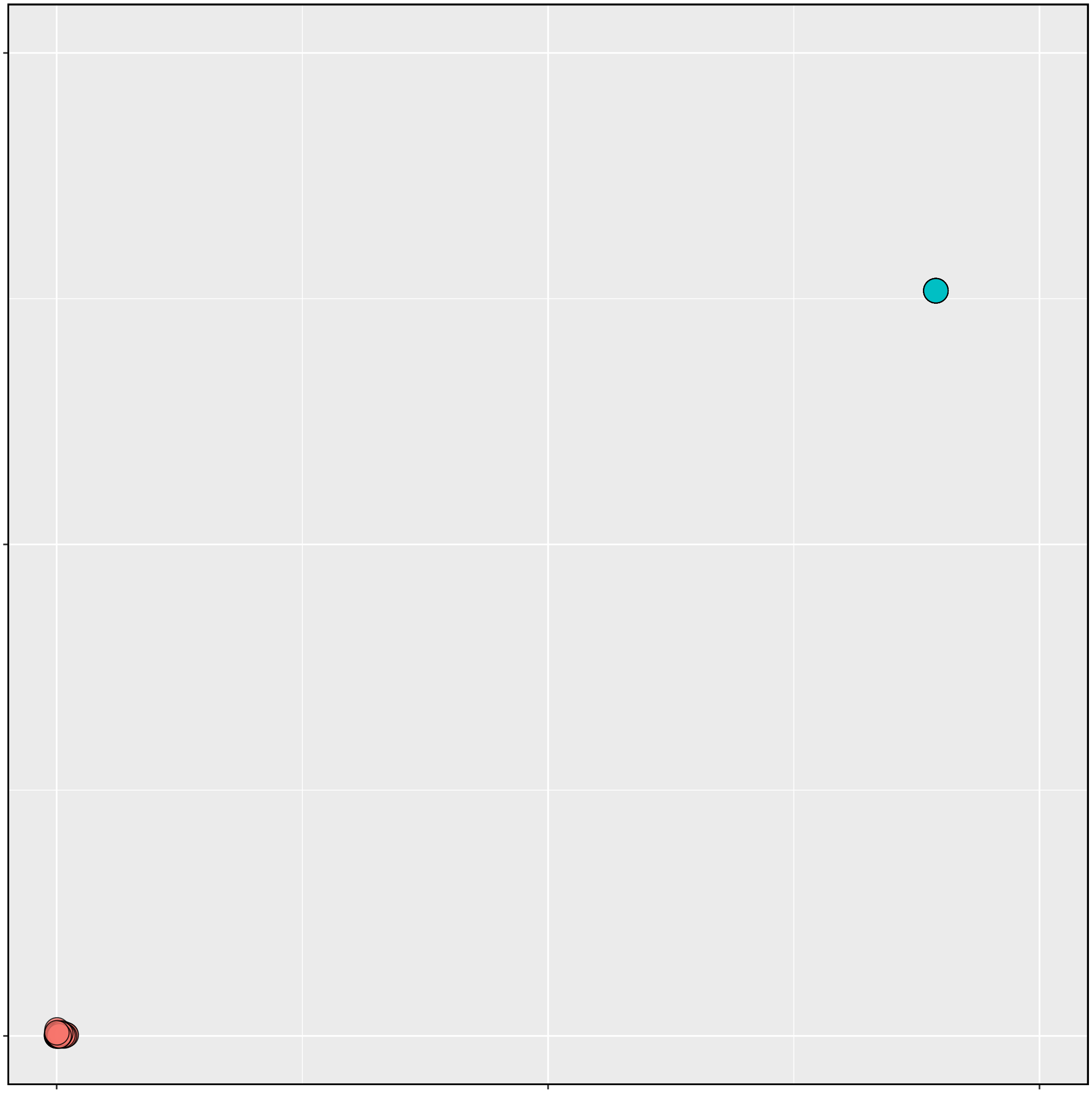

Substructure

```
input <- readRDS("data.Rds")
Substructure <- input$Substructure_fingerPrinter
plot_scrambling(Substructure)
```

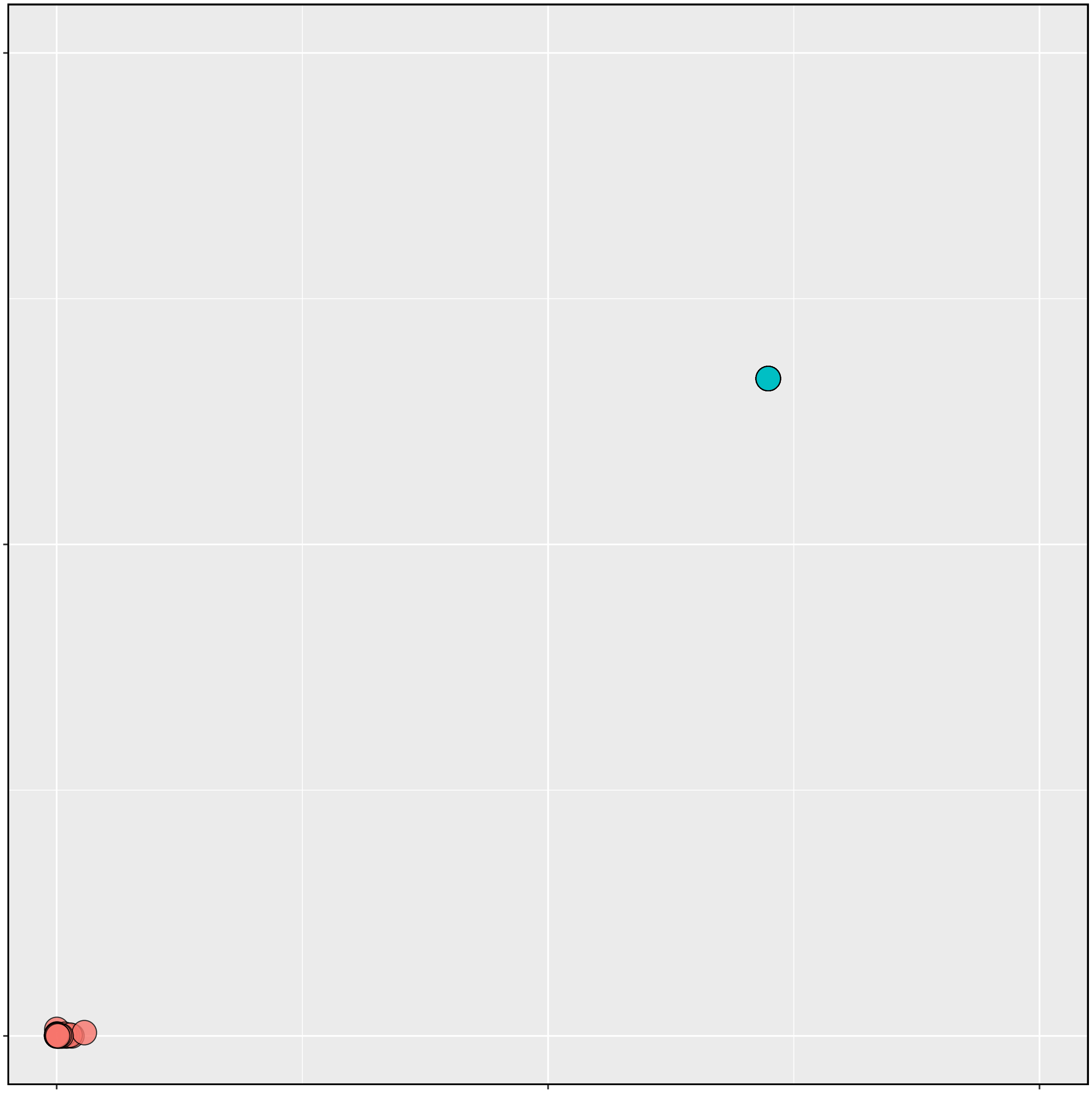

Substructure Count

```
input <- readRDS("data.Rds")
Substructure_Count <- input$Substructure_fingerPrintCount
plot_scrambling(Substructure_Count)
```

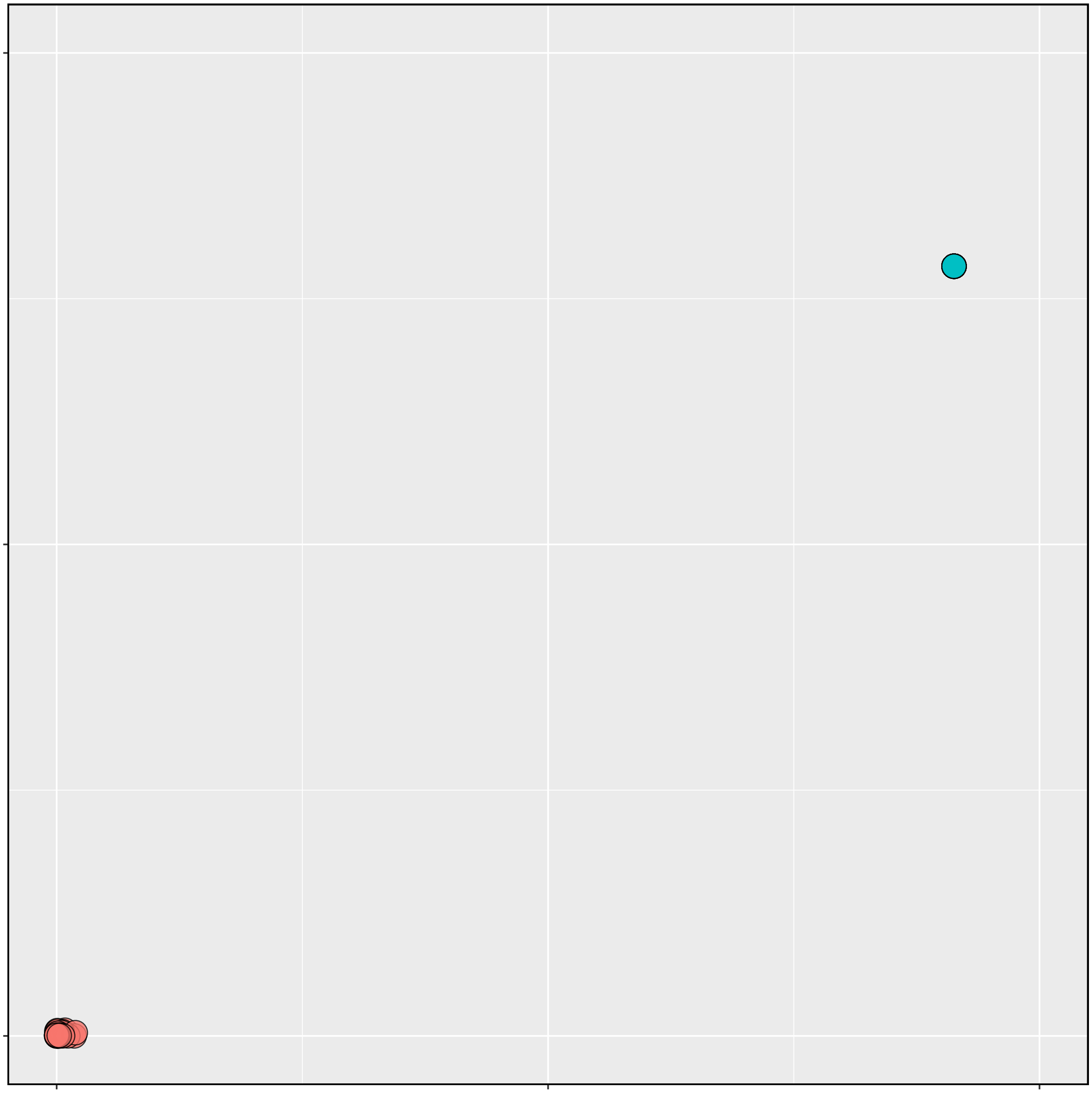

Klekota-Roth

```
input <- readRDS("data.Rds")
Klekota_Roth <- input$KlekotaRoth_FingerPrinter
plot_scrambling(Klekota_Roth)
```

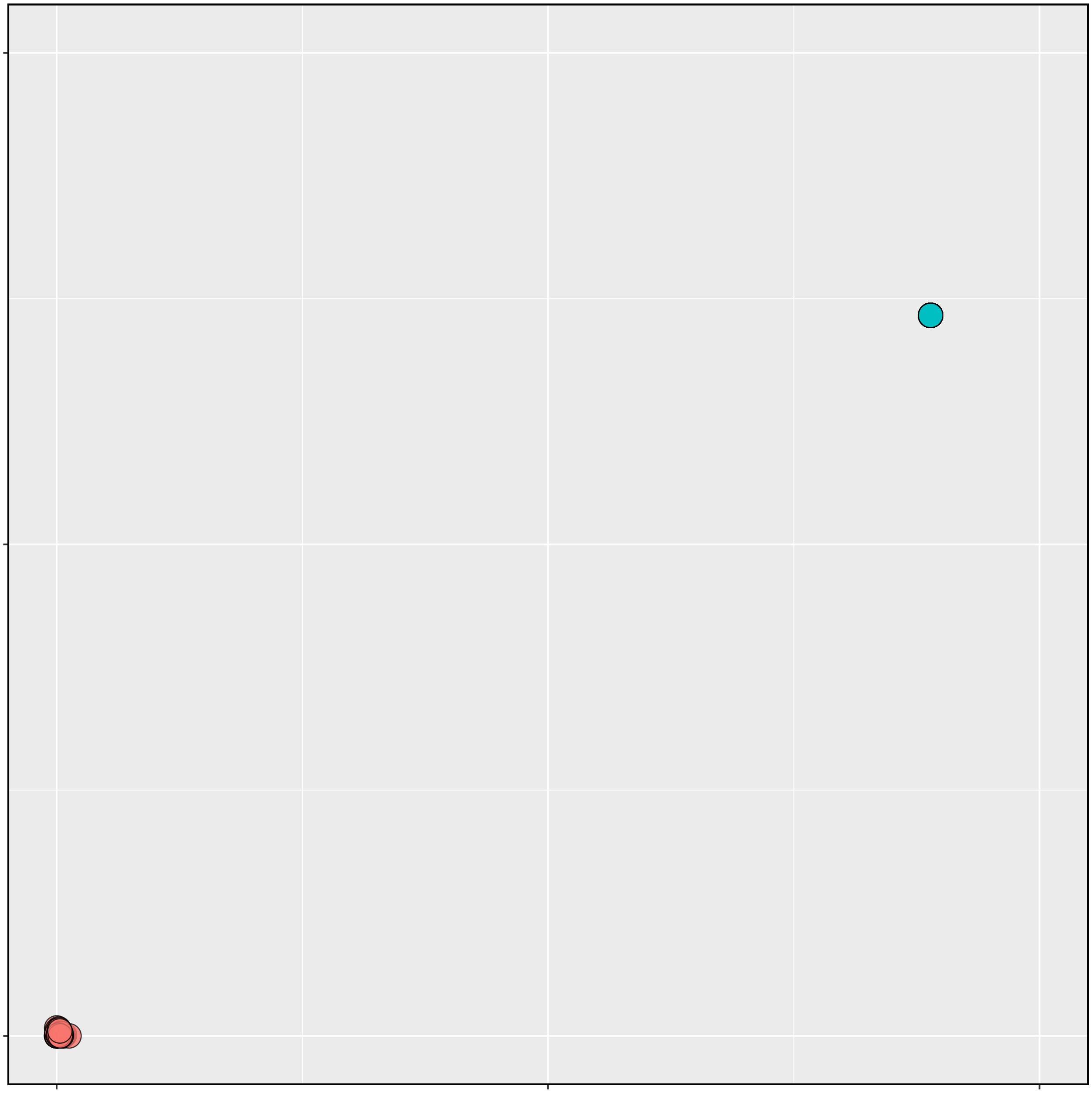

Klekota-Roth Count

```r
input <- readRDS("data.Rds")
Klekota_Roth_Count <- input$KlekotaRoth_FingerprintCount
plot_scrambling(Klekota_Roth_Count)
```

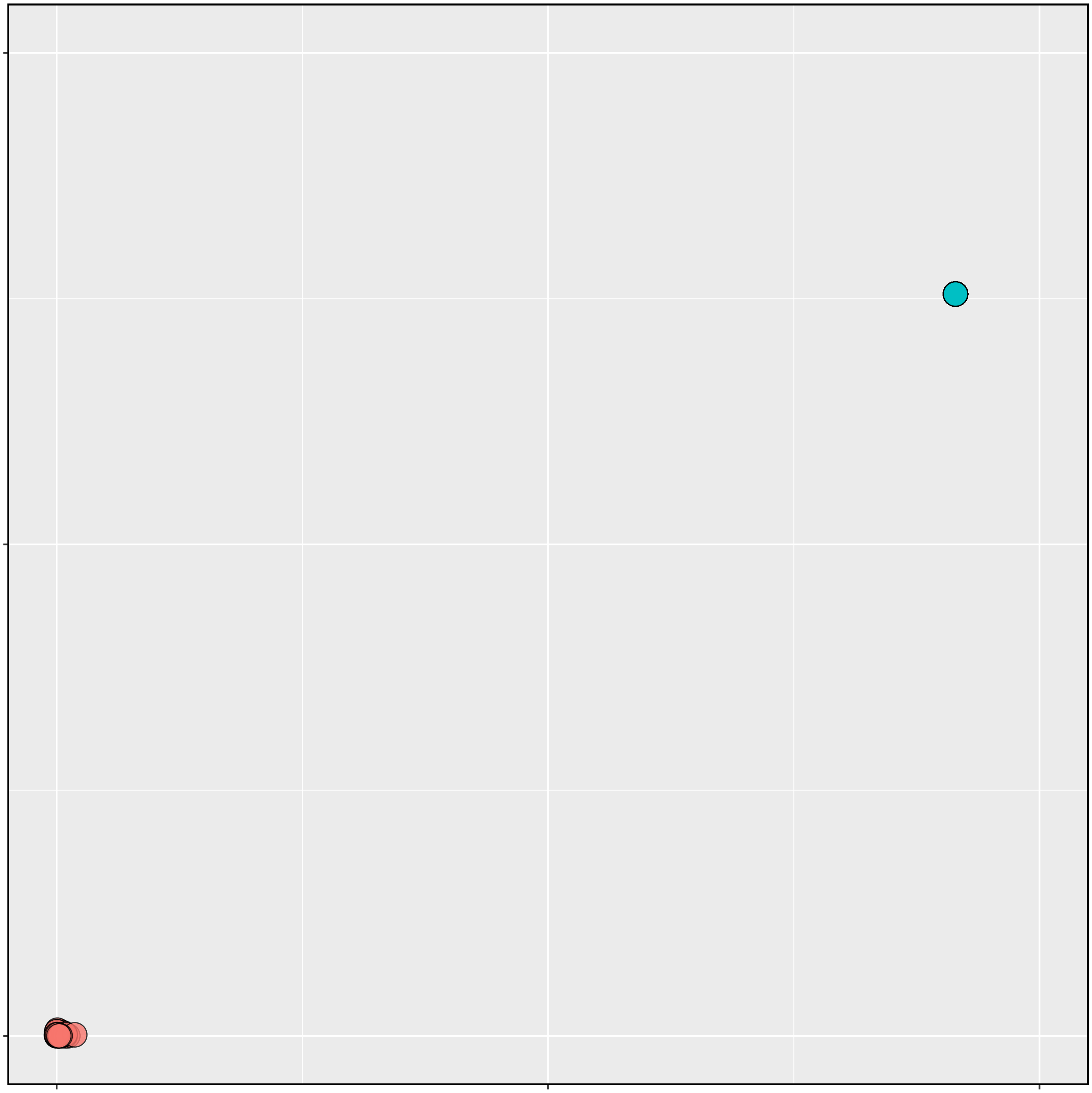

2D Atom Pairs

```
input <- readRDS("data.Rds")
Atom_Pairs <- input$AtomPairs2D_fingerPrinter
plot_scrambling(Atom_Pairs)
```

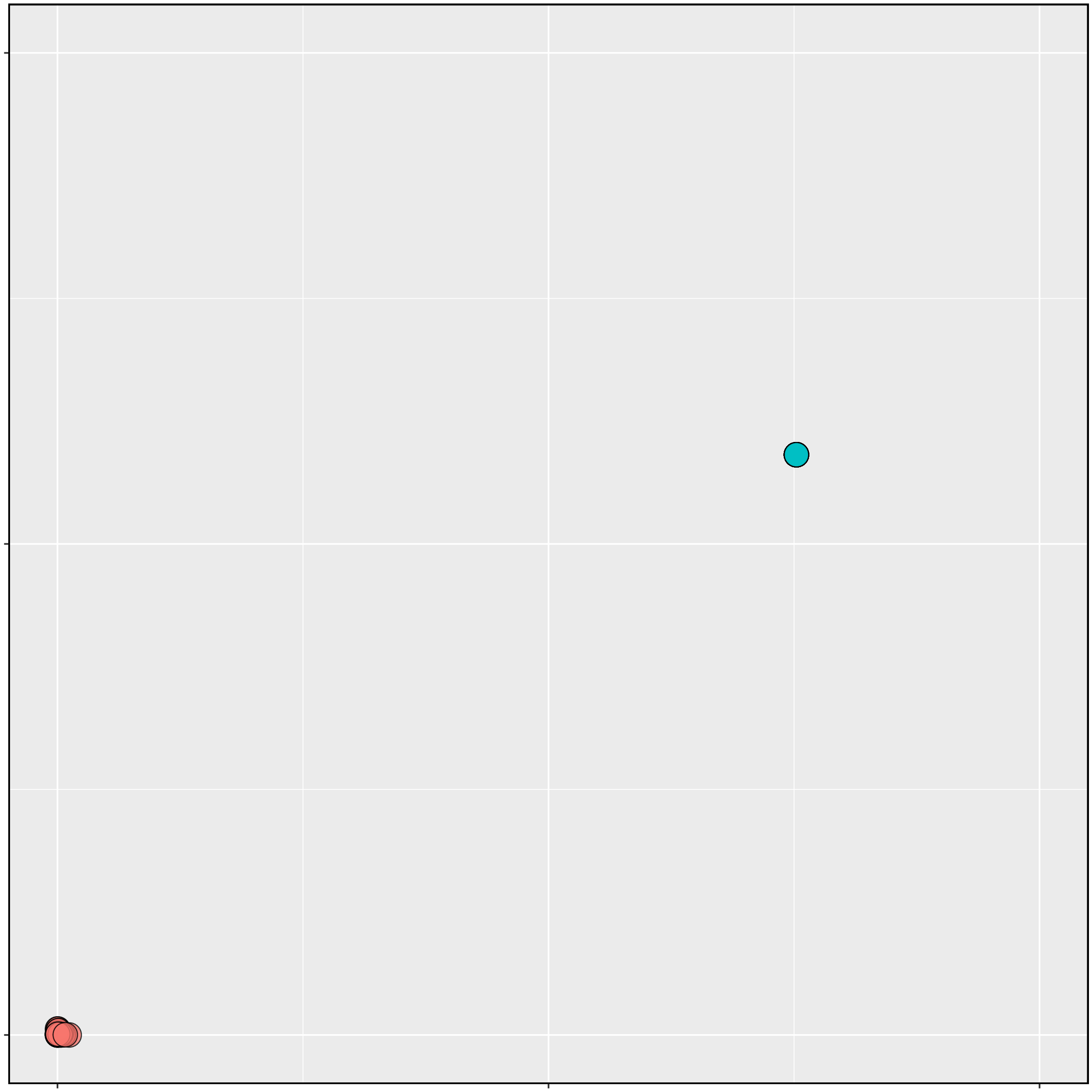

2D Atom Pairs Count

```
input <- readRDS("data.Rds")
Atom_Pairs_Count <- input$AtomPairs2D_fingerPrintCount
plot_scrambling(Atom_Pairs_Count)
```

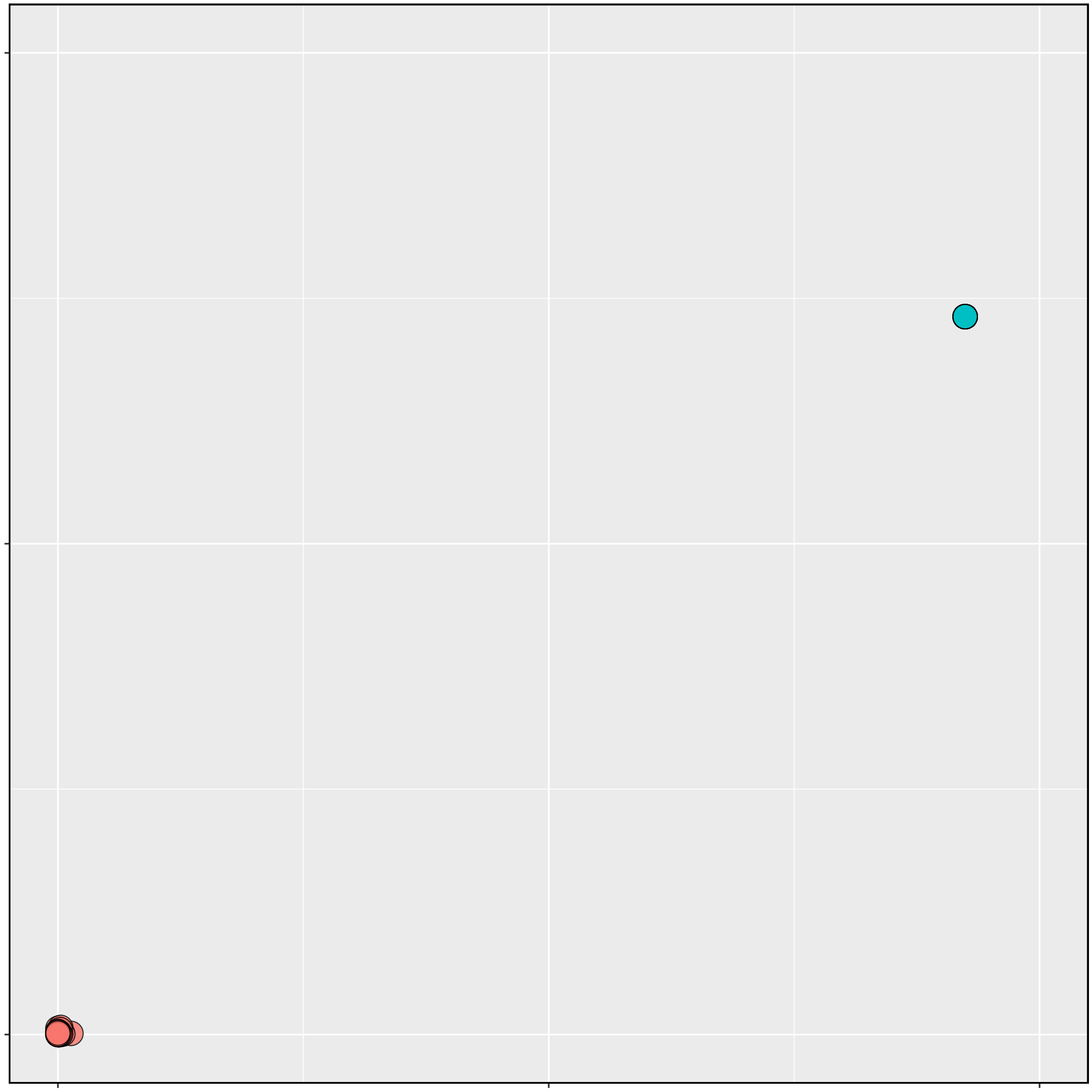

Supplement: Data S2 [file peerj-04-2322-s003.zip › R mark down/Scrambling.pdf]
