# Supplementary material for: Probing the origins of human acetylcholinesterase inhibition via QSAR modeling and molecular docking
Source: PeerJ. 2016 Aug 9;4:e2322. doi: 10.7717/peerj.2322 (PMC4991866; doi:10.7717/peerj.2322)

Applicability Domain (Williams Plot)

Saw Simeon, Nuttapat Anuwongcharoen, Watshara Shoombuatong, Aijaz Ahmad Malik, Virapong Prachayasittikul, Jarl E. S. Wikberg and Chanin Nantasenamat

June 19, 2016

Function to create Williams Plot (Red = External, Cyan = Internal)

```
file <- function(x) {
  library(randomForest)
  library(caret)
  library(ranger)
  set.seed(10)
  para <- dplyr::sample_n(x, size = 2570, replace = TRUE)
  set.seed(3)
  in_train_para <- sample(nrow(para),
                          size = as.integer(nrow(para) * 0.8),
                          replace = FALSE)
  set.seed(4)
  Train <- para[in_train_para, ]
  Test  <- para[-in_train_para, ]

  model <- ranger::ranger(pIC50~., data = Train, write.forest = TRUE, save.memory = TRUE)
  #actual <- train$Activity
  prediction <- predict(model, Train)
  prediction_Internal <- prediction$predictions
  value <- data.frame(obs = Train$pIC50, pred = prediction_Internal)
  labeling <- c("obs", "pred")
  colnames(value) <- labeling
  value$Label <- c("Internal")
  prediction_External <- predict(model, Test)
  prediction_External <- prediction_External$predictions
  value_external <- data.frame(obs = Test$pIC50, pred = prediction_External)
  colnames(value_external) <- labeling
  value_external$Label <- c("External")
  results <- rbind(value, value_external)
  return(results)
}




get_leverage <- function(x) {
  file <- file(x)
  x <- file[, 1]
  y <- file[, 2]
  data <- data.frame(x, y)
  error <- y-x
  label <- file[3]
```

```
  fit = lm(y~x,data = data)
  hv <- as.data.frame(hatvalues(fit))
  std.error = scale(error)
  df <- data.frame(hv, std.error, label)
  names(df) <- c("hv", "std.error", "Label")
  return(df)
}



plot_william <- function(x, title) {
  library(ggplot2)
  library(cowplot)
  ok <- get_leverage(x)
  df <- data.frame(ok)
  good <- ggplot(df, aes(hv, std.error)) +
  geom_point(aes(color = Label), alpha = .20, size = 6) +
  ggtitle(title) +
       xlab("Leverage") + ylab("Standardized Residuals") +
       geom_hline(yintercept = 3, color = "red", linetype = 2) +
    geom_hline(yintercept = -3, color = "red", linetype = 2) +
     theme(
      panel.border = element_rect(linetype = "solid", colour = "black",
                                  fill = NA, size = 1),

    plot.title = element_text(size = 30, color = "black", face = "bold"),
      axis.text.y = element_text(size = 20, colour = "black"),
    axis.text.x = element_text(size = 20, colour = "black"),
    axis.title.x = element_text(size = 30, color = "black", face = "bold"),
    axis.title.y = element_text(size = 30, color = "black", face = "bold"),

      legend.position = ("none"))
  return(good)

}
```

CDK fingerprint

```
input <- readRDS("data.Rds")
df <- input$FingerPrinter
plot <- plot_william(df, title = "CDK Fingerprint")
h <- 3*((dim(df)[2] - 1) + 1) / dim(df)[1]*0.8
plot + geom_vline(xintercept = h, linetype = 2, color = "red")
```

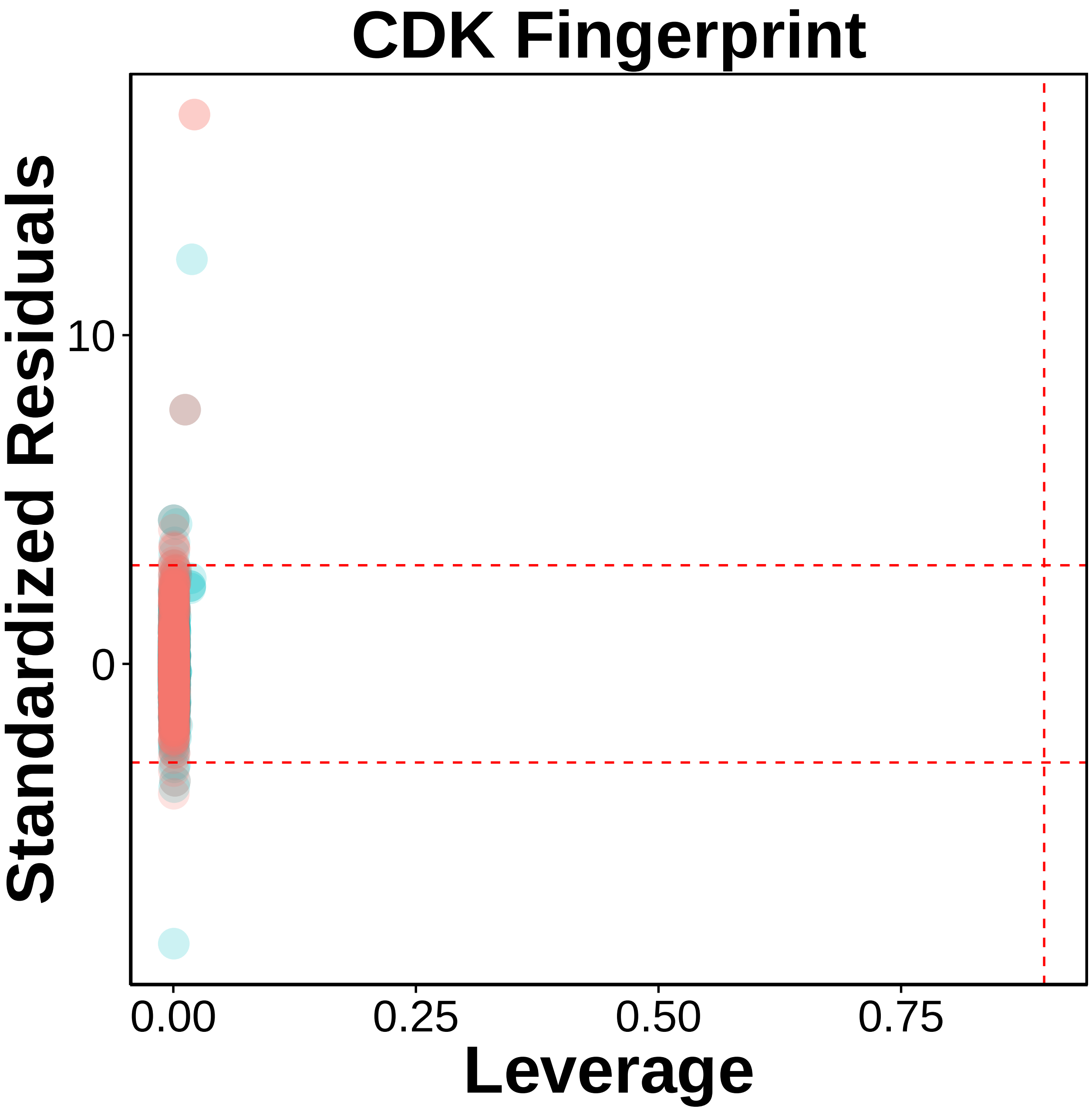

CDK extended fingerprint

```r
input <- readRDS("data.Rds")
df <- input$Extended_finterPrinter
plot <- plot_william(df, title = "CDK Extended Fingerprint")
h <- 3*((dim(df)[2] - 1) + 1) / dim(df)[1]*0.8
plot + geom_vline(xintercept = h, linetype = 2, color = "red")
```

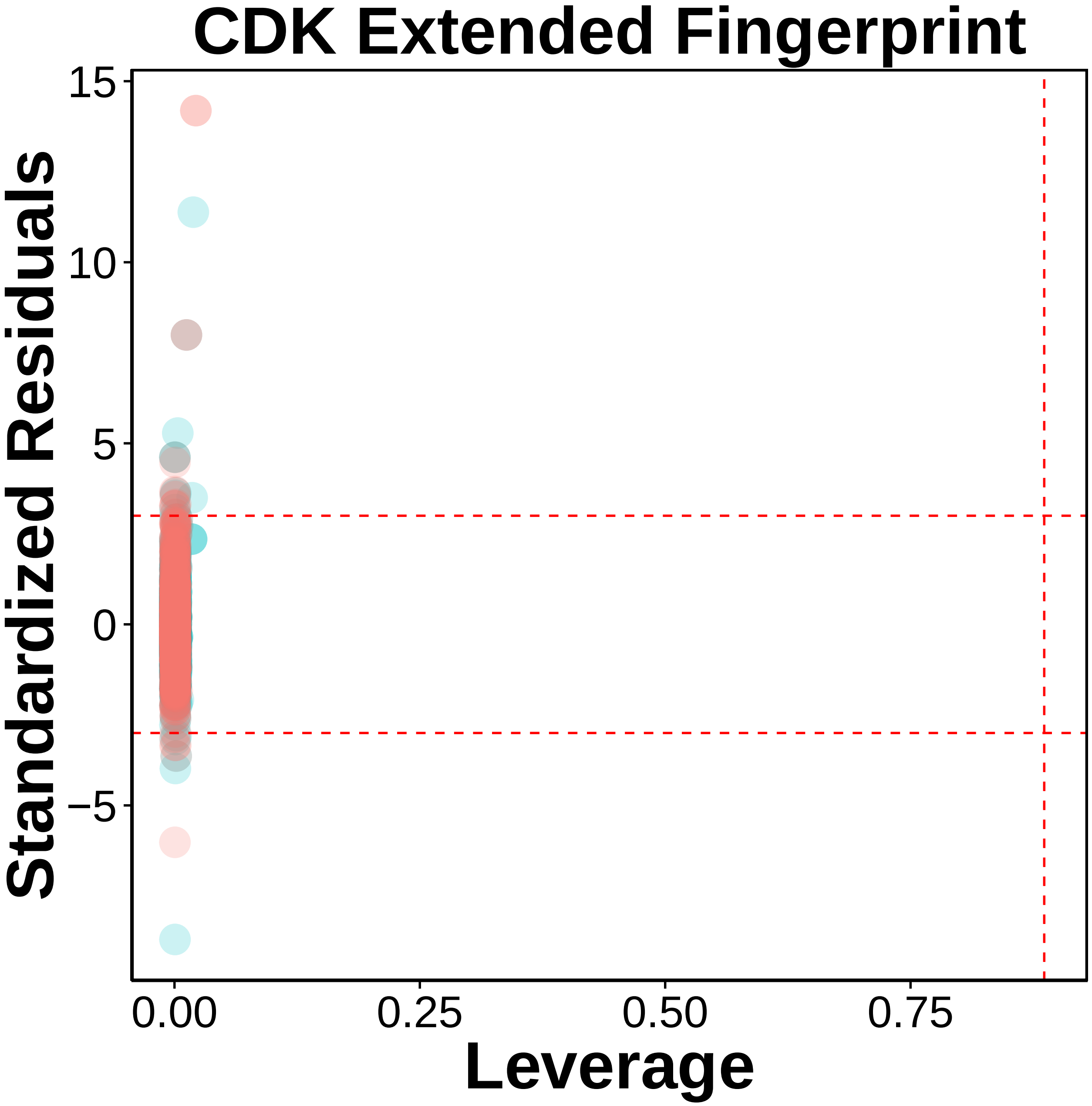

CDK graph only fingerprint

```
input <- readRDS("data.Rds")
df <- input$GraphOnly_FingerPrinter
plot <- plot_william(df, title = "CDK Graph Only Fingerprint")
h <- 3*((dim(df)[2] - 1) + 1) / dim(df)[1]*0.8
plot + geom_vline(xintercept = h, linetype = 2, color = "red")
```

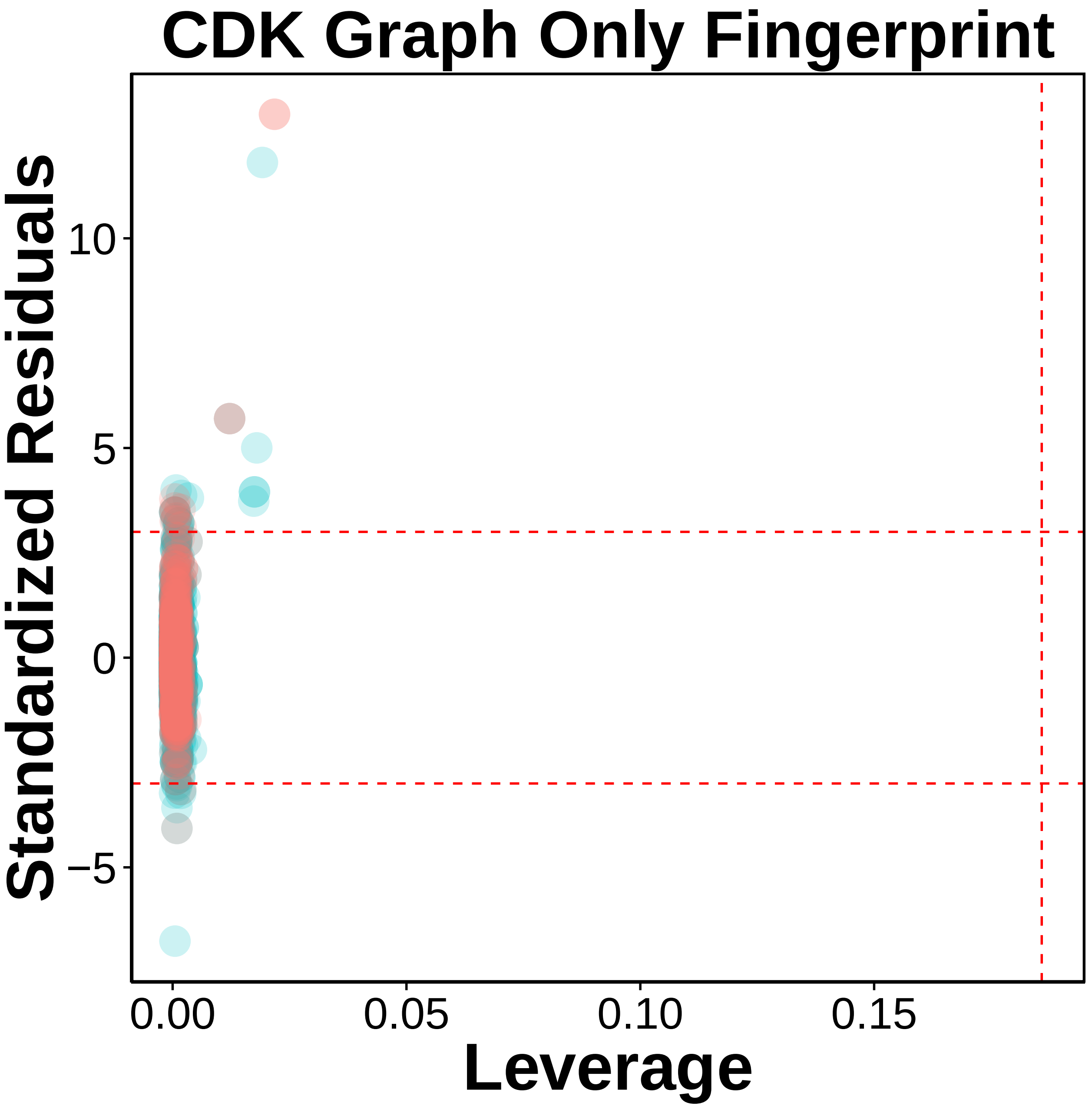

E-State fingerprint

```
input <- readRDS("data.Rds")
df <- input$Estate_FingerPrinter
plot <- plot_william(df, title = "E-State Fingerprint")
h <- 3*((dim(df)[2] - 1) + 1) / dim(df)[1]*0.8
plot + geom_vline(xintercept = h, linetype = 2, color = "red")
```

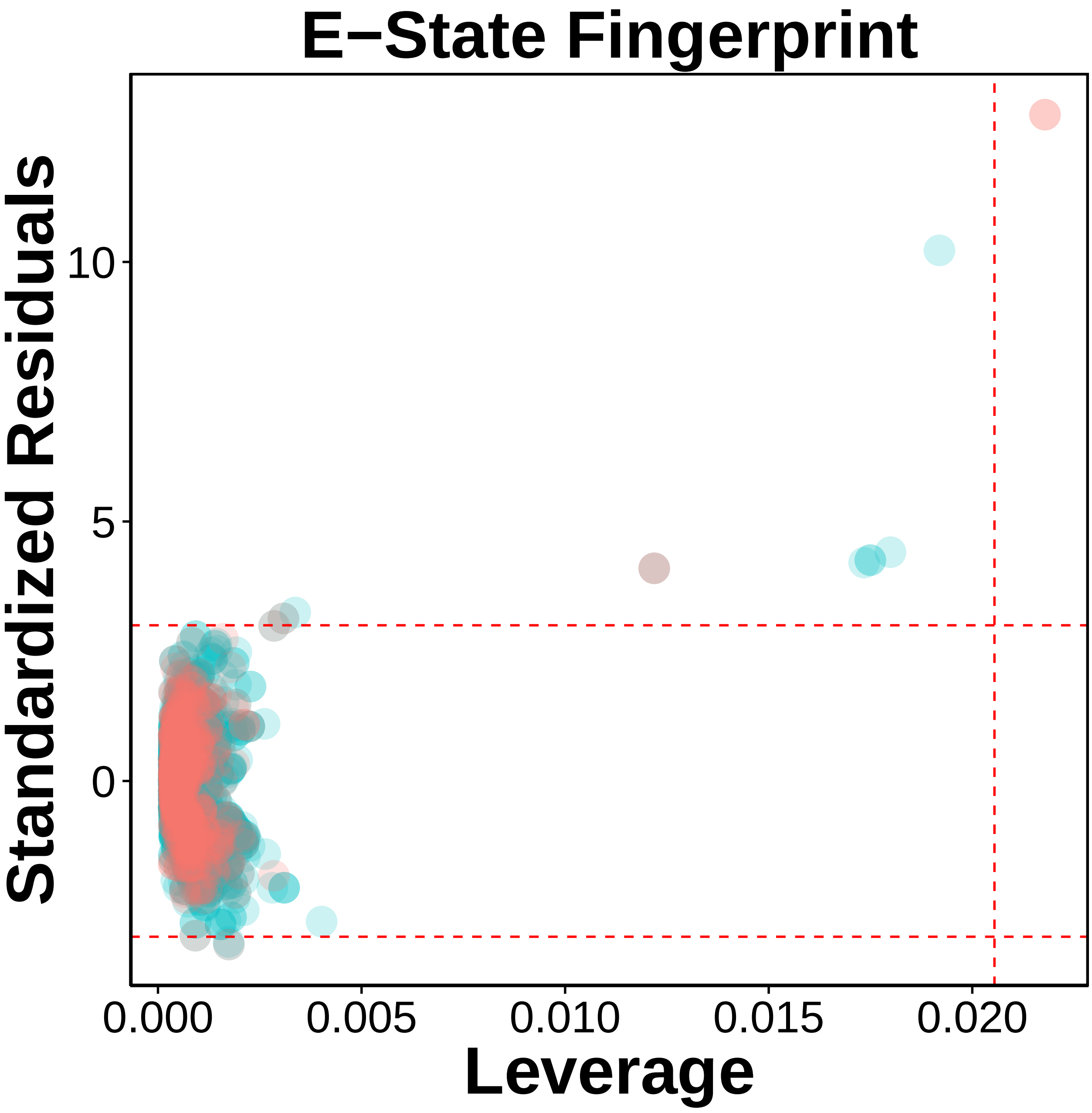

MACCS fingerprint

```
input <- readRDS("data.Rds")
df <- input$MACCS_FingerPrinter
plot <- plot_william(df, title = "MACCS Fingerprint")
h <- 3*((dim(df)[2] - 1) + 1) / dim(df)[1]*0.8
plot + geom_vline(xintercept = h, linetype = 2, color = "red")
```

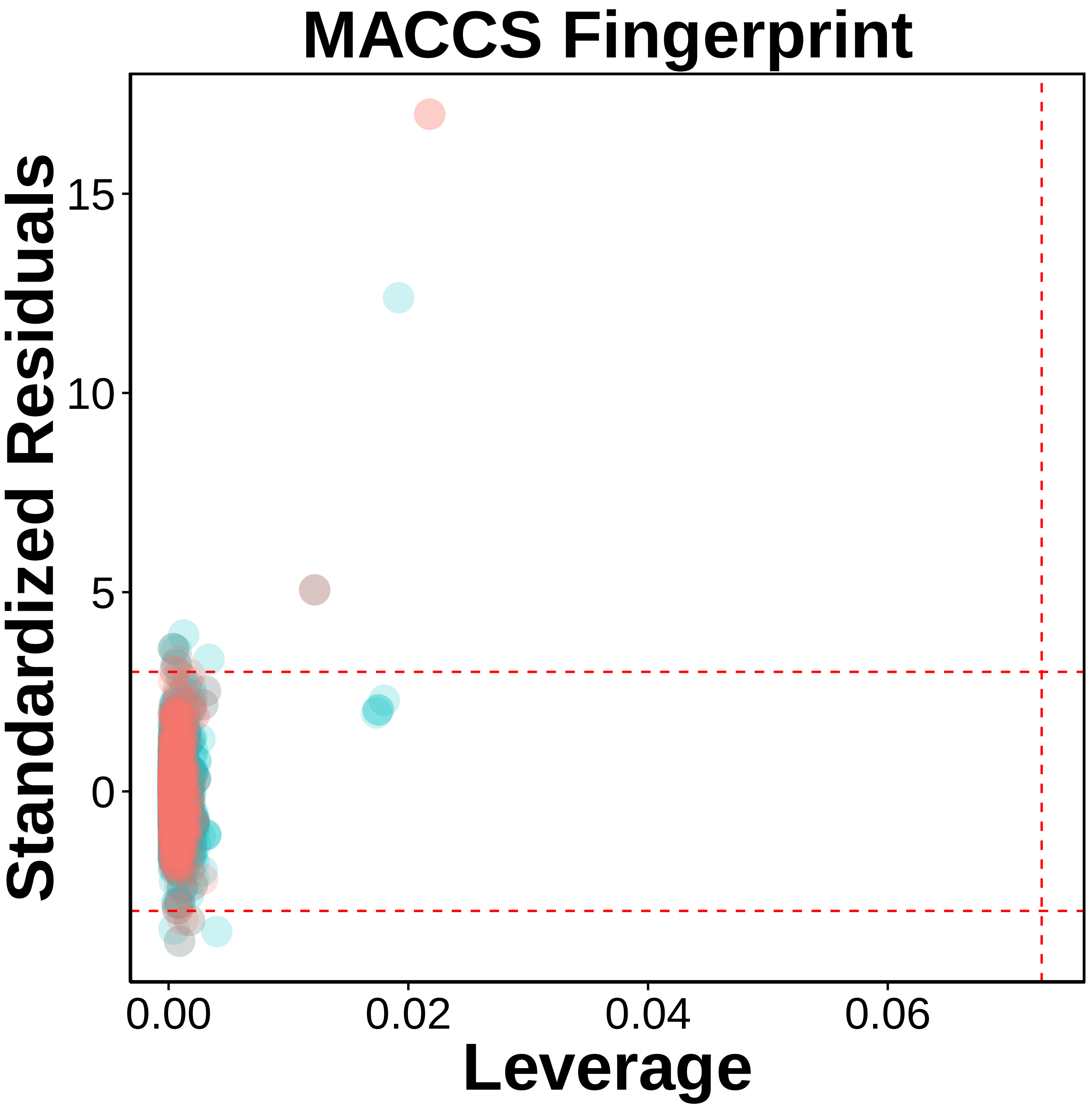

PubChem fingerprint

```
input <- readRDS("data.Rds")
df <- input$Pubchem_FingerPrinter
plot <- plot_william(df, title = "PubChem Fingerprint")
h <- 3*((dim(df)[2] - 1) + 1) / dim(df)[1]*0.8
plot + geom_vline(xintercept = h, linetype = 2, color = "red")
```

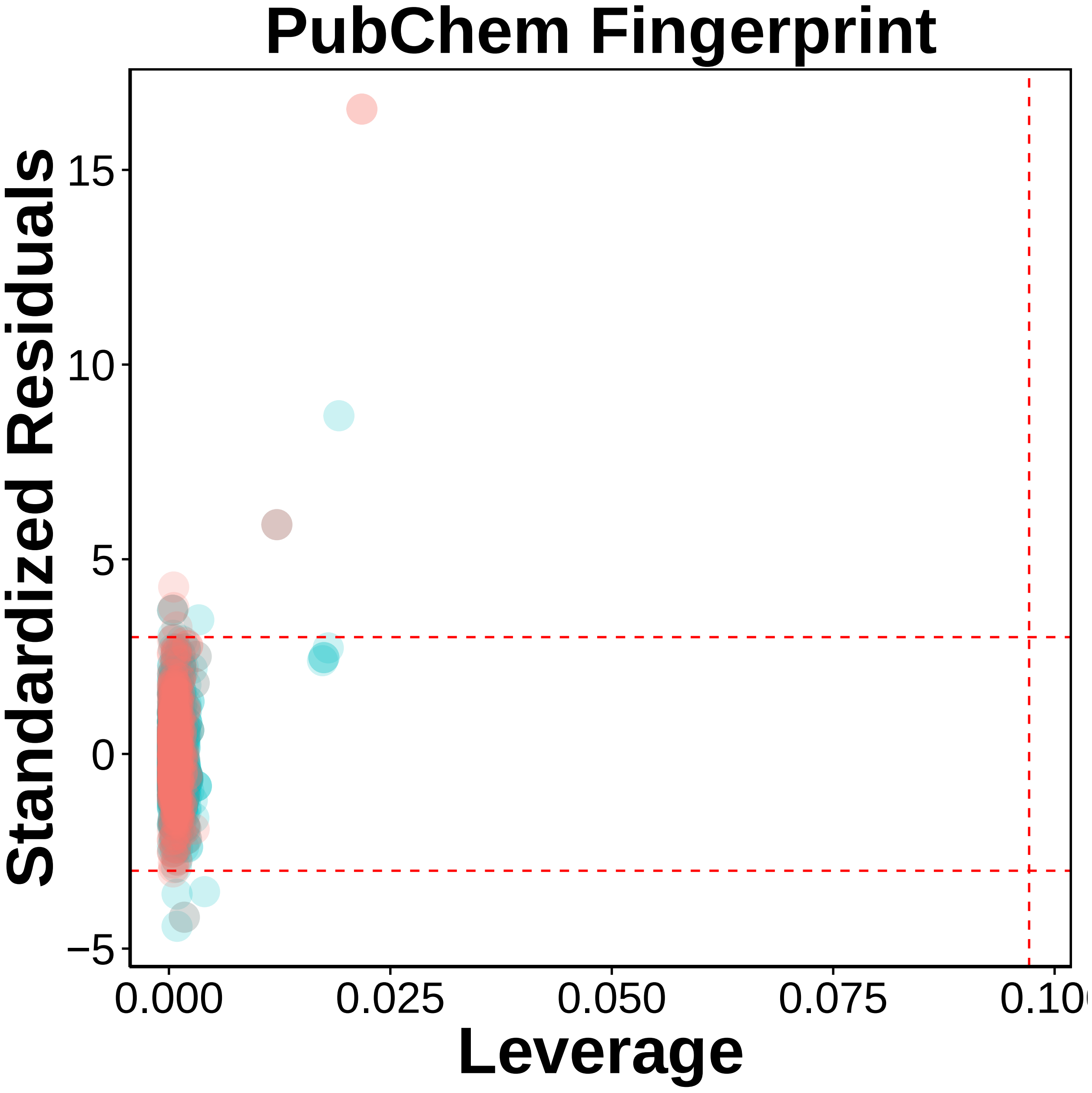

Substructure fingerprint

```
input <- readRDS("data.Rds")
df <- input$Substructure_fingerPrinter
plot <- plot_william(df, title = "Substructure Fingerprint")
h <- 3*((dim(df)[2] - 1) + 1) / dim(df)[1]*0.8
plot + geom_vline(xintercept = h, linetype = 2, color = "red")
```

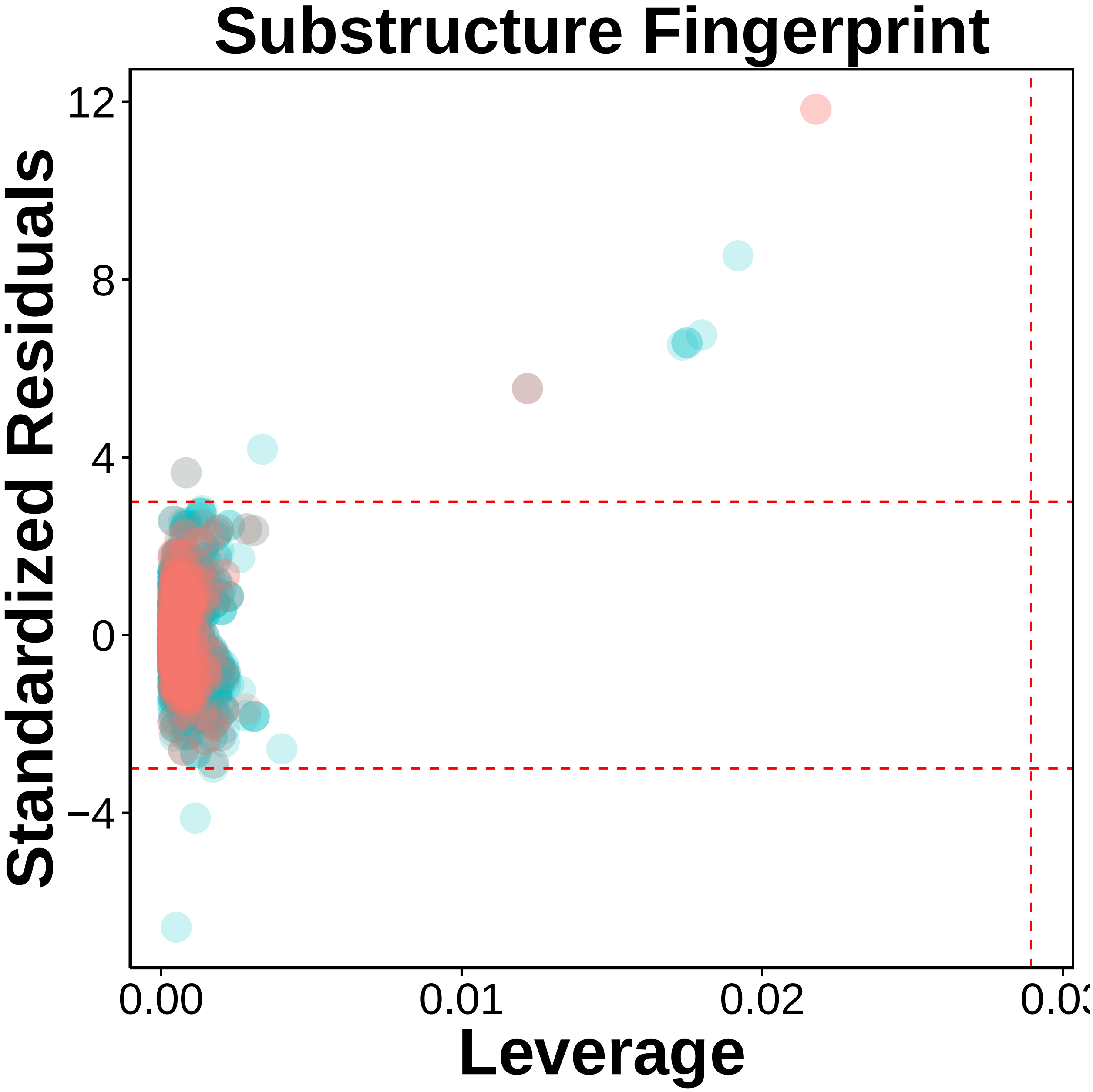

Substructure count

```
input <- readRDS("data.Rds")
df <- input$Substructure_fingerPrintCount
plot <- plot_william(df, title = "Substructure Count")
h <- 3*((dim(df)[2] - 1) + 1) / dim(df)[1]*0.8
plot + geom_vline(xintercept = h, linetype = 2, color = "red")
```

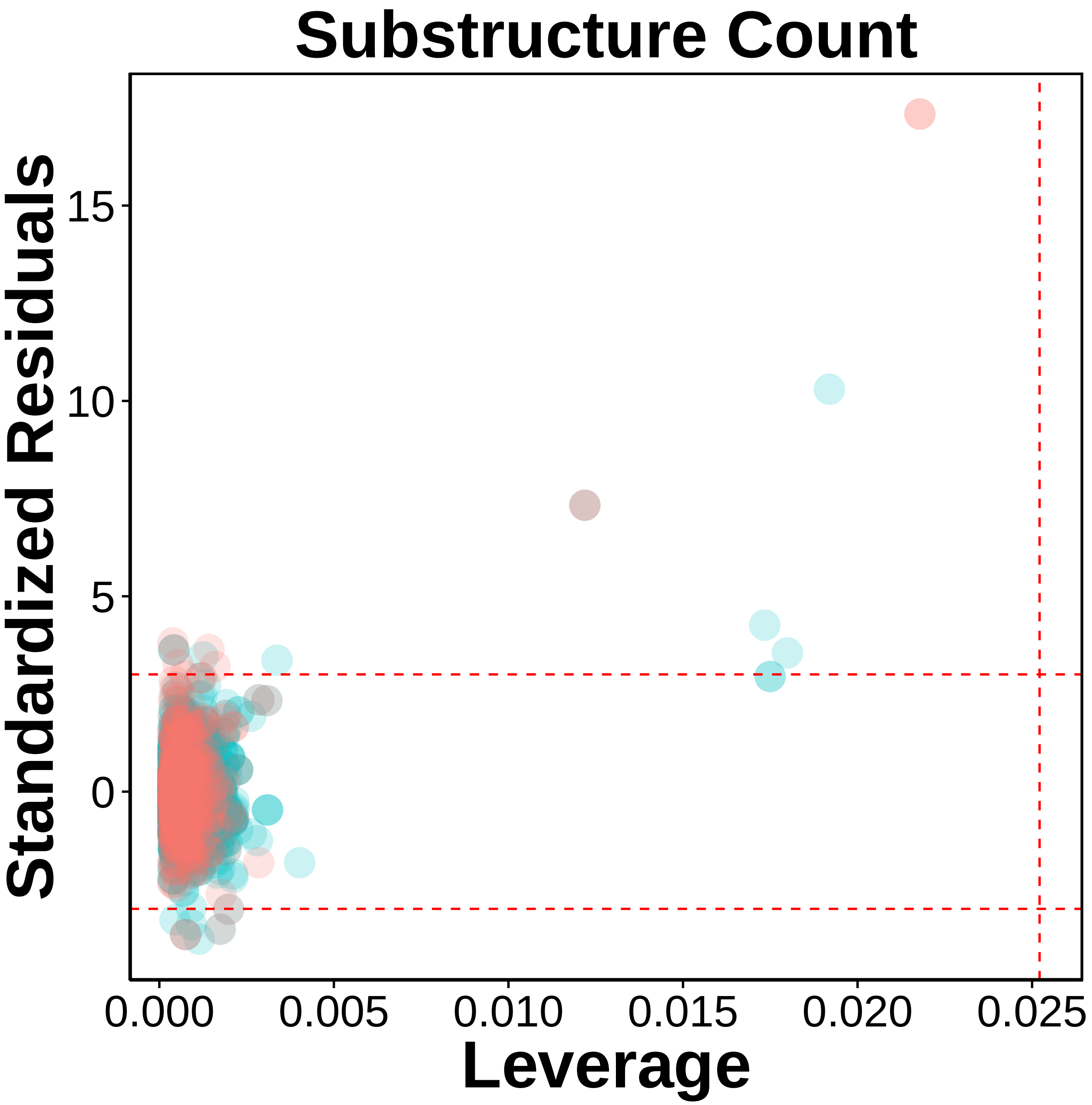

Klekota-Roth fingerprint

```
input <- readRDS("data.Rds")
df <- input$KlekotaRoth_FingerPrinter
plot <- plot_william(df, title = "Klekota-Roth Fingerprint")
h <- 3*((dim(df)[2] - 1) + 1) / dim(df)[1]*0.8
plot + geom_vline(xintercept = h, linetype = 2, color = "red")
```

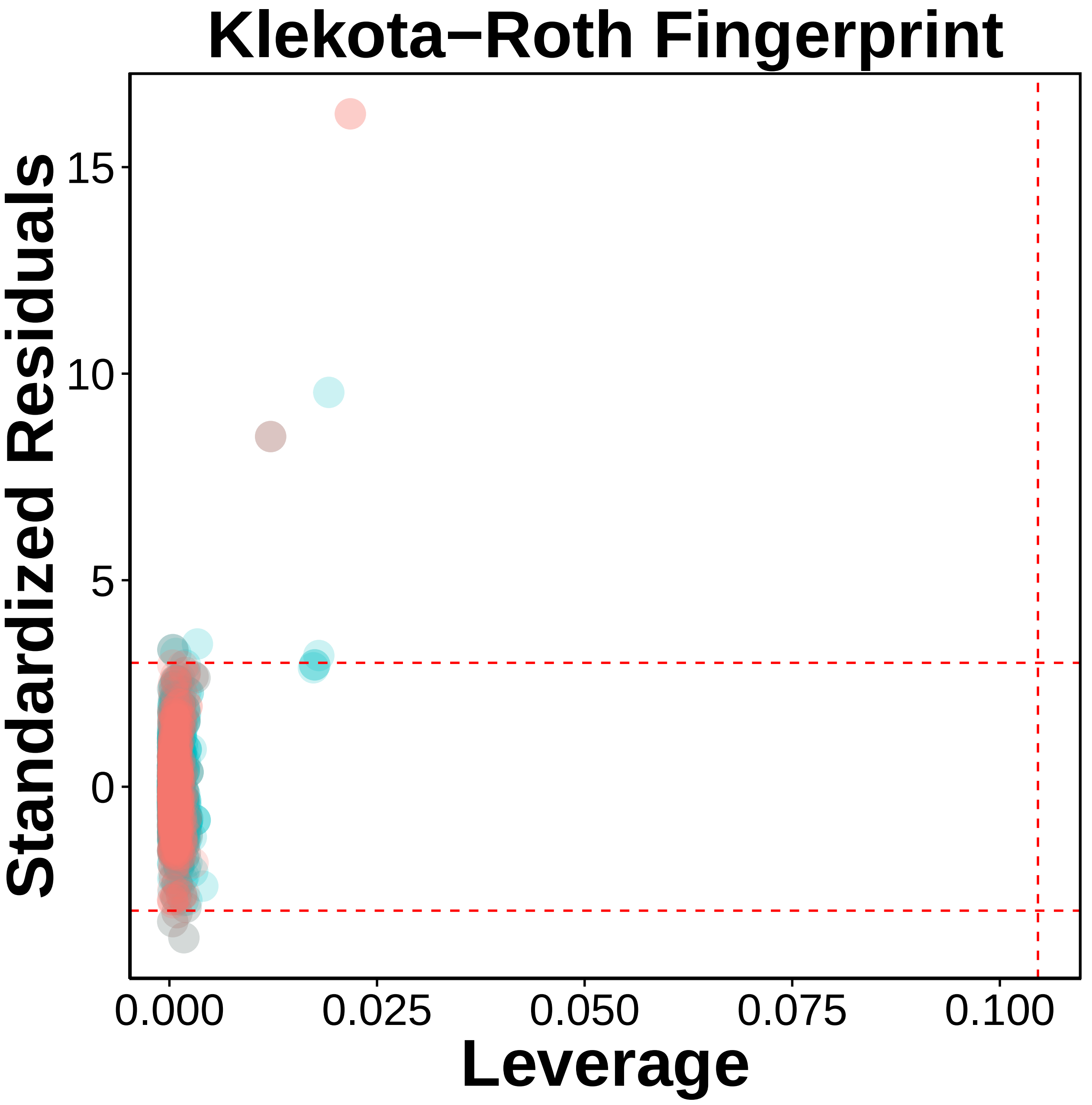

Klekota-Roth count

```
input <- readRDS("data.Rds")
df <- input$KlekotaRoth_FingerprintCount
plot <- plot_william(df, title = "Klekota-Roth count")
h <- 3*((dim(df)[2] - 1) + 1) / dim(df)[1]*0.8
plot + geom_vline(xintercept = h, linetype = 2, color = "red")
```

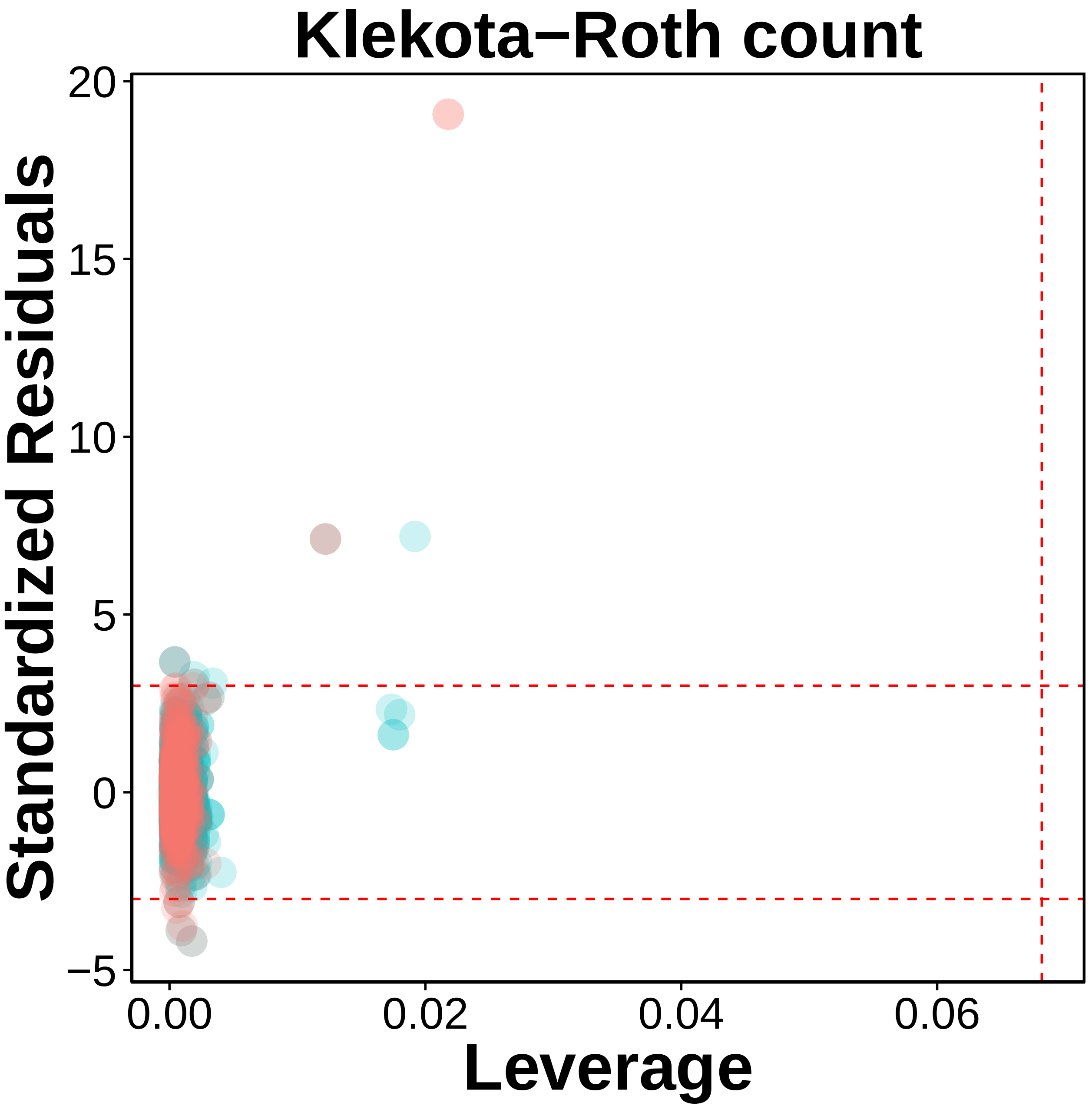

2D atom pairs

```
input <- readRDS("data.Rds")
df <- input$AtomPairs2D_fingerPrinter
plot <- plot_william(df, title = "2D Atom Pairs")
h <- 3*((dim(df)[2] - 1) + 1) / dim(df)[1]*0.8
plot + geom_vline(xintercept = h, linetype = 2, color = "red")
```

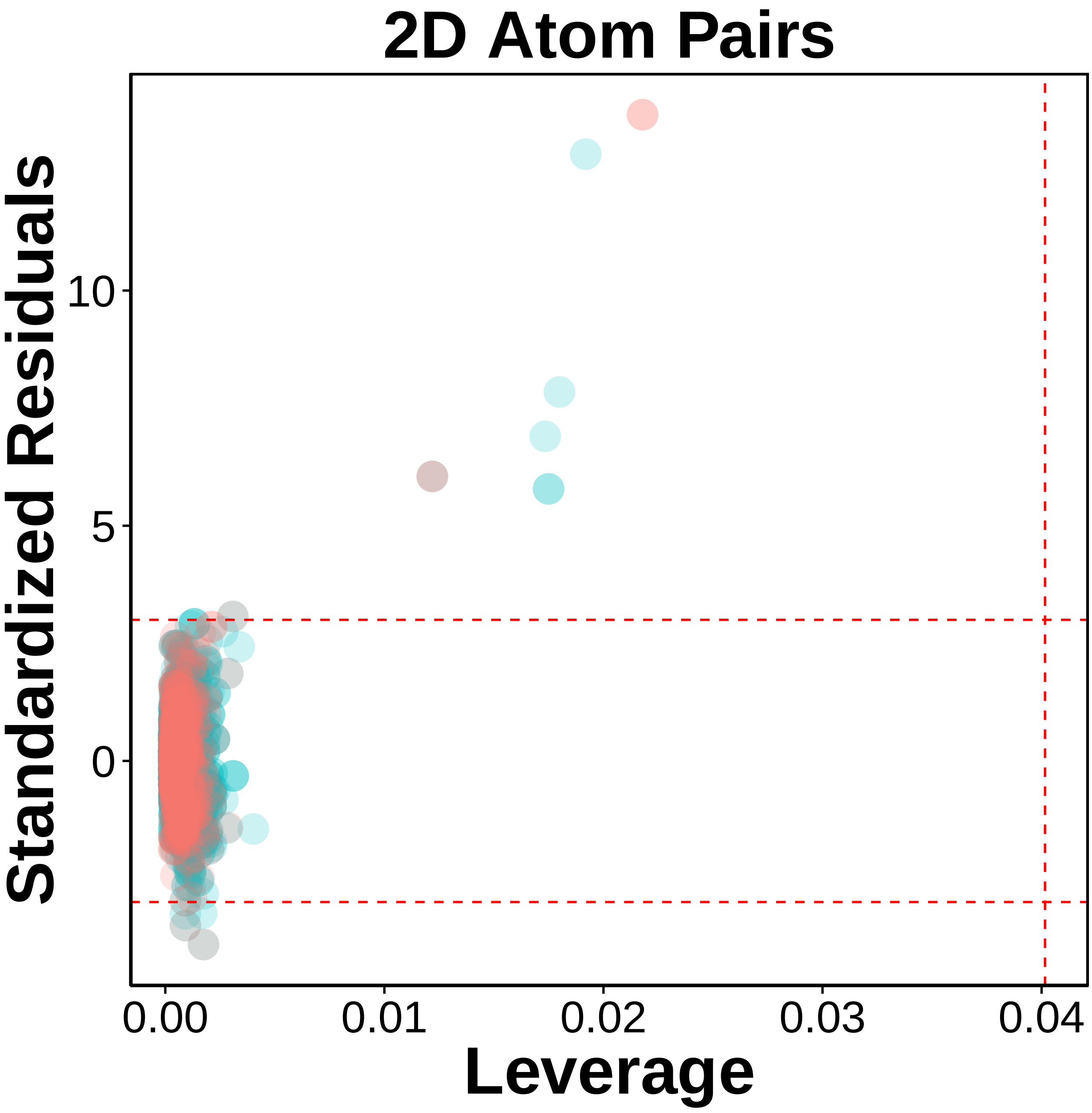

2D atom pairs count

```
input <- readRDS("data.Rds")
df <- input$AtomPairs2D_fingerPrintCount
plot <- plot_william(df, title = "2D Atom Pairs Count")
h <- 3*((dim(df)[2] - 1) + 1) / dim(df)[1]*0.8
plot + geom_vline(xintercept = h, linetype = 2, color = "red")
```

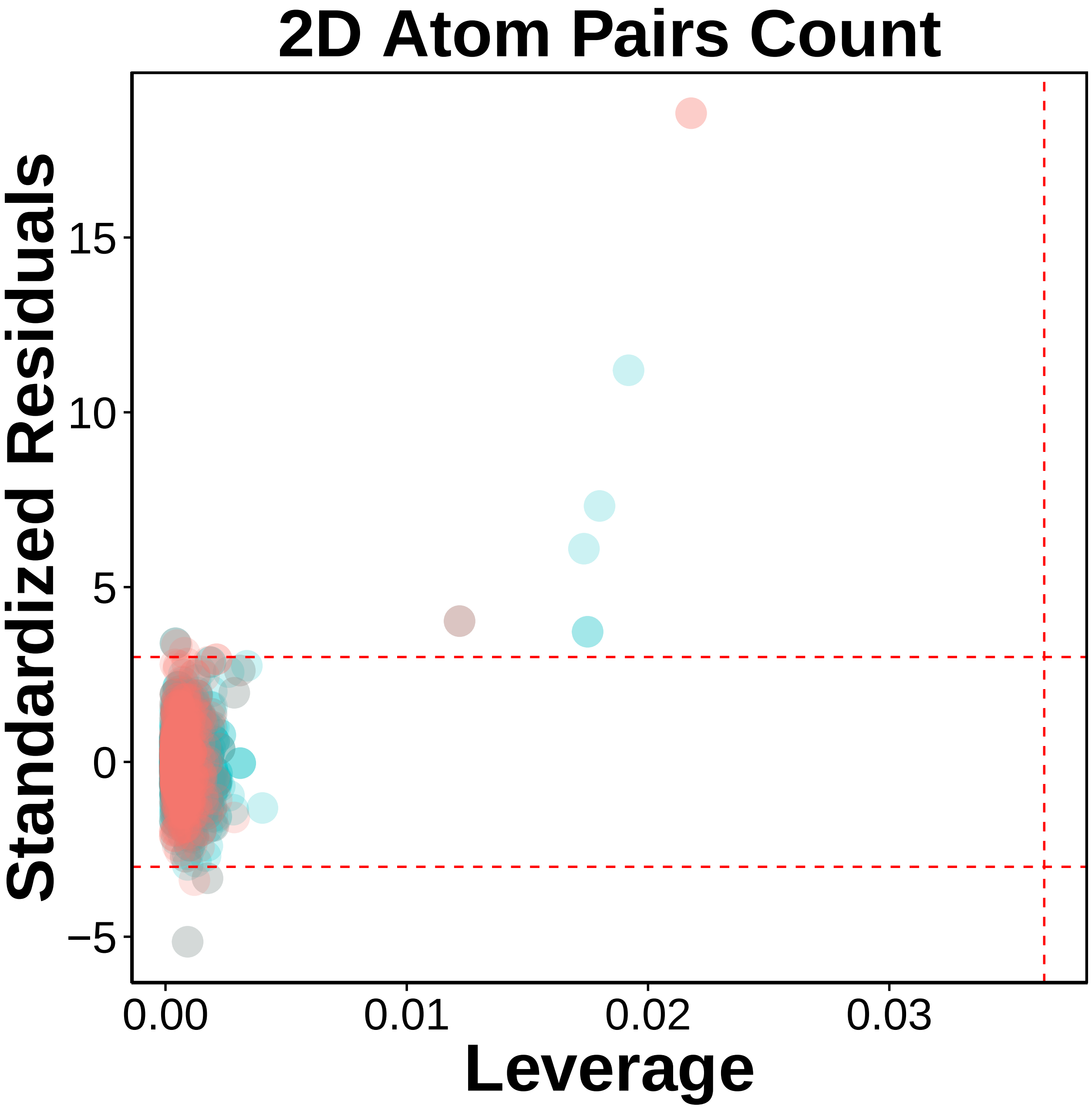

Supplement: Data S2 [file peerj-04-2322-s003.zip › R mark down/William_Plot.pdf]
