# Supplementary figures and images for: Probing the origins of human acetylcholinesterase inhibition via QSAR modeling and molecular docking
Source: PeerJ. 2016 Aug 9;4:e2322. doi: 10.7717/peerj.2322 (PMC4991866; doi:10.7717/peerj.2322)

Predicted  $\text{pIC}_{50}$

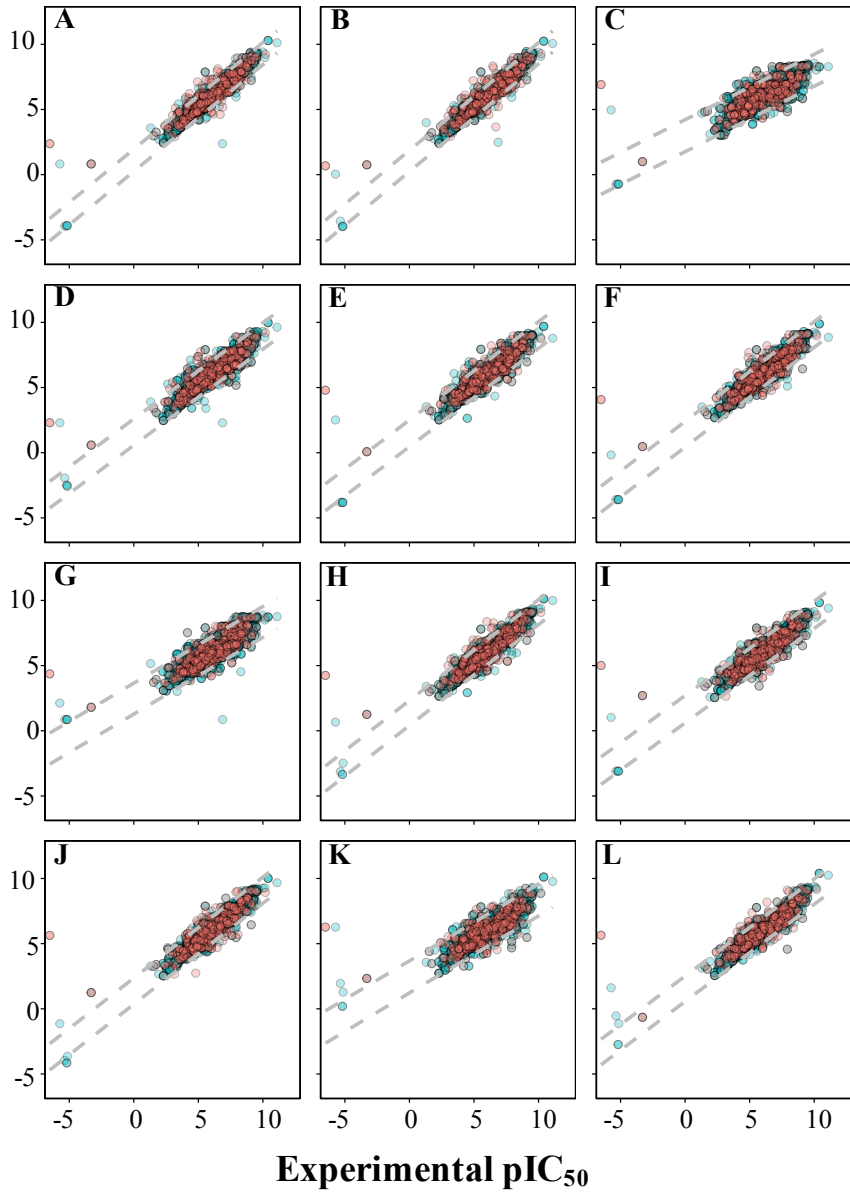

Supplement: Data S2 [file peerj-04-2322-s003.zip › R mark down/Fig_Exp_vs_Pred_Review.pdf]

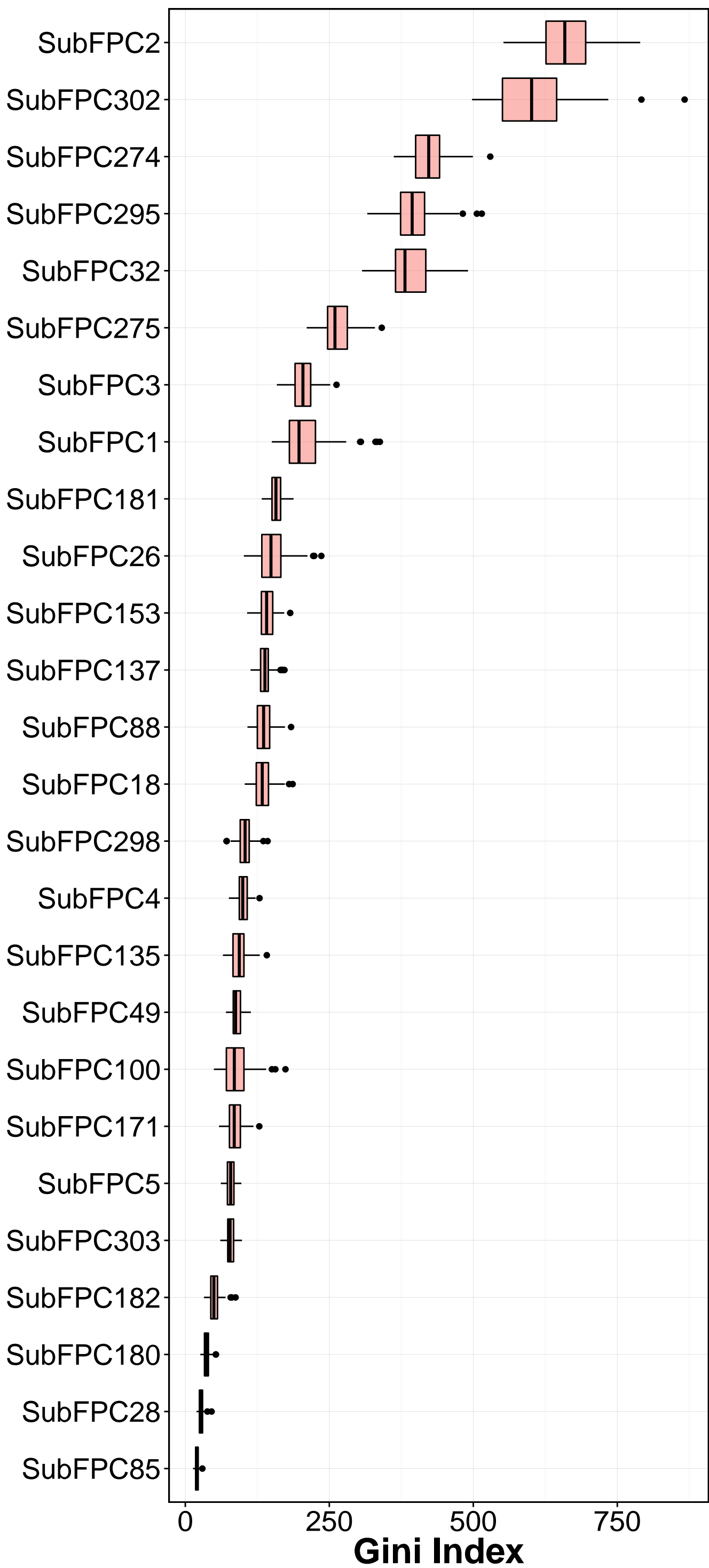

Supplement: Data S2 [file peerj-04-2322-s003.zip › R mark down/Fig_Feature_Importance_Review.pdf]

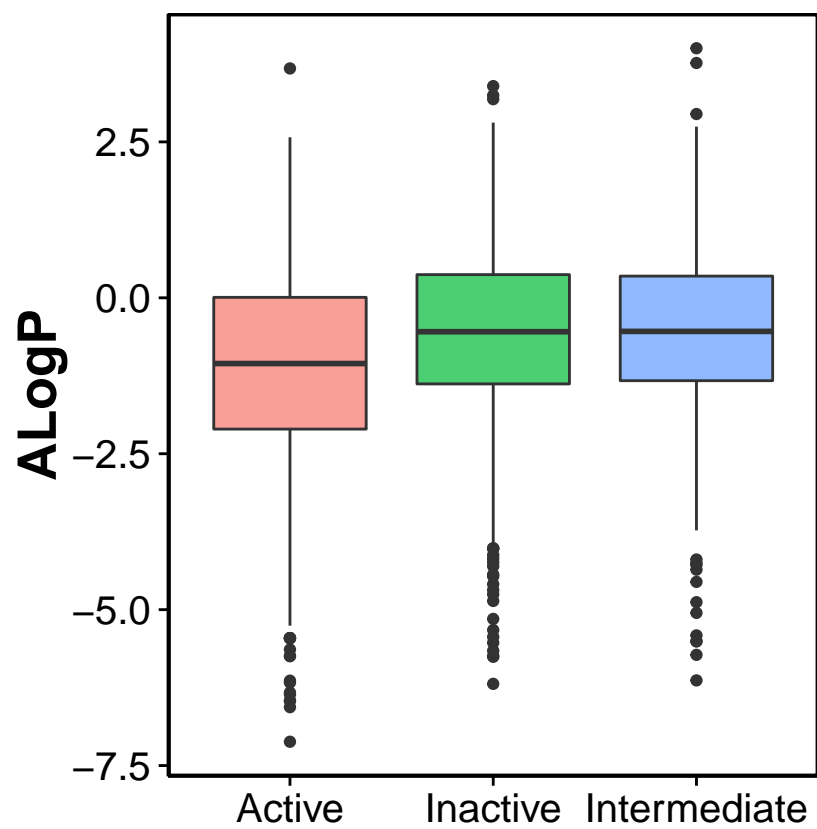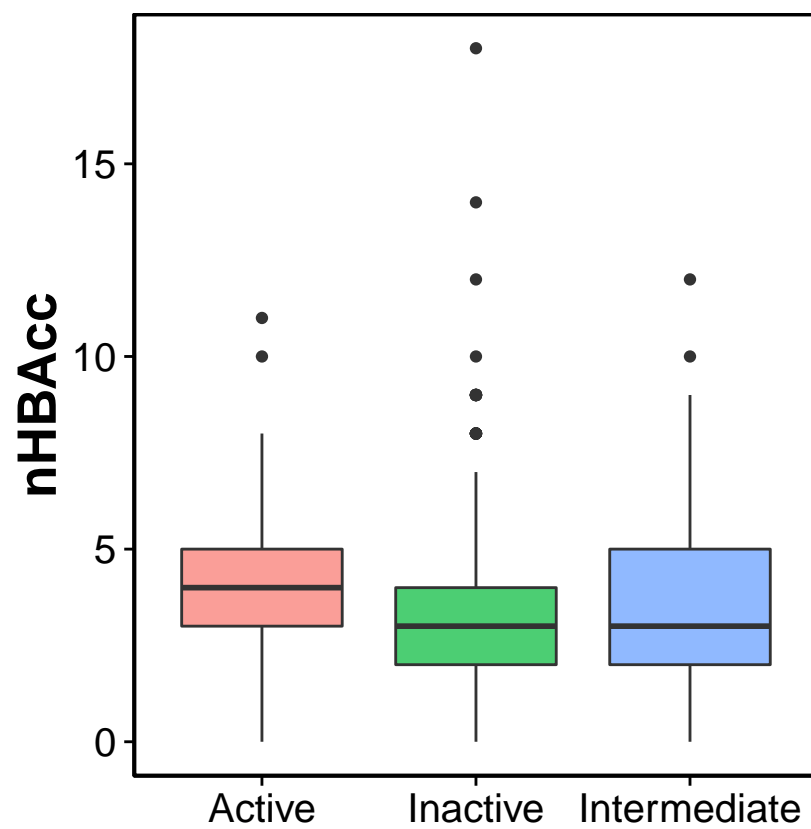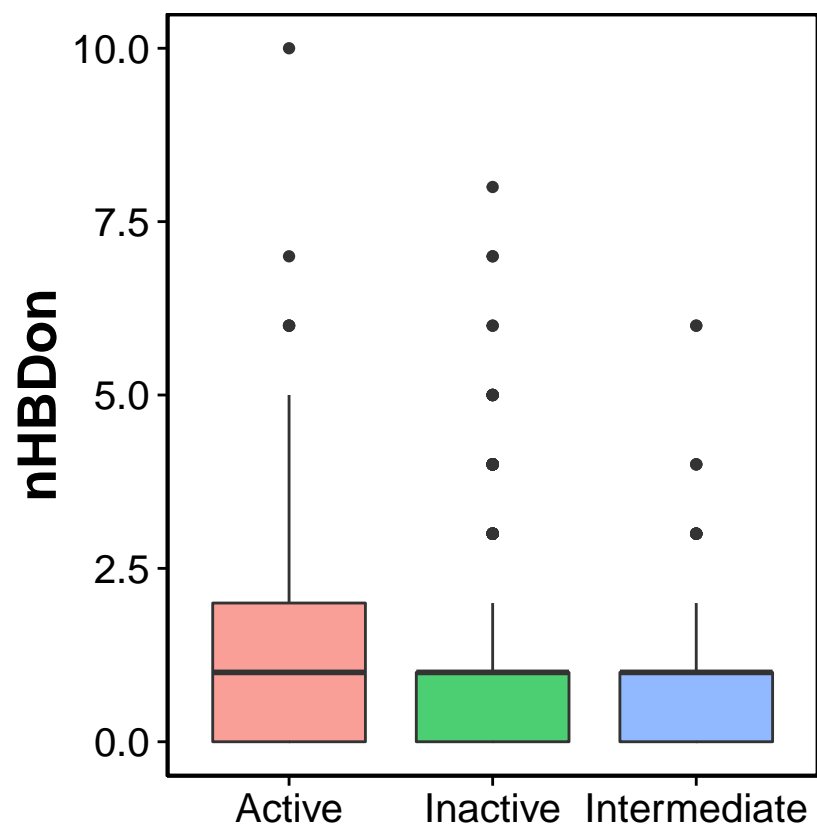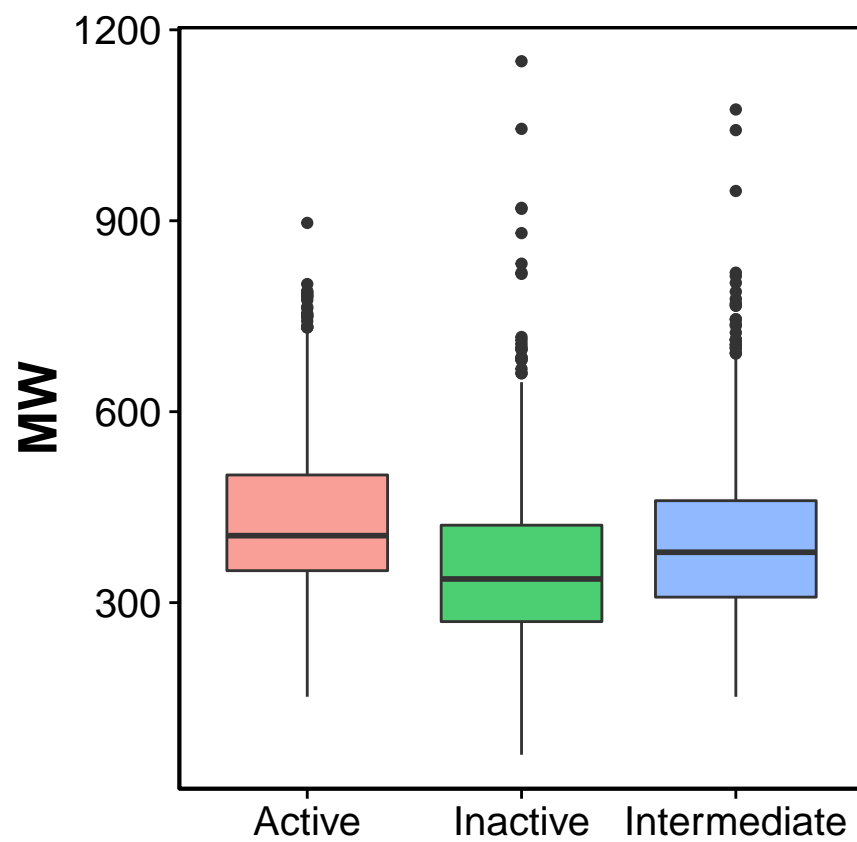

Supplement: Data S2 [file peerj-04-2322-s003.zip › R mark down/Fig_Lipinski_Box_Plot_Review.pdf]

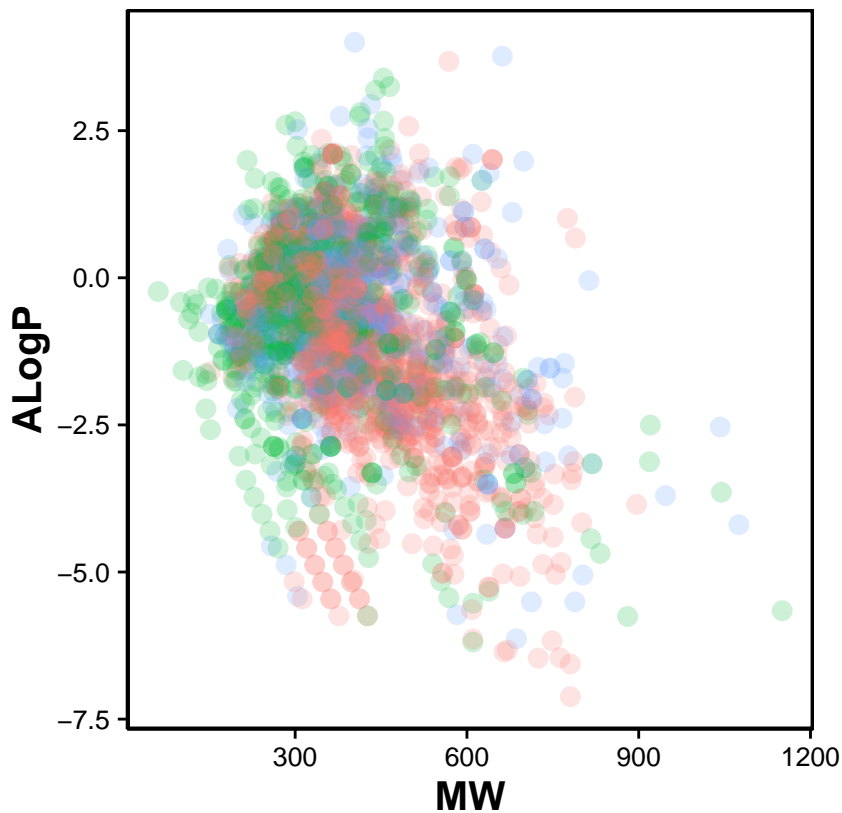

Supplement: Data S2 [file peerj-04-2322-s003.zip › R mark down/Fig_MW_vs_ALogP_Review.pdf]
