# Supplementary material for: Probing the origins of human acetylcholinesterase inhibition via QSAR modeling and molecular docking
Source: PeerJ. 2016 Aug 9;4:e2322. doi: 10.7717/peerj.2322 (PMC4991866; doi:10.7717/peerj.2322)
Supplement: Data S2 [file Fig_William_Plot.pdf › R mark down/Fig_William_Plot.pdf]

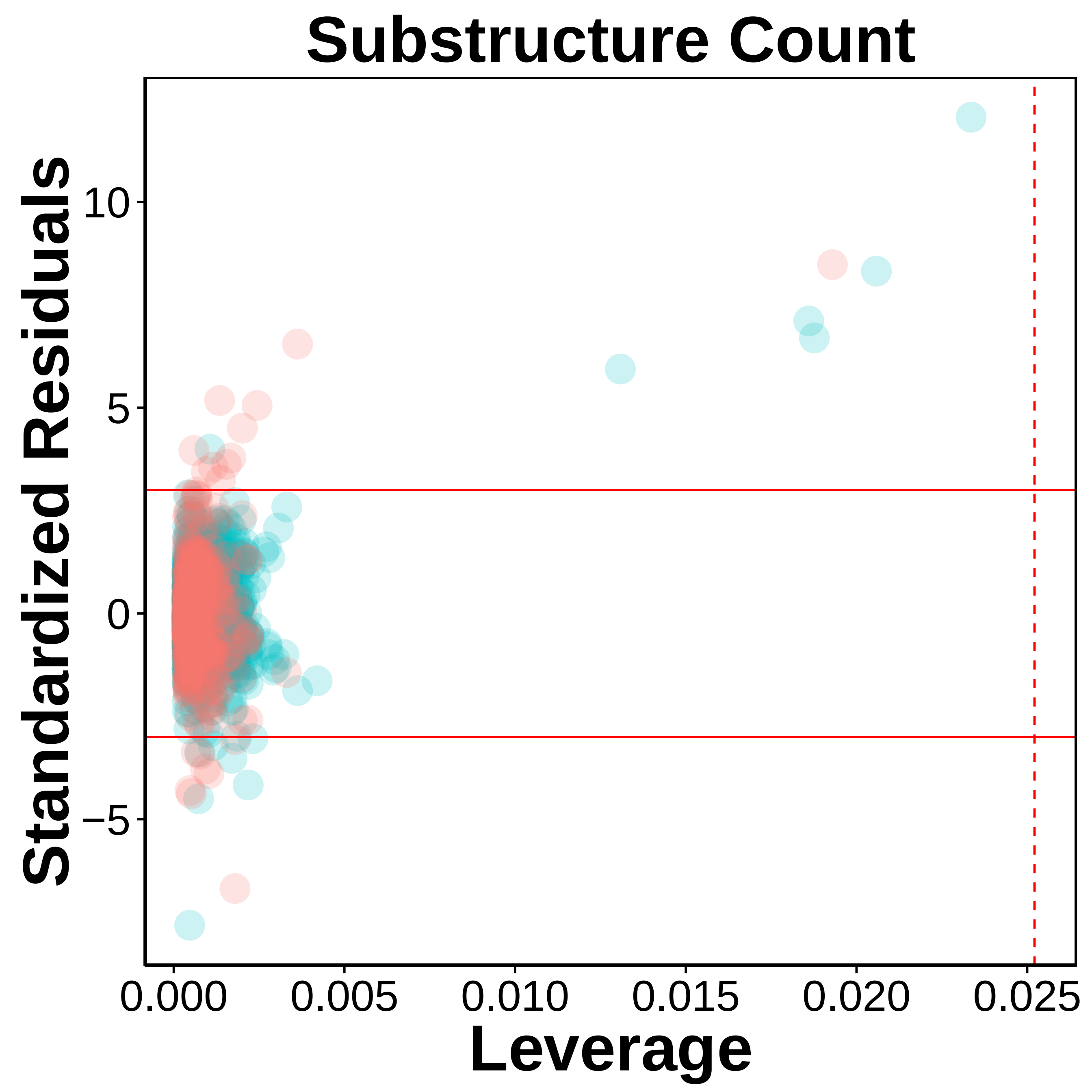
Substructure Count
Standardized Residuals
10
5
0
−5
0.000
0.005
0.010
0.015
0.020
0.025
Leverage
